# Integrated Sensing and Communication for 6G V2X Networks: A Comprehensive Survey of Architectures, Security, and Multi-Modal AI

**DOI:** 10.3390/s26175653

**Published:** 2026-09-05

**Authors:** Furkan Şen, Arif Basgumus, Mustafa Namdar

**Affiliations:** 1 Department of Electrical and Electronics Engineering, Bursa Uludag University, Bursa 16059, Türkiye; 512305004@ogr.uludag.edu.tr; 2 Research and Development Department, TCI Cabin Interior Systems, Istanbul 34906, Türkiye; 3 Department of Electrical and Electronics Engineering, Kutahya Dumlupinar University, Kutahya 43100, Türkiye; mustafa.namdar@dpu.edu.tr

**Keywords:** 6G wireless networks, integrated sensing and communication (ISAC), multi-modal artificial intelligence, physical layer security, vehicle-to-everything (V2X)

## Abstract

Sixth-generation (6G) vehicular systems are expected to provide extreme reliability, ultra-low latency, and high data rates while simultaneously enabling accurate and timely perception of vehicles, road users, and surrounding environments. Conventional vehicle-to-everything (V2X) systems treat communication and environmental sensing separately and therefore fall short in dynamic, dense, and safety-critical driving scenarios. Integrated sensing and communication for V2X (ISAC-V2X) addresses this limitation by sharing hardware and spectrum for dual-functional operation. This survey consolidates the state of the art across eight thematic areas: fundamentals and taxonomy; vehicular channel models and sensing metrics; physical-layer techniques, including waveform design, beamforming, reconfigurable intelligent surfaces, non-orthogonal multiple access, and rate-splitting multiple access; security and privacy; networked and cell-free ISAC with multi-access edge computing; multi-modal perception using radar, LiDAR, camera, and radio-frequency data; artificial intelligence and ML for channel, beam, target, and sensing-data processing; and future research directions. Five consolidated research gaps are synthesized from the reviewed literature: end-to-end frameworks for networked ISAC-V2X under high mobility; unified multi-modal AI-native architectures for joint sensing, communication, localization, and security; incomplete standardization and interoperability; limited validation, testbeds, and reproducible benchmarks; and scalability, robustness, and cross-domain optimization. This survey concludes with standardization and testbed recommendations and presents a roadmap toward deployable 6G ISAC-V2X systems.

## 1. Introduction and Motivation

Next-generation (6G) wireless networks are expected to deliver service targets that exceed 5G by a wide margin. Among the most challenging application domains, vehicle-to-everything (V2X) communication and sensing must meet strict reliability, sub-millisecond latency, high throughput, and massive connectivity requirements in rapidly changing radio environments [1,2,3]. Advanced V2X services span ultra-reliable low-latency communication (URLLC) for safety-critical control, enhanced mobile broadband (eMBB) for perception and infotainment traffic, and massive machine-type communication (mMTC) for dense telemetry among vehicles, roadside units (RSUs), and infrastructure [2,4]. Cooperative-intelligent transport systems (C-ITSs) and connected autonomous driving (CAD) further tighten the requirements on end-to-end latency, link reliability, and localization accuracy, calling for closer coupling of communication, sensing, computing, and control in future 6G V2X deployments [5,6].

Dedicated short-range communications (DSRCs) and cellular V2X (C-V2X) established the first standardized pathways for vehicle-to-vehicle (V2V), vehicle-to-infrastructure (V2I), and vehicle-to-network (V2N) connectivity, but both families fall short of 6G-level expectations [2,3]. IEEE 802.11p-based DSRC relies on contention-based access; under high traffic density, collision probability grows quickly and URLLC guarantees become difficult to sustain [3]. V2X protocol stacks today silo localization, radar sensing, and traffic bearers. Resource pools are rarely optimized jointly, and cues that bridge modalities go unused: echo traces from onboard radar could refine beam selection, while map-derived priors could steer slot or subcarrier assignment [7,8]. Consequently, DSRC and C-V2X as deployed today fall short of the simultaneous goals of dependable autonomous safety, shared situational awareness among vehicles, and continuous wide-area service articulated in 6G research roadmaps [2,4].

Integrated sensing and communication (ISAC) targets this shortfall by letting radar-comparable sensing and payload delivery share transceivers, spectrum allocations, and waveform design [1,9]. In addition to improving spectrum efficiency, reducing hardware costs, and simplifying deployment, codesign of transmit waveforms, receiver processing, and resource allocation opens design degrees of freedom that neither function can support on its own [10,11]. Communication-centric ISAC prioritizes the throughput and reliability yet uses sensing to refine channel state information (CSI), predict blockages, and manage beams; sensing-centric ISAC inverts that priority, embedding payload data into radar-like waveforms with minimal loss in detection range, resolution, or robustness [1]. Either approach avoids the rigid band splitting and duplicated radio chains that characterize separate radar and communication installations, a practical advantage in spectrum-constrained vehicular networks [5,9].

At the physical layer, ISAC spans a broad codesign space in which waveforms, beamforming, and resource allocation are tuned against both communication and sensing figures of merit. For instance, reconfigurable intelligent surface (RIS)-assisted ISAC formulations expose the rate–accuracy trade-off in a single optimization problem by jointly optimizing waveforms and discrete phase shifts to suppress multi-user interference while satisfying Cramér–Rao bound (CRB) constraints on direction-of-arrival estimation [10]. After active arrays and passive phase profiles are tuned together under linked transmit-power and sensing-accuracy ceilings, rate-splitting multiple access (RSMA) stacked on RIS has improved weighted energy efficiency (WEE) [11]. These kinds of designs perform better than pipelines that adjust radar and communication independently, which is important both in macro cells and on the road: faithful scene sensing and reliable connectivity within small latency margins are assumed by V2X safety logic [3,7].

**Figure 1:**
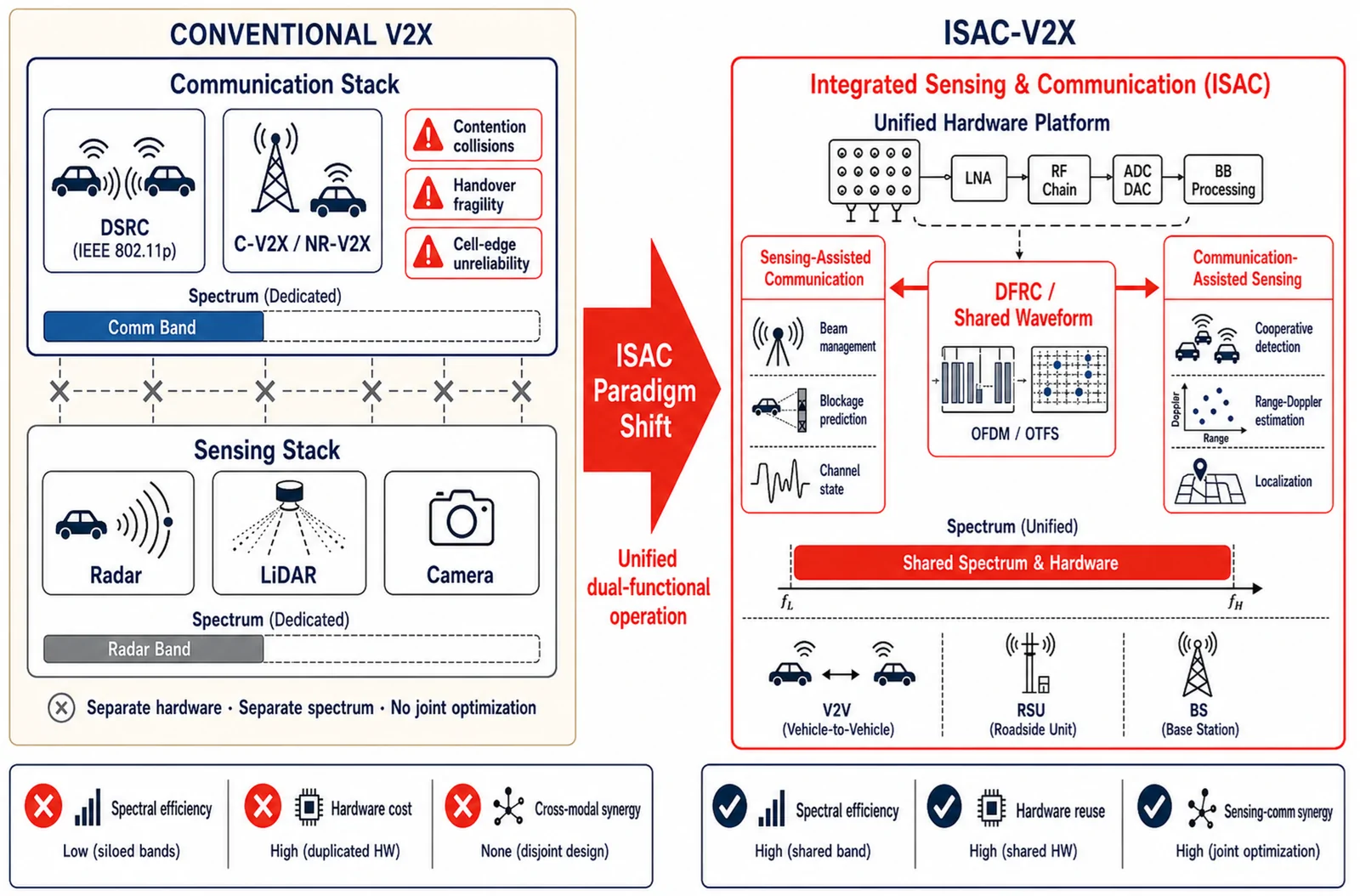
Comparison of conventional V2X architectures with the ISAC-V2X paradigm, highlighting shared hardware, spectrum, and dual-functional sensing–communication operation.

Automotive deployments are a strong fit for ISAC because cabins and chassis already host radar, LiDAR, imagers, and several radio modules, all operating over fast time-varying channels with heavy shadowing and blockage [7,8,12]. ISAC-enabled V2X can therefore harvest paired sensing and communication payoffs in the same air interface. Sensing-assisted communication uses radar or fused perception to anticipate blockages, guide beam and handover decisions, and allocate resources with geometric context, improving link stability and lowering control signaling [6,8]. Communication-assisted sensing reuses data waveforms, such as orthogonal frequency-division multiplexing (OFDM) or orthogonal time frequency space (OTFS), for target detection, range-Doppler estimation, and cooperative localization beyond a single vehicle’s line-of-sight (LoS), extending perception range and easing occlusions in congested traffic [7,9]. This bidirectional coupling defines the ISAC-V2X paradigm examined in the present survey. Figure 1 contrasts conventional separated sensing–communication architectures with the unified ISAC-V2X paradigm.

Despite rapid progress in ISAC and V2X, existing studies still reveal two closely related limitations. Networked ISAC-V2X lacks unified designs that jointly account for sensing, communication, resource allocation, mobility, and interference at fleet scale [2,3,5,7,9,13]. At the same time, multi-modal approaches combining RF, radar, LiDAR, camera, and AI/ML are rarely integrated with communication reliability, security, privacy, and robustness requirements [1,6,8,14,15,16]. These limitations motivate the broader set of research gaps formalized below.

Although recent surveys have addressed ISAC fundamentals, vehicular communications, sensing-assisted V2X, artificial intelligence (AI) and machine learning (ML)-enabled wireless systems, and related enabling technologies [1,3,6,7,8,15,17,18,19,20,21], the literature remains fragmented across communication, sensing, networking, security, and intelligent perception. Accordingly, five consolidated research gaps are synthesized from the reviewed literature. G1 concerns the lack of end-to-end networked ISAC-V2X frameworks for jointly optimizing sensing, communication, resource allocation, and cooperation under realistic vehicular mobility and interference [5,7,9,13]. G2 reflects the absence of unified multi-modal AI-native architectures that jointly exploit radio frequency (RF), radar, LiDAR, and camera information while accounting for communication reliability, security, and robustness [1,6,8,14,15,16]. G3 concerns incomplete standardization and interoperability across cellular, sidelink, sensing, and cooperative perception interfaces [1,22]; G4 reflects the limited availability of large-scale experimental validation, multi-node testbeds, open multi-modal datasets, and reproducible benchmarks [8,14,23]; and G5 concerns the scalability and robustness of ISAC-V2X as the number of vehicles, sensing modalities, infrastructure nodes, and optimization variables increases [24]. Together, these gaps motivate a unified 6G ISAC-V2X framework spanning physical-layer design, security, networked operation, multi-modal AI, and practical deployment.

**Figure 2:**
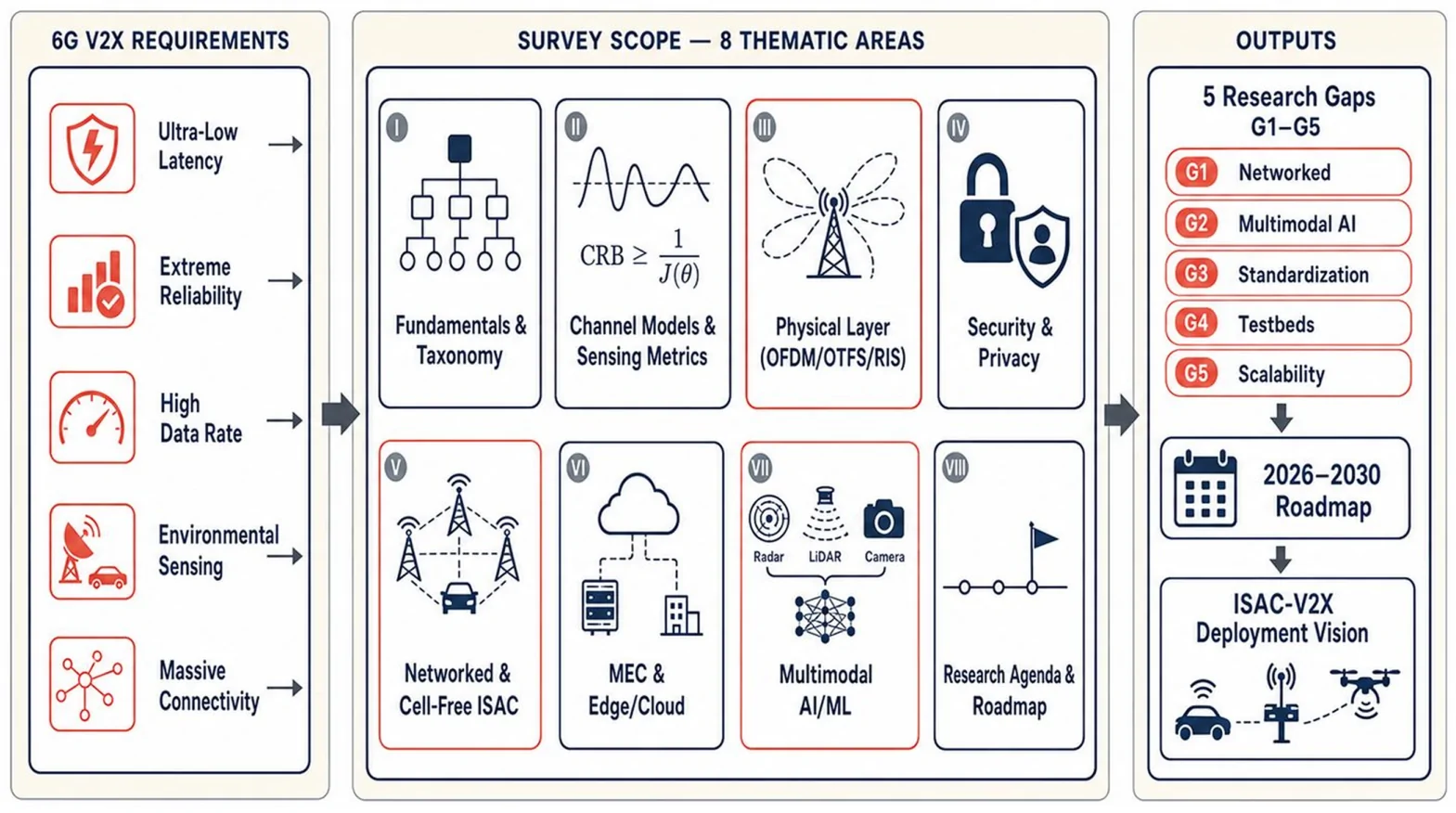
Visual map of ISAC-V2X survey content spanning Section 2, Section 3, Section 4, Section 5, Section 6 and Section 7, merged research gaps G1–G5, and the 2026–2030 outlook in Section 8.

With these five interconnected gaps in view, this survey adopts a 6G-oriented, ISAC-centric perspective on V2X and organizes the literature across architectures, physical-layer techniques, networked operation, multi-modal AI, security, and deployment considerations [1,3,5,6,7,8,9,13,14]. Section 2, Section 3, Section 4, Section 5, Section 6 and Section 7 develop these themes, while Section 8 consolidates G1–G5 and the 2026–2030 roadmap. Figure 2 provides a high-level view of the ISAC-V2X framework considered throughout this survey.

To further clarify the positioning of this survey, Table 1 compares its scope with representative recent reviews in closely related areas. Existing surveys generally focus on selected dimensions of the broader ISAC-V2X ecosystem, including general ISAC, V2X communications, multi-modal sensing, physical-layer security, and AI/ML-enabled sensing–communication integration [1,3,6,7,8,15,17]. In contrast, the present survey jointly covers ISAC-V2X architectures, vehicular channel and sensing models, physical-layer techniques, security and privacy, networked and cell-free operation, multi-modal AI/ML, standardization, testbeds, scalability, and a deployment-oriented roadmap within a unified framework.

Ma et al.’s thorough system-level analysis of ISAC-enabled vehicle networks, which focuses on resource allocation, beam management, RIS-assisted designs, and security and privacy challenges, is one of the most closely related recent studies [18]. Scalability, modeling, hardware flaws, deployment, and standardization issues are also covered in their work. The current survey, on the other hand, is more concerned with networked and cell-free ISAC, vehicular ISAC channel models, multi-modal perception combining RF, radar, LiDAR, and camera data, AI/ML-enabled sensing and communication, standardization-specific advancements, experimental validation, and repeatable benchmarking. As a result, while the current study reorganizes and expands these difficulties within a more comprehensive multi-modal, networked, and deployment-oriented ISAC-V2X framework, several aspects of the research challenges outlined in G1–G5 overlap with issues previously reported by [18].

Keyword combinations pertaining to ISAC, V2X, 6G vehicular networks, sensing, security, and AI/ML were used to search major academic databases, such as IEEE Xplore, Web of Science, Scopus, and Google Scholar, to find the material taken into consideration in this survey. Priority was given to peer-reviewed journal papers that directly address ISAC-V2X architectures, enabling technologies, security, cooperative sensing, and intelligent vehicular applications.

### Main Motivations and Contributions

The principal motivations and contributions are as follows.

- We develop an integrated ISAC-V2X perspective that connects physical-layer design, network architecture, sensing requirements, security, multi-modal intelligence, and application-level constraints within a common 6G vehicular framework [1,3,9].
- We structure the high-mobility networked ISAC-V2X gap (G1) by examining resource management, interference coordination, multi-node cooperation, and mobility-aware operation, and we identify the disconnect between point-to-point theoretical designs and fleet-scale vehicular deployments [5,7,13].
- We synthesize the multi-modal AI-native ISAC-V2X gap (G2) by bringing together RF sensing, radar, LiDAR, camera-based perception, and ML-enabled communication mechanisms while emphasizing the need to jointly consider reliability, security, privacy, and robustness [6,8,14].
- Beyond individual algorithms, we consolidate three deployment-oriented gaps concerning standardization and interoperability (G3), experimental validation and reproducible benchmarking (G4), and scalability, robustness, and cross-domain optimization (G5). Based on these findings, we provide a structured research agenda and a deployment-oriented roadmap toward practical 6G ISAC-V2X systems [2,4].

This paper is organized as follows. The principles, architectures, and taxonomy of ISAC-V2X are introduced in Section 2; vehicular channel models and sensing needs are reviewed in Section 3; and physical-layer approaches are covered in Section 4. Security/privacy and networked ISAC-V2X architectures are covered in Section 5 and Section 6, respectively, while multi-modal and AI/ML-enabled techniques are the subject of Section 7. The primary research gaps, future directions, and the deployment strategy for 2026–2030 are finally compiled in Section 8.

## 2. ISAC-V2X Fundamentals and Taxonomy

Safety-critical 6G V2X objectives that integrate packet transport, scene sensing, and geolocation into a single control loop were described in Section 1. We now map design options onto vehicle mobility patterns, create a common terminology for ISAC, and summarize the main V2X architectural and standards strands. The main meanings of the acronyms used throughout this study are provided in the Abbreviations section.

### 2.1. ISAC Fundamental Concepts

For much of the radio era, radar and packet radio lived in parallel worlds: different frequency plans, front ends, and baseband pipelines. The radar side optimized detection, range-Doppler maps, and track maintenance given fixed waveforms, with ambiguity sidelobes as a primary metric; the communication side modeled fading channels and maximized throughput or outage performance, treating scatterers only through simplified statistical channels [31]. Shared progress in phased arrays, digital receivers, and multiple-input multiple-output (MIMO) transmission later showed deep overlap between the problem sets, motivating today’s 6G agenda to merge communication, sensing, and localization on common air interfaces [32,33].

ISAC denotes a design paradigm in which sensing and communication share radio resources (spectrum, hardware, and waveforms) and are optimized together rather than bolted on as afterthoughts. Compared with “separated” radar–communication deployments that partition bands and duplicate chains, ISAC co-allocates power, time, frequency, spatial, and code resources subject to joint performance constraints on both tasks [32]. Surveys on fundamental limits show that sensing accuracy and communication capacity couple through shared mutual information and Fisher information, which motivates co-designed transmission and estimation rather than treating sensing as a secondary output [34].

ISAC architectures can be positioned along two complementary dimensions. From a functional perspective, communication-centric designs prioritize rate, reliability, or latency while exploiting sensing information for tasks such as CSI refinement and beam management, whereas sensing-centric designs prioritize detection and parameter estimation while embedding communication functions into sensing-oriented transmissions [35,36]. From an integration perspective, radar–communication coexistence (RCC) retains largely separate sensing and communication chains, while dual-function radar–communication (DFRC) and fully integrated ISAC progressively increase hardware, spectrum, and waveform sharing [34,37]. Symbiotic sensing and communications (SSaC) extends this view by describing integration as an evolutionary continuum from loose coexistence toward mutually dependent sensing and communication functions [17].

**Figure 3:**
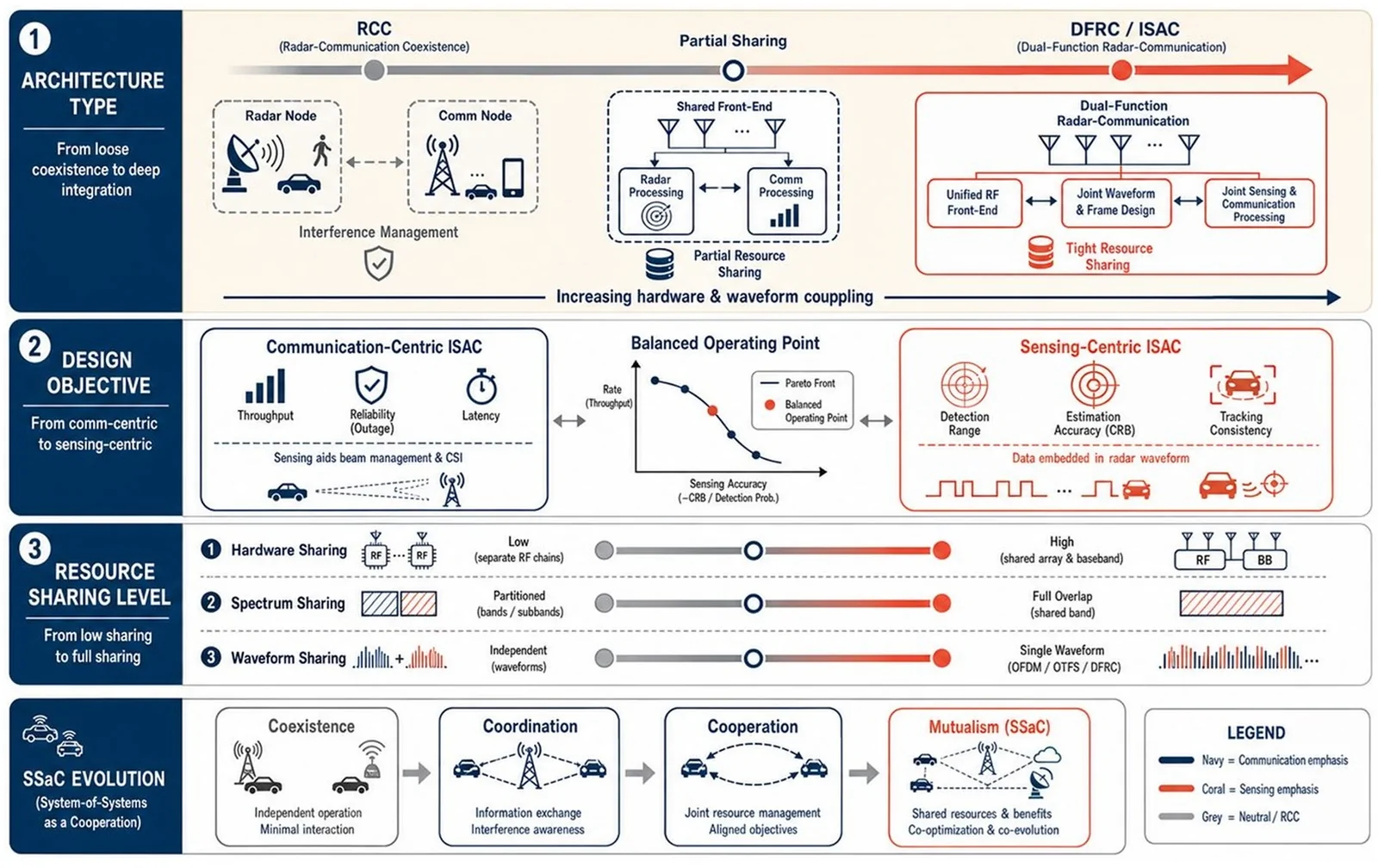
ISAC architecture continuum from RCC to fully integrated ISAC, showing communication- and sensing-centric design objectives and increasing levels of integration.

In large-array DFRC, joint waveform and passive beamforming can shape sensing beampatterns while steering communication beams to limit interference and raise spectral efficiency [38]. Millimeter-wave DFRC for V2X applies directional beams and single-target-multi-beam (STMB) strategies to meet short-range sensing and high-rate links under radar signal-to-interference-plus-noise ratio (SINR) limits, showing how ISAC theory behaves under vehicular mobility and harsh propagation [39]. Figure 3 summarizes the continuum from RCC to DFRC/ISAC.

### 2.2. V2X Architecture and Standards

Flagship 6G vision documents single out V2X for its stringent latency and reliability targets, heterogeneous deployment settings, and need for precise localization and sensing [22,40]. Two legacy pillars dominate today’s deployments: IEEE 802.11p DSRC and C-V2X. DSRC uses contention-based access and short-range broadcasts at low infrastructure cost, but latency and collision statistics become unpredictable in dense traffic [22]. C-V2X employs cellular scheduling and evolved physical layers for better coverage and reliability, first through long-term evolution (LTE)-V2X and later through 5G new radio (NR)-V2X.

The 3rd Generation Partnership Project (3GPP) standardization path toward ISAC has become increasingly explicit across recent releases. NR-V2X evolution through Releases 16 and 17 introduced and enhanced sidelink capabilities relevant to resource allocation, reliability, mobility, and positioning, providing an important foundation for future sensing-assisted V2X operation [22,40]. At the service-requirement level, the Release 19 study item FS_Sensing was conducted during 2022–2023 and produced 3GPP TR 22.837, “Study on Integrated Sensing and Communication,” which addresses ISAC use cases, service requirements, and sensing-related performance indicators [41]. This work was followed by 3GPP TS 22.137, “Integrated Sensing and Communication,” which specifies Stage 1 service requirements [42]. The standardization effort subsequently progressed toward system architecture in Release 20 through the FS_Sensing_ARC study and TR 23.700-14, “Study on Integrated Sensing and Communication; Stage 2,” initiated in 2025 [43]. The radio-interface level was carried out by 3GPP Radio Access Network Working Group 1 (RAN1) during 2025–2026 and resulted in TR 38.765, “Study on Integrated Sensing and Communication (ISAC) for NR” [44]. In parallel, continuing NR positioning enhancements provide enabling mechanisms such as positioning reference signals, measurement procedures, and improved positioning accuracy that are closely related to sensing-assisted localization, beam management, and mobility control. Overall, the 3GPP trajectory is moving from service-level requirements toward system architecture and radio-interface specification, whereas V2X-specific ISAC procedures, particularly sidelink sensing, cooperative sensing, and sensing–result exchange, remain insufficiently standardized.

IEEE 802.11bf provides a complementary wireless local area network (WLAN) standardization track for integrated sensing. IEEE Std 802.11bf-2025 introduces modifications to IEEE 802.11 medium access control and physical-layer service mechanisms to support WLAN sensing across license-exempt frequency bands [45]. Beyond the reuse of communication signals for sensing, the framework supports coordinated sensing operation, including sensing-session establishment and management, capability and parameter negotiation, sensing measurements, and the exchange and reporting of sensing-related information [45,46]. These mechanisms enable applications involving presence and motion detection, ranging, localization, and other environment- or motion-related inference. For heterogeneous V2X deployments, WLAN sensing can therefore complement cellular ISAC at vehicles, roadside infrastructure, intersections, parking areas, and other local environments. However, interoperable procedures for exchanging or jointly exploiting sensing information across IEEE 802.11bf and cellular ISAC remain an open standardization challenge.

**Figure 4:**
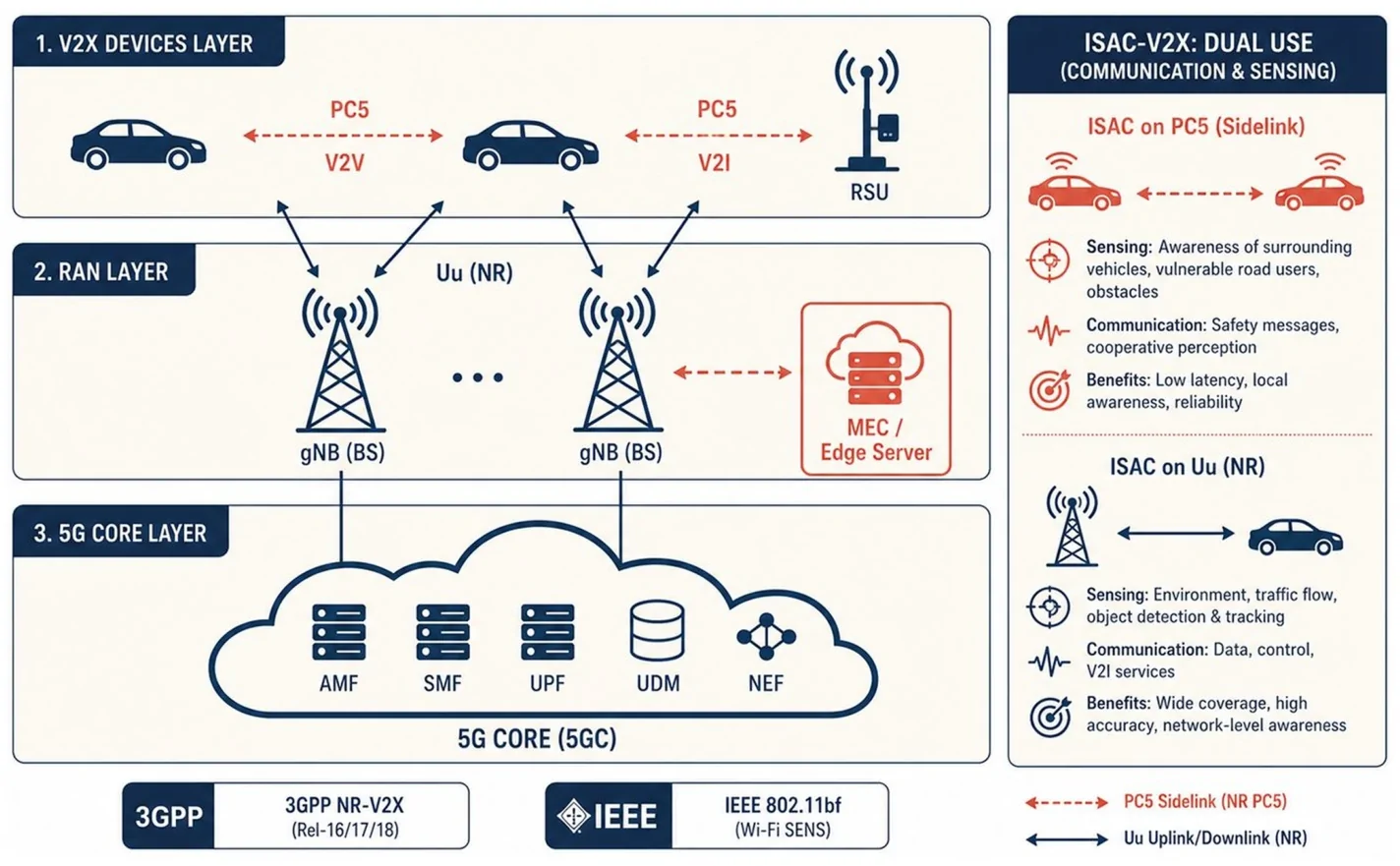
ISAC-V2X network layout with PC5 sidelink for direct V2V/V2I safety traffic, Uu cellular backhaul for wide-area service and network-led sensing, and MEC nodes for low-latency ISAC stream processing.

A structural split runs through these architectures between sidelink (PC5) and uplink/downlink (Uu) interfaces. PC5 sidelink supports direct V2V and V2I links without traversing the core, essential for low-latency safety and cooperative maneuvers [2]. Uu links connect vehicles to base stations (BSs) and the wider network for cloud/edge processing, wide-area service, and traffic management integration [47]. ISAC-V2X can use both: sidelink waveforms may carry safety data and perform relative sensing among neighbors, while Uu links enable network-centric sensing, cooperative perception aggregation, and distribution of situational awareness [39,48]. Multi-access edge computing (MEC; also called mobile edge computing) nodes supply the compute needed to process ISAC streams and close low-latency control loops, making MEC a core element of end-to-end ISAC-V2X [32,47]. Figure 4 depicts the PC5 sidelink, Uu interface, and MEC integration in ISAC-V2X.

Standards for ISAC-V2X will likely crystallize through several converging initiatives rather than one monolithic specification. Hexa-X and related European flagship projects envision 6G with co-designed communication, sensing, and AI-driven computation, listing vehicular services among the primary beneficiaries [40,49]. Depending on the application and regulatory environment, radio access networks that alternate between pure communication, sensing, and intermediate DFRC/RCC modes are surveyed by a joint radar communication architecture [37]. The cellular path is moving from service requirements to architectural and NR interface procedures, while IEEE 802.11bf offers standardized WLAN sensing operation within the IEEE 802.11 ecosystem [41,43,44,45]. The developing 3GPP ISAC specifications, NR positioning evolution, and IEEE 802.11bf WLAN sensing procedures represent complementary standardization tracks.

Practically speaking, ISAC-V2X can facilitate sensing-assisted mobility management, linked and autonomous driving, cooperative perception, and collision avoidance [50]. Additionally, it can enhance network resilience in post-disaster situations, where aerial platforms and V2I/V2V cooperation could provide situational awareness and reestablish emergency connectivity [51]. These applications emphasize the necessity of taking into account latency, mobility, infrastructure availability, communication dependability, and sensing accuracy all at once.

### 2.3. ISAC-V2X Taxonomy

Building on the architectural concepts defined in Section 2.1, this subsection does not introduce a separate ISAC classification. Instead, it organizes representative ISAC-V2X studies along five vehicular design dimensions: architecture, waveform/physical layer (PHY) technique, application focus, mobility regime, and security considerations. This application-oriented taxonomy links the general ISAC integration continuum to the specific requirements of vehicular networks and provides a common structure for the technical sections that follow.

**Figure 5:**
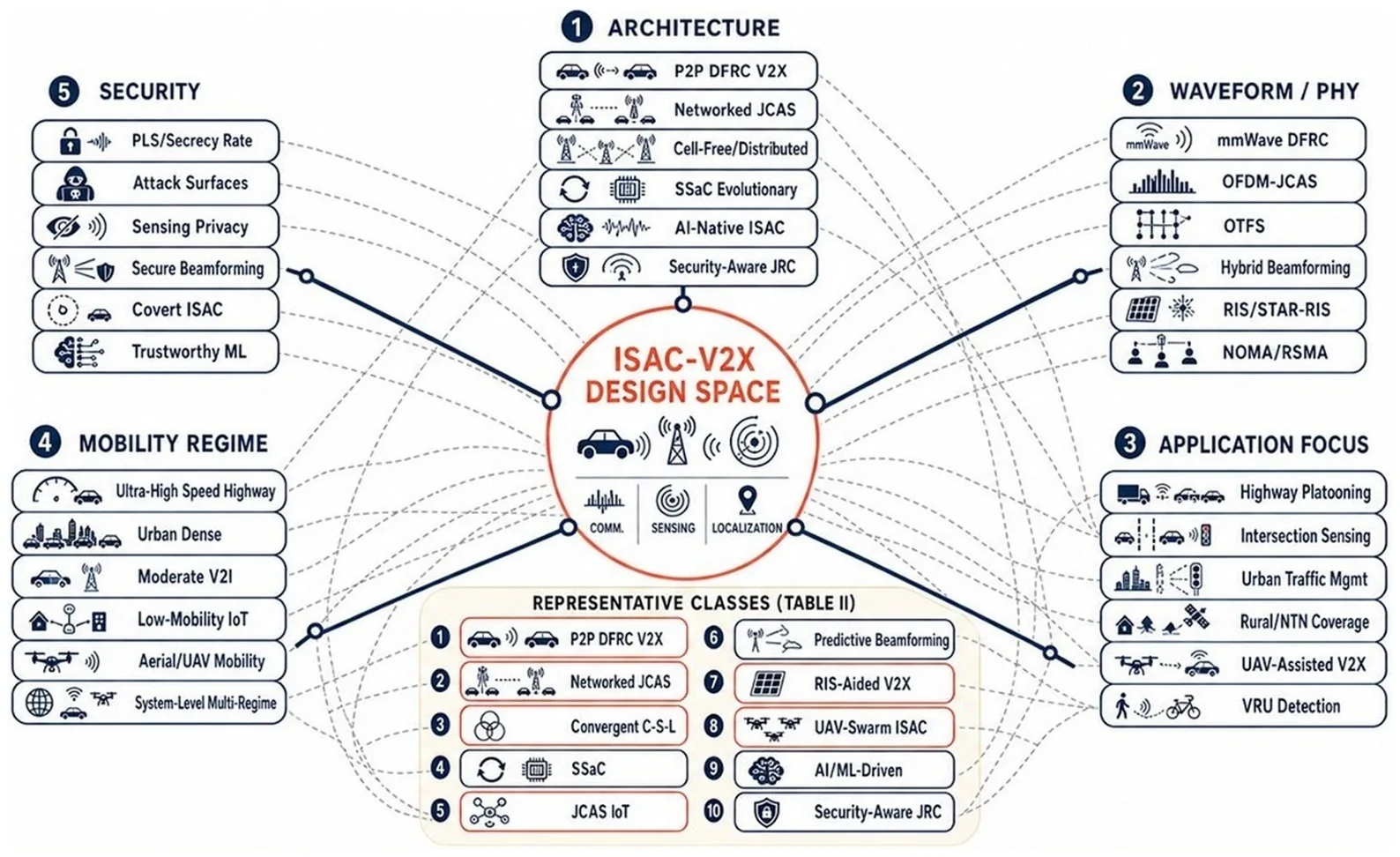
Proposed ISAC-V2X taxonomy organized along five axes: architecture, waveform/PHY technique, application focus, mobility regime, and security considerations.

Representative ISAC-V2X studies span point-to-point DFRC links, networked joint communication and sensing (JCAS), convergent communication–sensing–localization systems, RIS and unmanned aerial vehicle (UAV)-assisted architectures, and AI/ML-enabled designs. Rather than constituting separate taxonomies, these approaches occupy different positions within the five vehicular design dimensions illustrated in Figure 5. In general, point-to-point architectures emphasize waveform and beam design, whereas networked, cooperative, RIS-assisted, and AI-enabled solutions increasingly shift the design burden toward coordination, distributed processing, learning, security, and infrastructure support.

Table 2 provides a deployment-oriented comparison of the principal ISAC-V2X approaches in terms of their advantages, limitations, and suitable vehicular scenarios. The comparison shows that the preferred approach depends strongly on mobility, propagation conditions, infrastructure availability, computational constraints, and the required level of sensing–communication cooperation.

### 2.4. ISAC-V2X Scenario Classification

Deployment scenario strongly shapes mobility, propagation, and performance requirements. We therefore distinguish five recurring ISAC-V2X settings used throughout this survey.

**Highway platooning.** High-speed platoons require low-latency and highly reliable links while maintaining accurate relative sensing. DFRC V2X beams can jointly support payload transmission and estimation of intervehicle spacing and closing speed, while learning-based channel prediction can mitigate rapid Doppler variations and reduce pilot overhead [39,48].

**Intersection cooperative sensing.** Urban intersections require reliable perception under dense clutter, occlusion, and the presence of vulnerable road users. RSUs and BSs can combine communication and sensing information through convergent architectures and OFDM-based JCAS to extend perception beyond LoS and support traffic monitoring [32,35].

**Urban dense traffic.** Dense urban environments introduce severe interference, blockage, and high node density. Dynamic communication/DFRC/RCC operation, coordinated scheduling, and MEC-assisted processing can therefore support low-latency sensing and communication while reducing cross-cell and cross-vehicle interference [37,47].

**Rural and NTN-assisted coverage.** Rural V2X deployments face sparse terrestrial infrastructure and long communication distances. Non-terrestrial networks (NTNs), including LEO satellites and high-altitude platforms, can complement roadside infrastructure by providing wide-area connectivity, sensing support, and backhaul where terrestrial coverage is limited [22,32,40].

**UAV-assisted V2X.** UAVs can jointly provide relaying, sensing, and temporary infrastructure support in areas with limited or disrupted coverage. ISAC-enabled UAVs and swarms can combine trajectory, beam, and waveform design to support target tracking and vehicular connectivity while balancing coverage and energy constraints [62,63].

**Figure 6:**
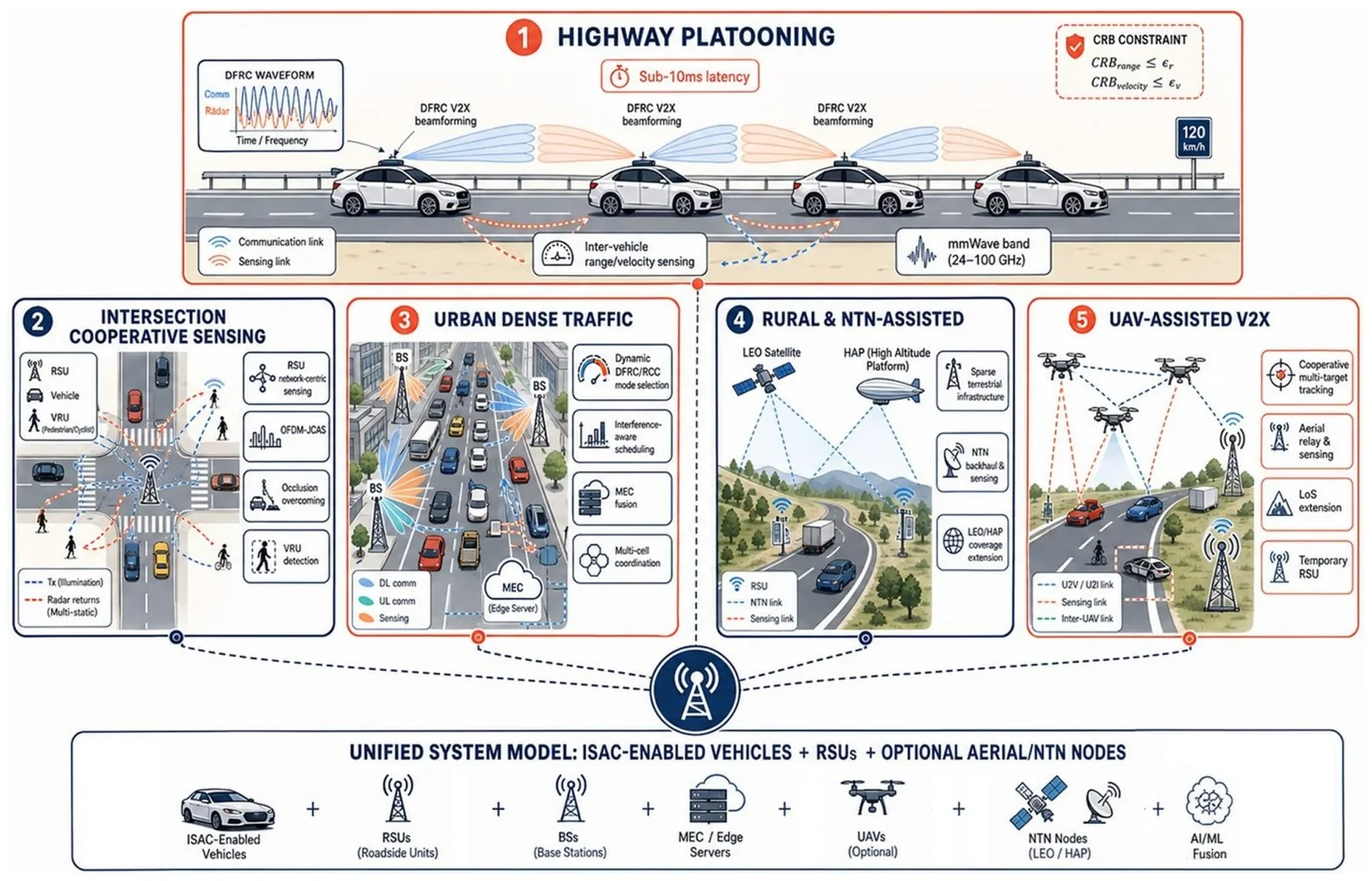
Representative ISAC-V2X deployment scenarios covering highway, intersection, urban, rural/NTN-assisted, and UAV-assisted vehicular environments.

These five scenarios differ in mobility, infrastructure density, propagation conditions, and sensing requirements but share a common ISAC-V2X framework involving vehicles, roadside infrastructure, and optional aerial or non-terrestrial nodes. Figure 6 summarizes the representative deployment settings.

In general, Section 2 outlined the basic ideas, architectures, and taxonomy of ISAC-V2X systems and emphasized how deployment circumstances influence design decisions. Highway platooning, intersection cooperative sensing, dense urban traffic, rural/NTN-assisted coverage, and UAV-assisted V2X are among the scenarios that were taken into consideration. These scenarios demonstrate how mobility, propagation conditions, infrastructure availability, and application requirements have a significant impact on the preferred ISAC architecture and PHY strategy. The vehicular channel models and sensing needs covered in Section 3, which describe how multi-path, motion, and street layout bound waveforms and feasible key performance indicators (KPIs) in each circumstance, are based on these architectural and scenario-level findings. The results mainly drive G1 and G5 because high mobility and heterogeneous deployment conditions necessitate scalable architectures, cross-domain optimization, and performance frameworks that hold up well in a variety of vehicle settings.

## 3. Vehicular ISAC Channel Models and Sensing Requirements

This section reviews vehicular channel characteristics relevant to ISAC, ISAC-specific channel modeling approaches, sensing performance metrics, and channel estimation techniques, with emphasis on measurement-based and stochastic models.

### 3.1. Vehicular Channel Characteristics

**Figure 7:**
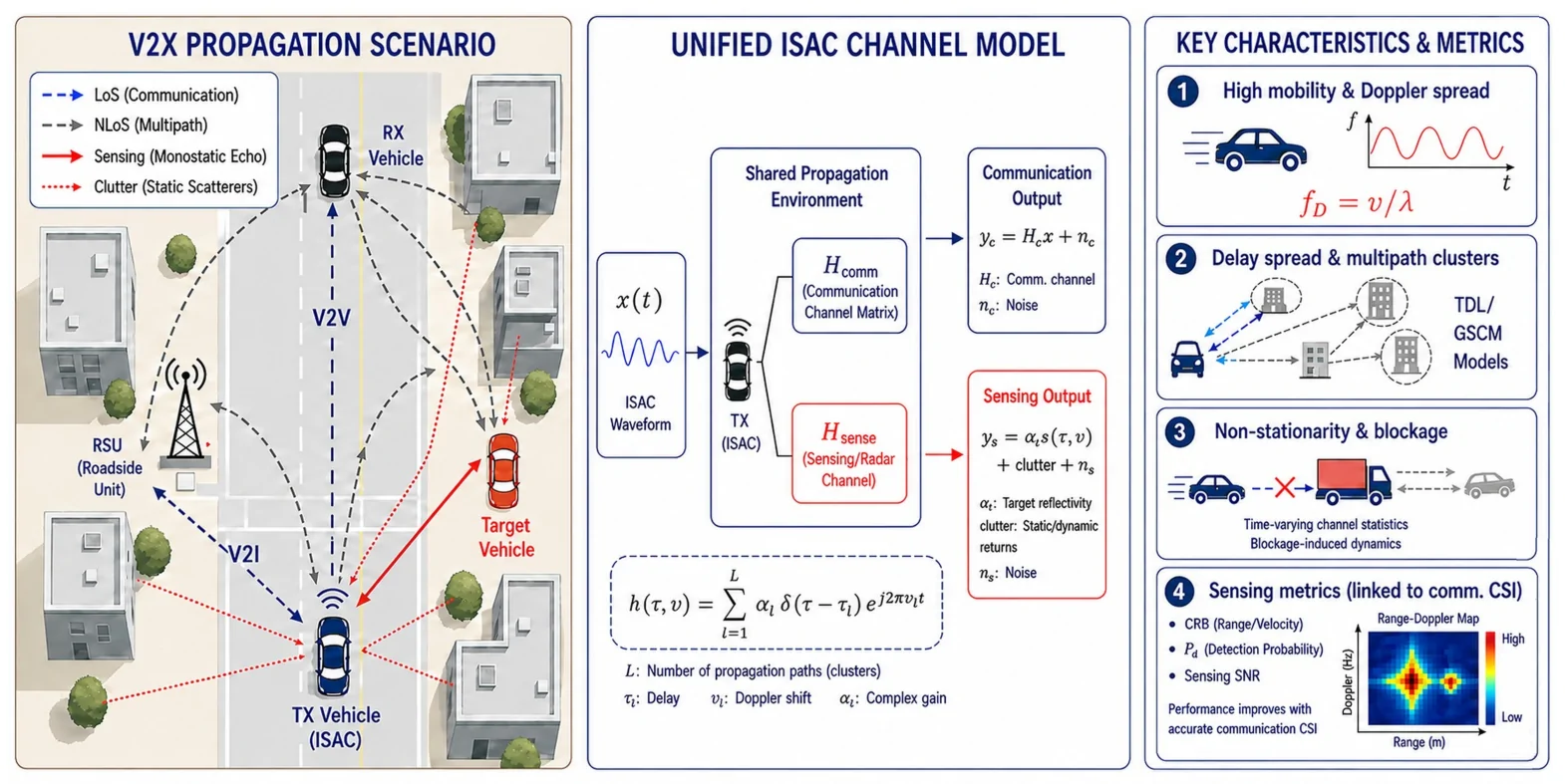
Vehicular ISAC channel model showing shared V2X propagation, unified communication and sensing channel representations, mobility and multi-path effects, and representative sensing metrics.

Communication quality and sensing accuracy are significantly impacted by the unique propagation behavior of the vehicular channels at millimeter-wave (mmWave) and sub-terahertz bands. Free-space path loss increases dramatically with distance at 28 GHz and 60 GHz, and obstructions caused by cars, structures, and vegetation result in significant shadowing and non-stationary fading [64,65]. In 3GPP and geometry-based stochastic channel model (GBSM) frameworks, path loss models for V2V, V2I, and V2N links have been standardized and expanded; these models usually distinguish between LoS and non-LoS (NLoS) conditions and parameterize delay spread, angular spread, and cross-polarization ratio as functions of scenario and antenna configuration [27,66]. Figure 7 depicts the key modeling ingredients that differentiate vehicular ISAC channels from communication-only abstractions (one-way vs. two-way sensing paths, targets/clutter, and shared clusters).

Severe Doppler spread is introduced by high mobility, for example, a car traveling at 120 km/h experiences a maximum Doppler shift of several kilohertz at 28 GHz, which reduces channel coherence time and eliminates subcarrier orthogonality in OFDM unless countermeasures like larger subcarrier spacing or Doppler-resistant waveforms are used [67,68]. Similar difficulties are faced by automotive joint communication and radar systems (JCRSs) operating at 77 GHz; hardware and waveform selections (such as phase-coded continuous-wave code-division multiple access (CDMA)) must take into consideration both radar range-velocity resolution and communication equalization under the same Doppler spectrum [69]. At 60 GHz and beyond, oxygen and rain attenuation add to free-space loss, and antenna gains and beamwidths become central to maintaining both link budget and angular resolution for sensing; hybrid beamforming designs for mmWave massive MIMO in Internet of Vehicles (IoV) with dual-functional radar–communication illustrate how array configuration and beam management interact with these channel characteristics [65].

### 3.2. ISAC-Specific Channel Models

Unlike conventional communication-only or radar-only models, ISAC channel models must represent both the one-way link from transmitter to communication receiver and the two-way (round-trip) path from the sensing transmitter to the target and back. Survey work on ISAC channel modeling emphasizes that radar sensing channels depend on target radar cross-section (RCS) and clutter RCS and that deterministic (e.g., ray-tracing, physical optics) and statistical (e.g., Swerling, log-normal) RCS models are needed in addition to propagation path loss and multi-path structure [27]. A key structural distinction is that communication channels are usually modeled as one-way, with stochastic multi-path components (MPCs) between fixed or mobile nodes, whereas sensing channels involve the same transmit/receive location (monostatic) or distributed nodes (bistatic/multi-static) and incorporate target and clutter scattering explicitly.

Shared clusters between sensing and communication channels have been observed in measurement campaigns. Because the same hardware and environment are shared, a subset of scatterers can contribute to both the communication MPCs and the sensing echoes; independently generating the two channel types under a common geometry can therefore underestimate their correlation. Liu et al. proposed a shared cluster-based stochastic channel model in which communication and sensing channels are jointly generated by superimposing shared clusters (from common scatterers) and non-shared clusters, with parameters extracted from 28 GHz indoor ISAC measurements and a K-power-means-based joint clustering algorithm. The resulting “sharing degree” (SD) metric quantifies how much the two channels overlap in the angular-delay space and is useful for evaluating ISAC system design and deployment [70].

For dynamic vehicular ISAC, time-varying behavior is critical. Zhang et al. reported channel measurements at 28 GHz in dynamic vehicular scenarios and proposed a tapped-delay-line model that distinguishes sensing multi-path components (S-MPCs) from clutter multi-path components (C-MPCs) [64]. S-MPCs correspond to reflections from moving or persistent targets of interest and exhibit birth–death processes, lifetime statistics, and time-varying power and delay within their lifetimes; C-MPCs are modeled as a random clutter floor with clustering, power decay, and small-scale fading. The model is parameterized from measurements in front, left, and right sensing directions and supports simulation of dynamic evolution over time, which is essential for platooning, intersection, and urban ISAC-V2X scenarios. GBSM and beam-domain channel models (BDCMs) for 6G mmWave massive MIMO ISAC extend this idea by incorporating three-dimensional geometry, non-stationarity, and the correlation between sensing and communication channel statistics (e.g., forward and backward scattering) for system-level simulation [66,71].

### 3.3. Sensing Performance Metrics

Sensing performance in ISAC systems is commonly quantified by estimation accuracy bounds and detection statistics. The CRB provides a lower bound on the variance of unbiased estimators for parameters such as range, velocity, and angle; it is widely used to constrain or optimize waveform and beamforming design so that sensing meets a minimum accuracy while communication rate or power is maximized [72,73]. For joint angle and delay estimation, the CRB can be derived from the Fisher information matrix (FIM) of the received signal model, and transmit beamforming can be optimized to minimize the CRB subject to communication rate and power constraints. In RIS-aided and near-field ISAC systems, CRB analysis has been extended to multi-user, multi-target settings and to semi-passive RIS configurations where active elements support sensing and passive elements support communication [74].

Detection probability and false alarm rate are standard radar metrics. In ISAC-capable V2X sidelink, resource allocation is often random or sparse in time and frequency, so the received OFDM grid is unstructured, and conventional two-dimensional fast fourier transform (FFT)-based processing may suffer from high sidelobes and missed or false detections. High-resolution off-grid target detection algorithms, such as those based on Newtonized orthogonal matching pursuit (NOMP), have been proposed to detect weak targets masked by sidelobes and to operate regardless of the OFDM grid structure [75]. Range-velocity resolution is determined by waveform bandwidth and coherent processing interval; delay-Doppler domain designs (e.g., block-shaped sensing reference signals) allow joint range and velocity estimation with controlled complexity and resilience to noise, which is relevant for beyond 5G (B5G) and 6G ISAC under resource constraints [76].

Information theory and estimate theory have been used to examine fundamental trade-offs between sensing and communication in monostatic ISAC. By connecting waveform and resource decisions to the Pareto frontier of sensing accuracy vs. communication rate [68], Keskin et al. offer a comprehensive analysis of these trade-offs approaching 6G. The probability of detection and the amount of time available for data transmission are directly impacted by the selection of the detection threshold and number of sensing repetitions in resource-constrained or latency-critical V2X settings. Analytical models that link the above parameters to block length and antenna imperfections support low-complexity optimization and help bridge the gap between theoretical CRB-based design and deployable ISAC-V2X systems [77]. These metrics and trade-offs directly inform the physical-layer and resource management techniques covered in Section 4.

### 3.4. Channel Estimation Techniques

Accurate CSI is needed for both communication demodulation and sensing parameter extraction. Pilot design for ISAC waveforms must allocate time-frequency resources for channel estimation while preserving sufficient degrees of freedom for sensing (e.g., range-Doppler resolution) and minimizing overhead [27]. In carrier aggregation (CA)-based ISAC, different pilot structures can be used for sensing across aggregated bands, and compressed sensing (CS) has been applied to improve sensing performance from fragmented or sparse observations [78]. Joint angle-delay sparse structured compressed sensing for ISAC channel estimation exploits the overlap of sensing and communication channel support in the angle and delay domains and designs expectation-maximization (EM)-based CS algorithms tailored to the ISAC channel sparse structure [79].

Deep learning (DL)-assisted channel estimation has been proposed to handle the coupling and interference between sensing and communication streams in RIS-assisted ISAC systems. A three-stage approach decouples the estimation of direct sensing/communication channels, reflected communication channel, and reflected sensing channel using convolutional neural networks (CNNs), improving estimation quality compared with least-squares baselines under various signal-to-noise ratio (SNR) and system parameters [80]. Such data-driven methods can reduce pilot overhead and computational cost at the expense of training data and robustness to out-of-distribution conditions; the trade-off between overhead and accuracy is particularly relevant in high-mobility vehicular ISAC where coherence time is short [81].

UAV-enabled ISAC tracking designs often minimize a weighted sum of predicted posterior CRB (PCRB) for position and velocity estimation by optimizing the UAV trajectory and, where applicable, bandwidth allocation; the PCRB captures the achievable estimation accuracy after accounting for motion and measurement models [81]. Overall, channel estimation techniques for ISAC-V2X must address time-varying, possibly shared communication and sensing channels, clutter, and strict latency constraints, motivating continued work on pilot design, CS, and learning-based methods.

### 3.5. Channel Models Comparison

Table 3 summarizes representative channel models and their main characteristics relevant to vehicular ISAC. The categories include carrier frequency, scenario, mobility, model type (deterministic vs. stochastic, one-way vs. two-way), and key parameters (e.g., RCS, shared clusters, S-MPCs/C-MPCs, CRB).

Section 3 has summarized vehicular channel characteristics, ISAC-specific channel models (including shared clusters and dynamic S-MPC/C-MPC models), sensing performance metrics (CRB, detection, range-velocity resolution, and sensing–communication trade-offs), and channel estimation techniques (pilots, compressed sensing, and DL). Section 4 addresses physical-layer techniques for ISAC-V2X, waveform design, beamforming, and RIS-aided solutions that build on these channel and sensing foundations to achieve joint communication and sensing performance in vehicular networks. These findings primarily motivate G1 and G5 because high-mobility channel dynamics, sensing–communication coupling, and heterogeneous propagation conditions require unified performance metrics and scalable optimization methods that remain reliable across rapidly varying vehicular environments.

## 4. Physical-Layer ISAC-V2X Techniques

Building on the channel models and sensing requirements outlined in Section 3, this section reviews physical-layer techniques that enable integrated sensing and communication in V2X and vehicular networks. The techniques discussed here form the signal-processing and optimization foundation for dual-functional systems that share spectrum, hardware, or both between radar-like sensing and communication; they are directly relevant to 5G NR, C-V2X, and future 6G V2X standards where ISAC is expected to support applications such as autonomous driving, platooning, and roadside sensing. We cover waveform design, beamforming optimization, RIS/STAR-RIS assistance, NOMA/RSMA-based ISAC, power and resource allocation at the physical layer, and the fundamental sensing–communication trade-off. The emphasis is on techniques that are directly applicable to high-mobility vehicular scenarios and that align with 5G, B5G, and 6G radio interfaces.

### 4.1. ISAC Waveform Design for V2X (OFDM, OTFS, DFRC)

Waveform design is central to DFRC and ISAC systems, as the same transmitted signal must support both reliable data demodulation and accurate sensing (range, velocity, angle). Existing work can be grouped into communication-centric, sensing-centric, and joint waveform optimization [85,86]. In V2X, waveforms must cope with high Doppler, time-varying channels, and strict latency while preserving compatibility with cellular or sidelink standards.

**Figure 8:**
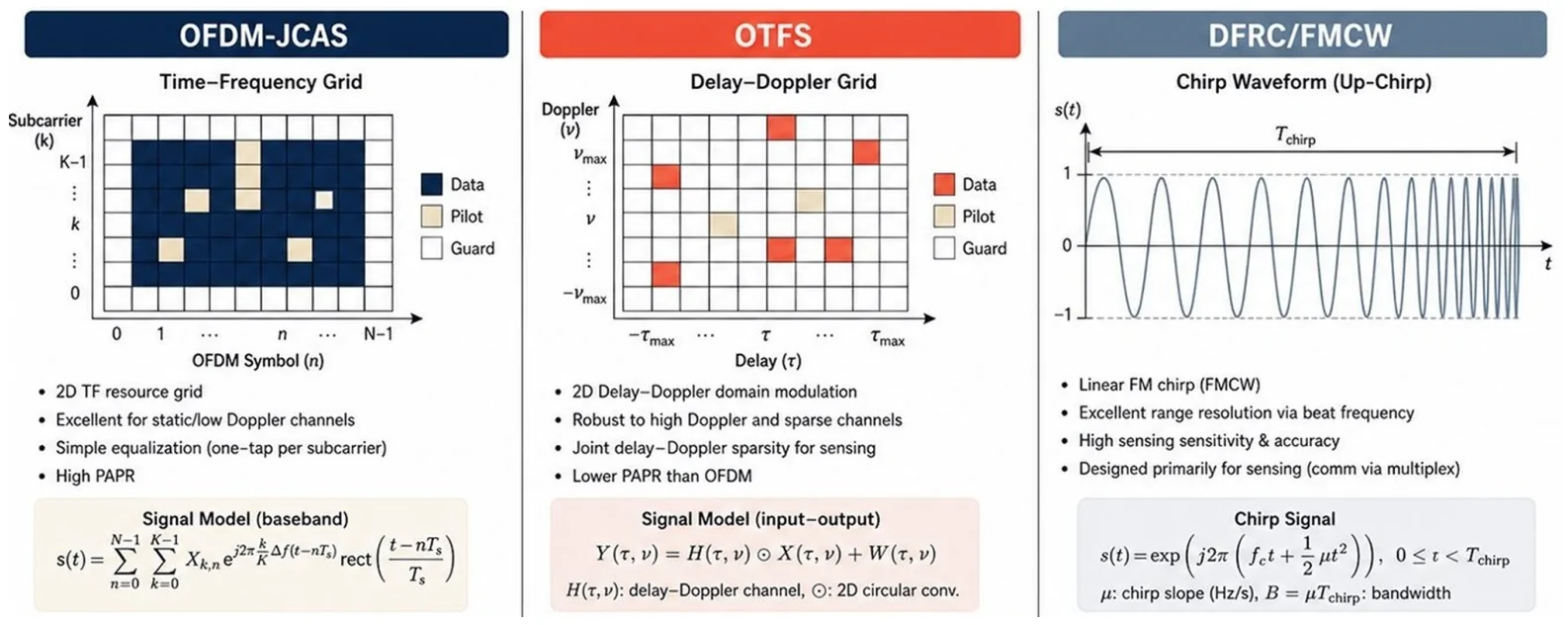
Comparison of representative ISAC-V2X waveform designs, including OFDM, OTFS, and radar-native approaches, with their main sensing–communication and mobility trade-offs.

As a motivating comparison for high-mobility regimes, Figure 8 contrasts OFDM and OTFS signal representations commonly used in ISAC-V2X studies.

**OFDM-based ISAC.** OFDM is the dominant waveform in 4G/5G and is widely used as a communication-centric base for ISAC. Its flexibility in time and frequency allows radar target detection via matched filtering or symbol-level processing. Modulation-symbol-based processing (using transmitted and received modulation symbols rather than raw baseband signals) reduces complexity for range and Doppler estimation in OFDM-ISAC. The number of subcarriers $N_{c}$, number of symbols $N_{s}$, subcarrier spacing Δf, and power allocation across subcarriers are key parameters: they affect range resolution (via bandwidth $N_{c}\Delta f$), velocity resolution (via coherent processing over $N_{s}$ symbols), and communication rate; joint optimization of these parameters under mutual information and rate constraints has been studied in the literature [85]. In the standard baseband OFDM formulation, the signal is a sum over subcarriers and symbols with data symbols a(m,n), carrier $f_{c}$, and symbol duration $T_{s}$. At the ISAC node, the noiseless echo from a point target with delay τ and Doppler $f_{D}$ can be expressed in the discrete time-frequency domain; block-wise FFT and correlation then yield range and velocity estimates [86]. Power and subcarrier allocation directly affect both communication rate and sensing accuracy. Joint optimization of subcarrier power, number of subcarriers/symbols, and subcarrier spacing under mutual information (MI) and rate constraints has been studied to balance performance. Peak-to-average power ratio (PAPR) reduction and sidelobe control (e.g., via complementary subcarriers or phase coding) are important for practical OFDM-ISAC in vehicular environments [85]. Space-time-frequency resources of OFDM signals can be cooperatively created in cooperative multi-BS ISAC to enhance sensing fusion and coordinate interference [87]. Doppler-resilient alternatives like OTFS are motivated by OFDM’s intercarrier interference (ICI) and sensitivity to Doppler in high-mobility regimes, notwithstanding its benefits.

**OTFS for high-mobility V2X.** OTFS modulation places symbols in the delay-Doppler (DD) domain and spreads them over the full time-frequency grid, offering robustness in doubly dispersive channels [37,67]. The DD-domain symbols $X_{\mathrm{dd}}\left[k,l\right]$ are related to the time-frequency symbols via the inverse symplectic finite Fourier transform (ISFFT). In vehicular networks, the RSU can use OTFS-ISAC signals to estimate delays, Dopplers, and angles of vehicles, build a dynamic topology, and then predict channel parameters for the next time instant; this supports pre-equalization for downlink and pilot-free or reduced-pilot uplink channel estimation. Because the DD representation aligns with the channel’s delay and Doppler spreads, OTFS enables efficient equalization and improved reliability in high-mobility vehicular links. For ISAC, the same OTFS waveform can be used for both communication and sensing: the RSU (or vehicle) transmits OTFS-ISAC signals, estimates target parameters from echoes (e.g., delay, Doppler, angle), and uses the estimated topology to design beamformers and, in the uplink, to predict delays/Dopplers and reduce pilot overhead [67]. Range-velocity estimation in OTFS-ISAC can be performed in the DD domain via 2D correlation or matched filtering; the achievable accuracy is comparable to OFDM in many settings, with potential benefits in highly dynamic channels [37,76]. Recent OTFS-based ISAC studies further extend delay-Doppler processing to cooperative and cell-free architectures, demonstrating its suitability for high-mobility sensing and communication scenarios [52]. A drawback of OTFS is higher receiver complexity than conventional OFDM; trade-offs between complexity, mobility, and standard compatibility remain active topics. Hardware impairments such as phase noise and frequency offset affect OFDM and OTFS differently and can influence the choice of waveform in practical vehicular ISAC deployments [37].

**DFRC and joint waveform design.** By deliberately improving sensing and communication goals within a shared signal structure, DFRC and collaborative waveform design go beyond just reusing communication waveforms. Current concepts include completely integrated waveform and precoder optimization, orthogonal or cognitive spectrum sharing, and expansions to adaptive, multi-node, and RIS-assisted operation [7,35]. Although these methods offer more control over sensing–communication trade-offs, they typically result in non-convex, high-dimensional optimization problems and call for more specific CSI or target information. In vehicular ISAC, the choice of waveform also depends on the deployment: RSU-to-vehicle links may use OFDM or OTFS depending on mobility and standard compliance; V2V sidelink often reuses the same waveform for consistency with C-V2X or NR sidelink, with sensing performed on the same reference or data symbols when permitted by the frame structure [3,7].

For ISAC, new waveform options like index modulation (IM) and orthogonal chirp division multiplexing (OCDM) have also been investigated; IM preserves high correlation for sensing while embedding information in resource indices (such as antenna or subcarrier activation) [85]. Taken together, the waveform literature reveals three recurring design directions. Communication-derived waveforms, particularly OFDM, offer the strongest compatibility with existing cellular V2X systems but become increasingly sensitive to Doppler and intercarrier interference at high mobility. Delay-Doppler-domain approaches such as OTFS improve robustness in rapidly time-varying channels, although this benefit comes with increased receiver processing complexity. Jointly optimized DFRC waveforms provide greater flexibility in controlling sensing–communication trade-offs but typically require more detailed CSI or target information and more complex optimization. The practical waveform choice is therefore governed by a trade-off among standard compatibility, mobility robustness, sensing performance, and implementation complexity rather than by a universally superior design. Representative ISAC waveform and beamforming approaches for V2X are consolidated in Table 4.

### 4.2. Beamforming Optimization (Hybrid, Digital, Analog)

**Figure 9:**
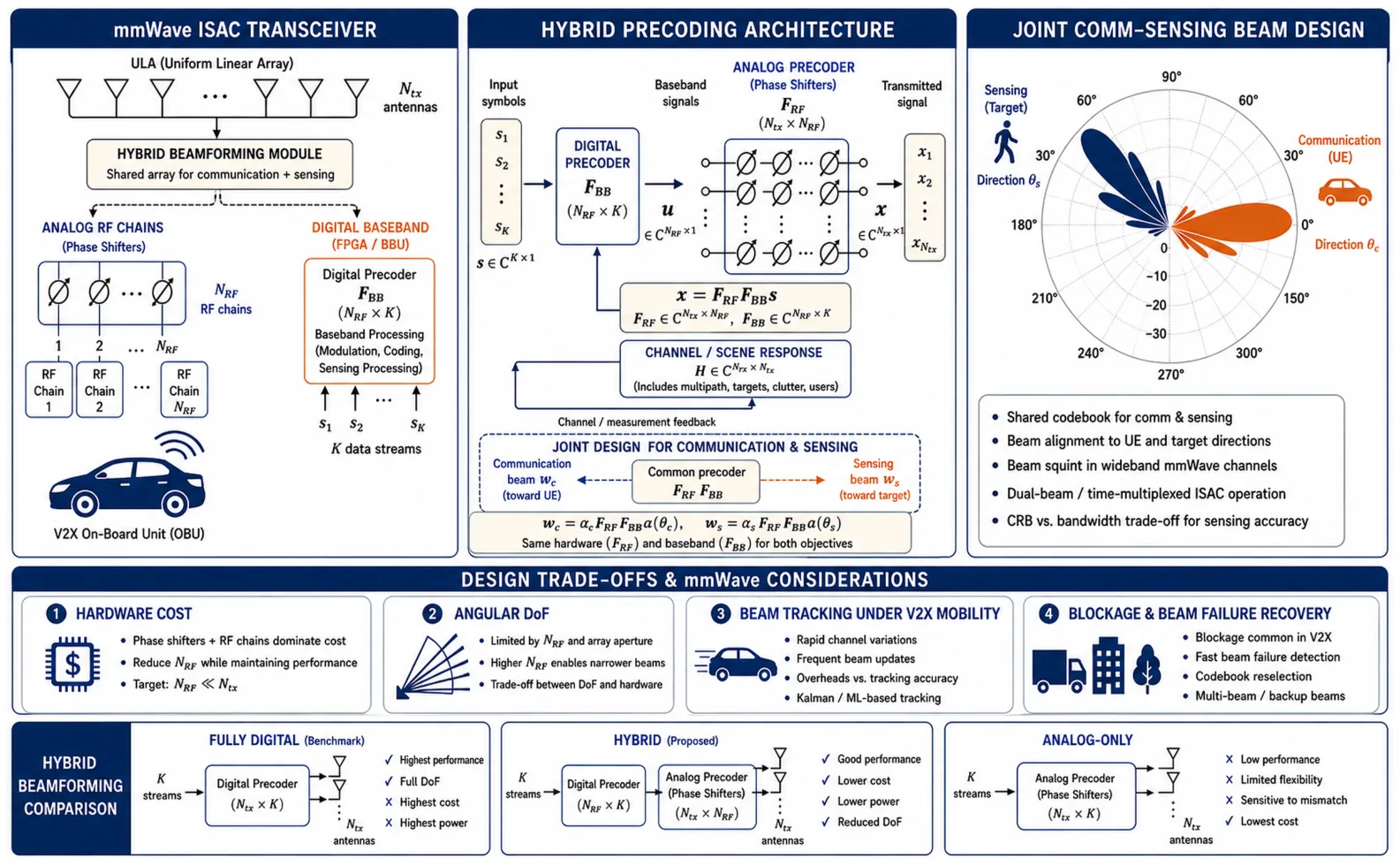
Hybrid beamforming architecture for mmWave ISAC-V2X, showing shared analog–digital precoding for joint communication and sensing beam design.

Beamforming determines the spatial directivity of the transmitted (and optionally received) ISAC signal, affecting both communication link quality and sensing accuracy. In mmWave V2X, hybrid analog–digital (HAD) structures are preferred to limit RF chain count while retaining multi-user and multi-target capabilities. Figure 9 illustrates a representative hybrid beamforming architecture for mmWave ISAC-V2X, where directional beams support both multi-user communication and target illumination/sensing.

**Hybrid analog–digital beamforming for V2X DFRC.** Liu et al. consider mmWave DFRC in V2X with an STMB radar scheme: multiple beams are allocated to one target to improve range/velocity and velocity-direction estimation [39]. The transmit uniform linear array (ULA) uses HAD beamforming (fewer RF chains than antennas); the receive ULA is assumed fully digital. With echo model $y_{\mathrm{echo}}=\sum_{i=1}^{K}\alpha_{i}b\left(\theta_{i}\right)a^{T}\left(\theta_{i}\right)x_{o}+n_{\mathrm{echo}}$, where $a(\theta_{i})$, $b(\theta_{i})$ are steering vectors, and $\alpha_{i}$ the reflection coefficient, the problem is formulated as maximizing the communication rate subject to per-target radar SINR constraints. A radar beam cancellation algorithm adapts the number of beams per target to satisfy the SINR under different power levels. The same work derives the HAD beamformer structure and shows gains over a zero-forcing-only baseline in both spectral efficiency and minimum radar SINR. Cong et al. study vehicular behavior-aware (VBA) ISAC beamforming with an HAD architecture: the transmitted signal is $x=F_{\mathrm{RF}}F_{\mathrm{BB}}s$, with $F_{\mathrm{RF}}$ and $F_{\mathrm{BB}}$ the analog and digital beamformers, under a normalized power constraint $∥F_{\mathrm{RF}}F_{\mathrm{BB}}{∥}_{F}^{2}=N_{s}$ [91]. The joint design balances communication and sensing via a trade-off factor and is solved using semidefinite relaxation (SDR). Yu et al. focus on hybrid beamforming in mmWave massive MIMO for the IoV with DFRC, aligning beams with both communication users and radar targets under hardware constraints [65]. These works show that HAD beamforming is a practical choice for vehicle-mounted or RSU-mounted ISAC with limited RF chains.

**Digital and analog beamforming.** Fully digital beamforming offers the finest control (independent amplitude and phase per antenna) and is often used as a performance upper bound or in small arrays. It requires one RF chain per antenna, which becomes costly and power-consuming at mmWave with large arrays; hence hybrid or analog-only solutions are preferred for massive MIMO ISAC in vehicles and RSUs [95]. Analog beamforming uses a single (or few) RF chain(s) and phase shifters to steer one or a small number of beams; it is low-cost and suitable for single-stream or radar-only sub-systems but offers limited flexibility for multi-user or ISAC optimization. Hybrid beamforming strikes a balance: the analog stage provides coarse spatial filtering, and the digital stage performs multi-stream precoding so that the number of RF chains can be much smaller than the number of antennas while still supporting multiple users and targets [96]. In ISAC, digital precoding can be designed to shape the radar beampattern while satisfying communication SINR or rate constraints [86,92]. Analog beamforming uses phase shifters (and possibly attenuators) with a single or few RF chains; it is low-cost and energy-efficient but typically supports one or a small number of beams. Hybrid beamforming combines an analog stage (e.g., phase-only) with a digital stage to approach full-digital performance with reduced RF chains. For ISAC, the beamformers are often optimized jointly with waveform or resource allocation; the resulting problems are non-convex and are tackled by alternating optimization, SDR, or learning-based methods [92,93].

**Joint beamforming with sensing and communication objectives.** Zhao et al. address joint beamforming for multi-target detection and multi-user communication in ISAC: the transmit covariance (or precoder) is optimized to maximize a weighted sum of communication and sensing metrics (e.g., sum rate and detection SNR) under power constraints [92]. The radar beampattern can be shaped to place nulls toward communication users (to reduce interference) and mainlobes toward targets; conversely, communication beams are steered toward users while satisfying a minimum radar SINR or beampattern mask. Such joint design leads to non-convex quadratically constrained problems that are typically solved by SDR, successive convex approximation (SCA), or block coordinate descent (BCD) [86]. Barneto et al. design and optimize beamformers for joint communication and full-duplex sensing at mm-waves, including the impact of self-interference [94]. Rihan et al. consider robust RIS-assisted MIMO communication–radar coexistence with joint beamforming and waveform design to guarantee radar and communication performance under channel uncertainty [84]. In RIS-enabled mmWave ISAC, joint hybrid beamforming at the BS and phase shifts at the RIS can be optimized to maximize communication sum rate or sensing SNR, or to balance both [93]. Predictive or learning-based beamforming has been proposed to reduce training overhead and adapt to mobility: for example, a low-rank adaptation (LoRA) transformer-based long short-term memory (LSTM) model integrated with a graph neural network (GNN) can predict ISAC beamforming in ultra-dense device-to-device (D2D) mmWave scenarios, and deep unfolding or reinforcement learning can aid transceiver design when channel or target state changes rapidly [97,98]. Table 4 compares representative beamforming strategies (and waveform designs) and their applicability to ISAC-V2X. The choice among analog, digital, and hybrid beamforming depends on the number of antennas, cost and power constraints, and the need for multi-user or multi-target support in a given V2X or RSU deployment; hybrid structures are often the preferred compromise for mmWave ISAC.

### 4.3. RIS/STAR-RIS-Assisted ISAC-V2X

**Figure 10:**
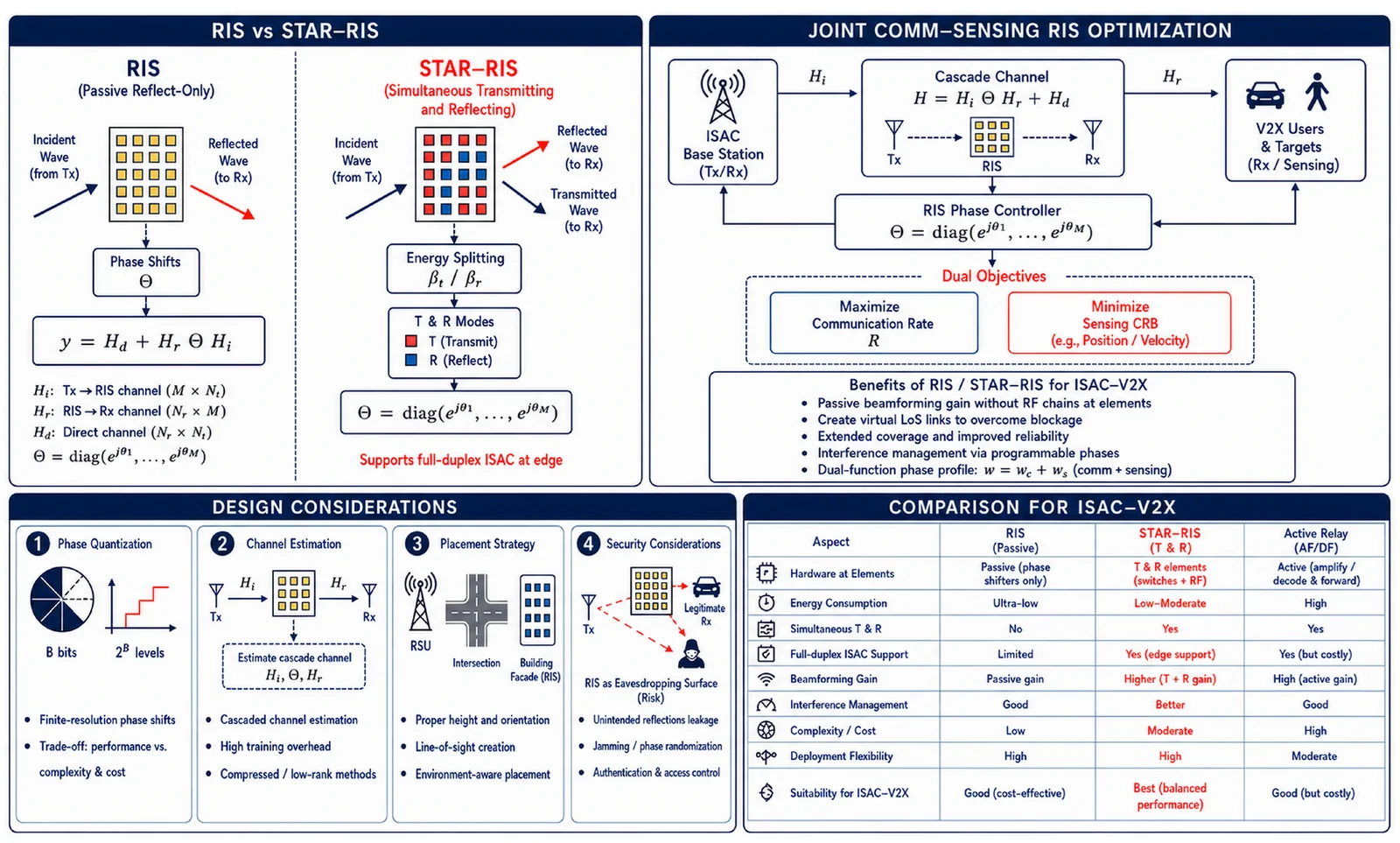
RIS/STAR-RIS-assisted ISAC-V2X architecture illustrating reconfigurable propagation control for joint communication and sensing in NLoS scenarios.

RISs and STAR-RISs add controllable reflection (and optionally refraction) paths, extending coverage and improving both communication and sensing in NLoS or blocked scenarios. RIS-assisted ISAC has been standardized and discussed in 3GPP and the European Telecommunications Standards Institute (ETSI) for 6G use cases, including object tracking and environmental mapping; the joint optimization of BS beamforming and RIS phase shifts (and, for active or multi-functional RIS, amplitudes) is a central design problem that couples communication and sensing performance [28]. Figure 10 visualizes RIS and STAR-RIS-assisted ISAC-V2X links, emphasizing how controllable reflected/refracted paths can jointly improve coverage for both sensing and communication.

**Passive RIS for ISAC.** RISs consist of passive (or near-passive) elements whose phase shifts (and sometimes amplitudes) are configurable. In ISAC, they can create virtual LoS to targets and users, improve angular resolution, and reduce the impact of blockages [86]. Wang et al. jointly design the communication waveform and discrete RIS phase shifts under a CRB constraint for sensing, improving estimation accuracy while maintaining communication performance [10]. Ismail et al. propose an enhanced artificial ecosystem optimizer (EAEO) for RIS-assisted ISAC beamforming, maximizing SNR at communication users while satisfying sensing constraints; the formulation includes two RISs and a dual-function BS [99]. Wei et al. study wideband RIS-aided holographic DFRC with a reconfigurable holographic surface (RHS) at the transmitter and a passive RIS in the channel; joint design of digital, holographic, and passive beamformers maximizes worst-case radar SINR subject to communication SINR constraints, with alternating maximization and handling of constant-modulus and difference-of-convex constraints [88]. Rihan et al. address robust RIS-assisted MIMO coexistence with joint beamforming and waveform design under imperfect CSI [84].

**Active RIS and STAR-RIS.** Active RIS elements can amplify the reflected signal, mitigating the double path loss of passive RIS [100]. Salem et al. study active RIS-assisted MISO ISAC for secure operation, optimizing beamforming and RIS coefficients to improve secrecy and sensing. Multi-functional RIS (MF-RIS) architectures that integrate reflection, refraction, and amplification in a single surface have been proposed for full-space coverage and better performance than conventional passive or single-sided STAR-RIS in ISAC [101]. STAR-RISs split incident power into reflected and refracted components, serving users and targets on both sides of the surface. Zhu et al. consider STAR-RIS-aided multiple-input single-output (MISO) ISAC secure communications with energy-splitting and time-switching protocols; long-term average secrecy rate is maximized subject to echo SNR and rate constraints, and the problem is solved using deep deterministic policy gradient (DDPG) and soft actor-critic (SAC) [56]. Zhao et al. investigate Pareto optimization for MF-RIS-assisted ISAC with multiple users and targets; a multi-objective formulation maximizes communication SINR and sensing SNR, and a modified Chebyshev scalarization with alternating optimization (AO) is used to obtain the Pareto boundary [101]. These works show that RIS and STAR-RIS can significantly improve ISAC performance in vehicular and indoor scenarios when phase (and, for active/MF-RIS, amplitude) are jointly optimized with transmit beamforming. For V2X specifically, RIS can be deployed at road margins or on infrastructure to create virtual LoS to vehicles in blockage or at cell edges; the same phase configuration can enhance both the communication link to the vehicle and the echo path for sensing the vehicle or nearby targets [86]. Joint waveform and discrete phase-shift design under CRB constraints, as in [10], is representative of the class of problems that couple RIS optimization with ISAC performance metrics; the discrete phase constraint is typically relaxed or handled by quantization in iterative algorithms.

### 4.4. NOMA/RSMA for ISAC-V2X

Non-orthogonal multiple access (NOMA) and RSMA allow multiple users to share the same time-frequency resources via superposition and successive interference cancellation (SIC) or via common and private streams [102,103]. Both can be combined with ISAC to improve spectral efficiency and to manage interference between communication and sensing.

**NOMA-empowered ISAC.** In power-domain NOMA, users are served with different power levels and decode in a prescribed order using SIC. The decoding order and power allocation directly affect both the achievable rates and the structure of the transmitted signal used for sensing; hence, joint design of beamforming, power allocation, and scheduling is necessary [104]. Xie et al. develop a NOMA-empowered ISAC system where the BS serves multiple communication users and simultaneously senses a UAV [105]. The transmitted signal is $s\left(t;\tau \right)=u\left[t\right]g\left(\tau \right)\sum_{n=1}^{N}\alpha_{n}x_{n}$, with beamformer u[t], pulse g(τ), and power allocation $\left\{\alpha_{n}\right\}$. A joint beamforming, power allocation, and rate adaptation strategy is designed to minimize average delay while meeting sensing capability using a queue-aware cross-layer formulation and a Newton-based inner solver with linear programming for rate adaptation. Dou et al. consider sensing-efficient NOMA-aided ISAC with joint sensing scheduling and beamforming optimization to maximize sensing performance under communication quality-of-service (QoS) [58]. Wang et al. optimize transmit power in cooperative NOMA-aided secure ISAC with simultaneous wireless information and power transfer (SWIPT), balancing secrecy, sensing, and energy harvesting [106]. These works show that NOMA can support multi-user ISAC with manageable complexity when decoding order and power allocation are optimized.

**RSMA for ISAC.** In RSMA, messages are split into common and private parts; the common stream is decoded by all users and the private streams by the intended users, offering flexibility in interference management. Yin et al. introduce an RSMA-assisted ISAC architecture where the ISAC node simultaneously communicates with downlink users and probes a moving target [107]. They formulate a waveform design problem that minimizes the CRB for target estimation and maximizes the minimum fairness rate (MFR) among users under a per-antenna power constraint. The optimization maximizes a weighted combination of the minimum user rate (common plus private) and an auxiliary variable *t*, subject to an FIM constraint F≥tI ensuring minimum sensing accuracy, per-antenna power constraints, and common/private rate consistency. The problem is non-convex; it is transformed to a semidefinite programming (SDP) form and solved via SCA. Simulations show that RSMA-assisted ISAC achieves a better communication–sensing trade-off than space-division multiple access (SDMA) and NOMA baselines in both terrestrial and satellite scenarios. Niu et al. note that in NOMA-inspired downlink ISAC, additional sensing signals can be overlaid and then removed at the UE via SIC so that the same waveform serves both functions with reduced interfunction interference [108]. Dizdar et al. study energy-efficient DFRC with RSMA, low-resolution digital-to-analog converters (DACs), and RF chain selection; the transmit signal is $x=p_{c}s_{c}+\sum_{k=1}^{K}p_{k}s_{k}$, with common stream $s_{c}$ and private streams $s_{k}$, and the design accounts for hardware power consumption and imperfect channel state information at the transmitter (CSIT) [109]. RSMA is thus a promising multiple access scheme for ISAC when both multi-user fairness and sensing accuracy are required. Compared with orthogonal multiple access (OMA), NOMA and RSMA allow more users to be served on the same time-frequency resources at the cost of increased receiver complexity and sensitivity to imperfect CSI; in ISAC, the common stream in RSMA can also be exploited for sensing without introducing extra dedicated radar waveforms, which reduces interfunction interference and simplifies the waveform design.

### 4.5. Power and Resource Allocation at PHY Level

Efficient allocation of power and of time-frequency-spatial resources is essential to meet both communication and sensing requirements under shared hardware and spectrum. Because communication and sensing are evaluated by different metrics (e.g., rate vs. CRB or detection probability), the resource allocation problem is inherently multi-objective; common approaches include weighted-sum optimization, constraint-based formulation (optimize one metric while constraining the other), or Pareto-front computation.

**Power allocation under sensing and communication constraints.** A common formulation is to maximize a communication metric (e.g., sum rate or minimum rate) subject to a sensing constraint (e.g., minimum SNR or CRB), or vice versa, under a total or per-antenna power budget [28]. For OTFS-aided cell-free MIMO ISAC, Singh et al. maximize sensing SNR subject to per-access point (AP) power limits and per-user equipment (UE) SINR constraints [52]. The non-convex SINR constraints are rewritten as second-order cone (SOC) constraints, and the problem is solved iteratively. Alternatively, the sensing performance can be constrained while maximizing a communication objective: $\max_{R}C\left(R\right)$ subject to $S\left(R\right)\geq S_{\mathrm{th}}$ and T(R), where C, S denote communication and sensing metrics and T is the constraint set. Zhang et al. consider joint fronthaul compression and power allocation for networked ISAC, optimizing over compression ratios and transmit power to balance backhaul load and sensing/communication performance [60]. Wei addresses low-latency ISAC optimization for 6G V2X: sensing latency is minimized while preserving detection probability and communication quality; analytical detection thresholds and repetition bounds are derived for UAV and vehicular cases, and branch-and-bound is used for mixed-integer programs with much lower complexity than grid search [77]. These formulations often lead to non-convex or mixed-integer problems; solutions rely on convex relaxation, AO, or learning-based methods.

**Resource allocation in V2X and multi-cell ISAC.** Annu and Rajalakshmi survey resource allocation for 6G V2X sidelink, including mathematical formulations and challenges relevant to ISAC-capable sidelink [13]. In their study of cooperative sensing in uplink ISAC with multi-user waveform optimization, Li et al. propose joint subcarrier and power allocation (JSPA), which uses water-filling for the communication subproblem under a rate threshold and distributes subcarriers and power to sensing and communication subsystems [110]. In an IoV setting enabled by digital twins, Gong et al. examine resource allocation for ISAC, coordinating communication and sensing resources throughout the network [111]. With deep reinforcement learning (DRL) or other learning-based techniques for real-time adaptation, sub-channel and power distribution among BSs in multi-cell or coordinated multi-point (CoMP) ISAC can be tuned to maximize sum rate under QoS and CRB restrictions [25]. ISAC-capable nodes must allocate time-frequency resources for data and sensing (e.g., reference signals or dedicated sensing slots) while meeting latency and reliability requirements for safety messages [13]. Another aspect of the joint fronthaul compression and power allocation problem in networked ISAC is that when multiple BSs or RSUs collaborate in sensing and communication, the backhaul links restrict the amount of sensing and channel information that can be shared; end-to-end performance is enhanced by optimizing compression and power jointly [60]. All things considered, PHY-level resource allocation for ISAC-V2X is still a multi-objective, non-convex problem with ongoing research into scalable algorithms and Pareto-optimal or constraint-based formulations.

### 4.6. Sensing–Communication Trade-Off Analysis

The coexistence of sensing and communication on the same resources creates a fundamental trade-off: improving one typically comes at the expense of the other. Characterizing and optimizing this trade-off is key for system design and standardization. The trade-off arises because the same transmit power, time, and bandwidth must be shared; a waveform or beamformer optimized purely for communication (e.g., maximum sum rate) generally does not maximize sensing accuracy (e.g., minimum CRB or maximum detection SNR), and vice versa. Joint design and Pareto analysis allow system designers to select operating points that meet both communication and sensing requirements for a given scenario.

**Figure 11:**
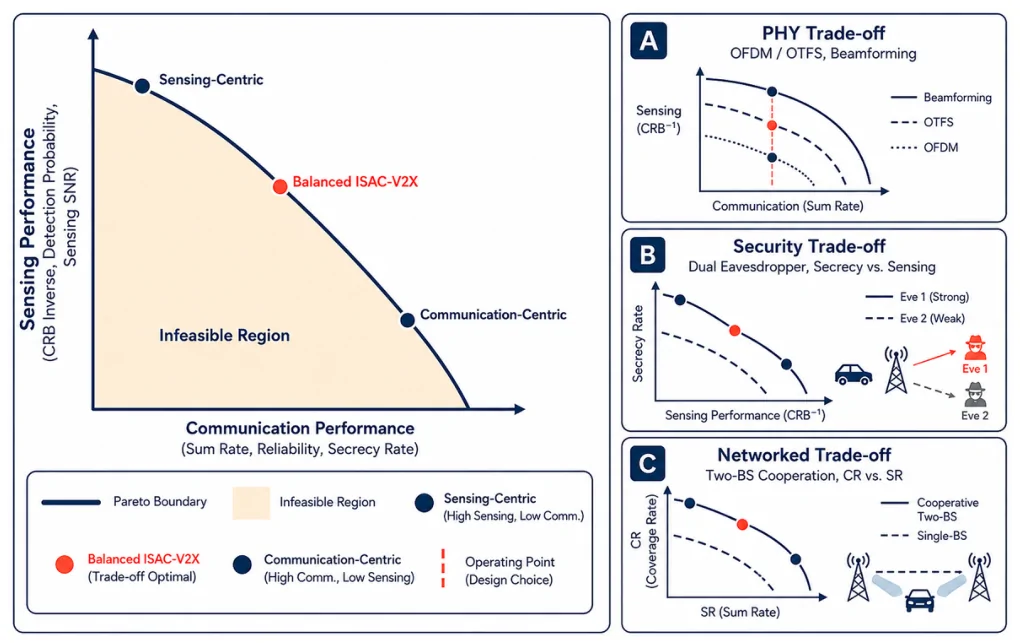
Fundamental sensing–communication trade-off in ISAC-V2X, illustrated through representative Pareto-optimal operating points.

Figure 11 illustrates the Pareto-style sensing–communication trade-off view used throughout this survey to interpret design choices and standardization operating points.

**Pareto frontier and weighted-sum formulations.** The trade-off is often represented as a Pareto frontier between a communication metric (e.g., rate, SINR) and a sensing metric (e.g., CRB, detection probability, SNR) [31,68]. Magbool et al. describe a generic multi-objective form: maximize G(R)=ρC(R)+(1−ρ)S(R) over resources R subject to constraints T(R), where C and S are communication and sensing metrics and ρ∈[0,1] is a trade-off weight; varying ρ yields the Pareto front [28]. The two corner points of the frontier correspond to communication-only optimal ($P_{\mathrm{CS}}$) and sensing-only optimal ($P_{\mathrm{SC}}$) designs; any point on the curve is Pareto-optimal in the sense that improving one metric would worsen the other [31]. Alternatively, one objective is optimized while the other is constrained (e.g., maximize rate subject to CRB ≤ϵ or maximize detection probability subject to minimum rate). Keskin et al. provide a holistic treatment of fundamental trade-offs in monostatic ISAC toward 6G, relating waveform and resource choices to the Pareto frontier of sensing accuracy versus communication rate [68]. Liu et al. survey fundamental limits of ISAC and discuss the Pareto-optimal rate-distortion or rate-CRB regions [34].

**Detection probability, CRB, and latency.** In safety-critical V2X, detection probability and latency are as important as rate. Wei analyzes low-latency ISAC for 6G V2X and derives detection thresholds and repetition bounds under antenna and RCS imperfections; the trade-off between detection probability, block length, and communication quality is made explicit [77]. Beam misalignment, precoder imperfections, and fluctuating radar cross-sections increase sensing latency; robust optimization and branch-and-bound for mixed-integer programs can minimize latency while preserving detection probability and communication QoS. Niu et al. examine interference management in ISAC and talk about how RSMA-based and NOMA-inspired designs might improve the achievable trade-off region by reducing mutual interference between sensing and communication [108]. For sensing accuracy; CRB-based formulations are frequently used: subject to communication and power limits, the transmit covariance or waveform is designed to reduce CRB (or maximize Fisher information) [10,107]. Zhao et al. trace the trade-off between communication SINR and sensing SNR in MF-RIS assisted systems via Pareto optimization in RIS-assisted ISAC [101]. These analyses assist operators and standards organizations in selecting operational points (such as ρ or constraint levels) based on service requirements and scenarios (such as highway versus urban). The Pareto frontier is actually tracked by repeatedly solving weighted-sum or constraint-based optimizations for various weights or thresholds because it is not known in closed form. Practical waveform and resource allocation strategies can be compared to theoretical boundaries provided by the fundamental limits of ISAC, such as the capacity-distortion or rate-CRB regions [34]. Maintaining a minimum detection probability while ensuring a minimum data rate or latency for cooperative awareness messages is a common requirement for vehicular safety applications.

### 4.7. Summary of PHY Techniques

The sensing–communication trade-off and Pareto-optimal operating points guide the choice of parameters and standards. The primary ISAC-V2X techniques show significantly differing computational and scalability properties from the standpoint of actual vehicle deployment. Because OFDM-based ISAC is compatible with the current NR-V2X infrastructure and benefits from sophisticated FFT-based processing, its computing requirements are comparatively low. On the other hand, OTFS increases receiver-side processing complexity at the expense of better robustness under high Doppler [112]. Fully digital beamforming offers high spatial flexibility but requires one RF chain per antenna and becomes increasingly costly and power-intensive for large vehicular arrays; hybrid analog–digital beamforming therefore provides a more scalable compromise by reducing the number of RF chains while retaining multi-user and multi-target capabilities [65].

RIS and STAR-RIS-assisted ISAC can improve coverage and sensing performance without adding active RF chains at the surface, but their scalability is constrained by channel-estimation overhead and the growing dimensionality of joint BS-RIS optimization as the number of reflecting elements increases [113]. NOMA and RSMA-assisted ISAC improve spectral efficiency and multi-user connectivity, but SIC, rate-splitting, and joint power/beam allocation increase receiver and optimization complexity, particularly in dense V2X scenarios [11,58]. Networked and cell-free ISAC further improve coverage and cooperative sensing, although coordination, synchronization, fronthaul signaling, and multi-node resource optimization become increasingly demanding as the number of vehicles, RSUs, or APs grows [52,60]. In contrast, AI/ML-based approaches shift much of the computational burden to the training stage and can provide relatively fast online inference, making them attractive for low-latency vehicular operation, although their scalability depends on training-data availability, model size, robustness, and distributed communication overhead [61]. Overall, no single approach is uniformly preferable: practical ISAC-V2X deployment requires balancing sensing and communication performance against processing latency, hardware cost, signaling overhead, and network scale. A qualitative comparison of the computational complexity, scalability, main complexity drivers, and practical vehicular suitability of these approaches is summarized in Table 5. However, the same radio interface and sensing outputs that enable new services also introduce security and privacy risks: adversarial access to sensing data, spoofing of range/velocity, and inference of location or motion. Section 5 therefore turns to security and privacy in ISAC-V2X, including physical-layer security (PLS), attack models, and countermeasures, and how they interact with the physical-layer techniques surveyed in this section. These findings primarily motivate G1 and G5, since practical ISAC-V2X still requires scalable joint optimization of waveforms, beamforming, RIS configurations, and radio resources under high mobility, interference, and implementation-complexity constraints. The security implications of shared sensing and communication resources further connect these limitations to G2.

## 5. Security and Privacy in ISAC-V2X

The integration of sensing and communication in V2X introduces distinct security and privacy challenges beyond those of conventional cellular or radar-only systems [7,34]. The attack surface is increased by dual-functional waveforms and shared hardware: although sensing echoes and radio environment data can reveal position, identity, or behavior, the same signal-carrying data can be used for spoofing or eavesdropping. ISAC and security are specifically mentioned as important research areas in 6G and B5G V2X standards and roadmaps [3,49]. With a focus on work that is directly applicable to vehicular and 6G V2X scenarios, this section examines PLS for ISAC-V2X, attack surfaces (spoofing, jamming, eavesdropping), privacy risks from sensing data leakage, secure beamforming and waveform design, AI/ML-based security threats and defenses, and security–privacy trade-offs. The treatment draws on the channel and physical-layer foundations of Section 3 and Section 4 and prepares the transition to networked ISAC-V2X architectures and resource management in Section 6.

**Figure 12:**
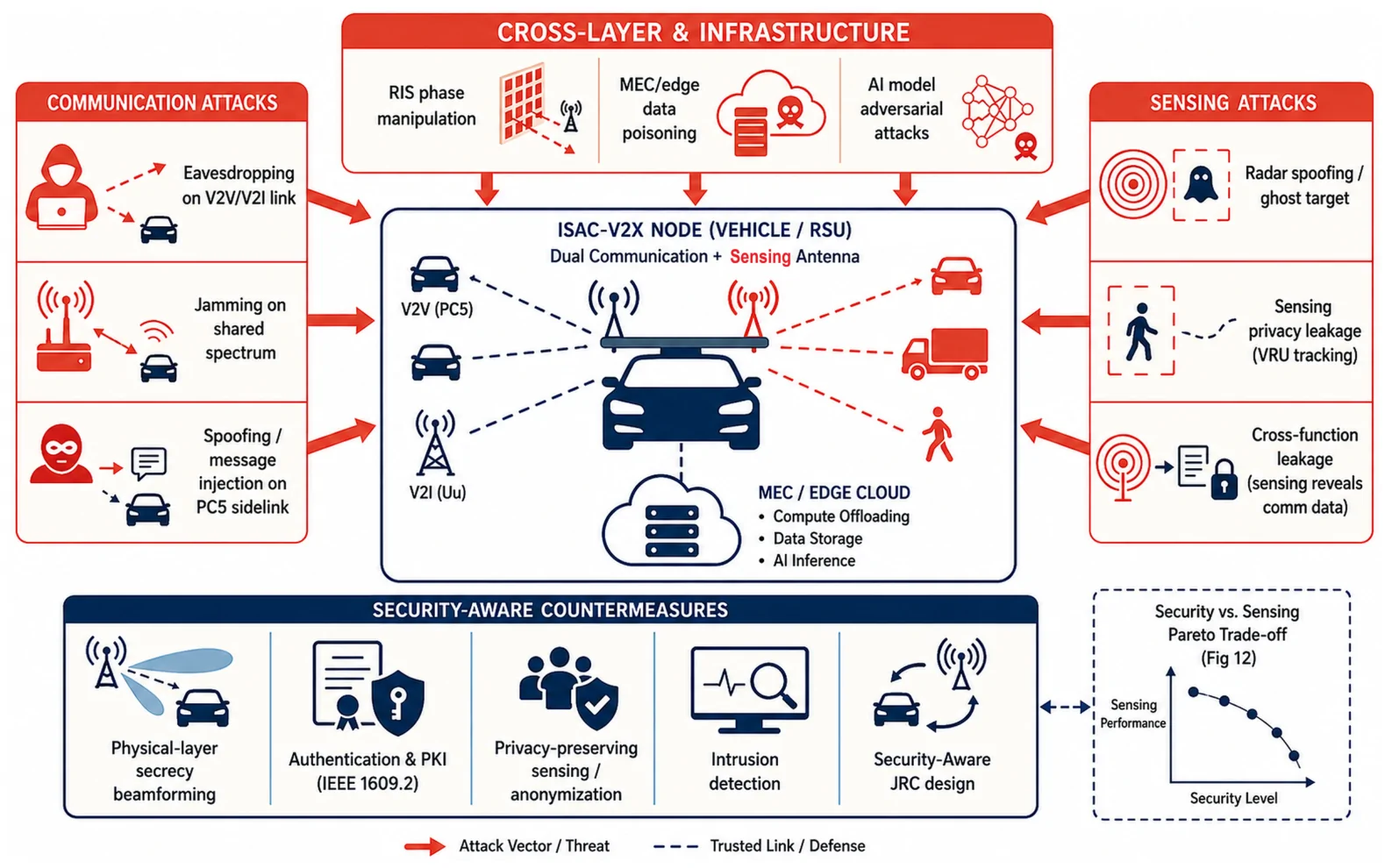
Security and attack surfaces in ISAC-V2X, highlighting communication and sensing threats together with representative countermeasures.

Figure 12 summarizes a representative ISAC-V2X threat model, highlighting how communication and sensing functions broaden the adversary surface (eavesdropping, spoofing/manipulation, and jamming/disruption).

### 5.1. Physical Layer Security for ISAC-V2X

PLS exploits channel randomness, spatial degrees of freedom, and signal design to achieve confidentiality and resilience without relying solely on upper-layer cryptography. In ISAC, PLS must protect both the communication link and the sensing functionality: the former from eavesdropping and the latter from unauthorized extraction of target or environment information. Zhu et al. survey secure ISAC in 6G and position PLS within a three-layer architecture (hardware, omniscient, application), emphasizing information-theoretic security, sensing-assisted interference, and software-defined communication for intelligent connectivity [26]. They categorize security indices for ISAC (sensing, communication, computation, security, and unified metrics such as Cramér–Rao lower bound (CRLB) and mutual information) and discuss how beamforming and intelligent antenna arrays synergize with information theory to enhance secure ISAC. Guo et al. provide a comprehensive review of RIS-assisted PLS, including secrecy rate, secrecy capacity, and secrecy outage probability; the same metrics apply when RIS is integrated with ISAC to reshape channels for legitimate users while degrading the wiretap channel [114]. Secrecy capacity is defined as the positive part of the difference between the legitimate and wiretap link capacities; secrecy outage probability (SOP) is the probability that the instantaneous secrecy capacity falls below a target rate and is used in delay-constrained or slow-fading settings. In ISAC, the same transmit covariance affects both the communication rate and the sensing beampattern, so the trade-off between maximizing secrecy rate and meeting sensing constraints (e.g., minimum gain at the target, maximum gain at the eavesdropper) must be explicitly handled in the optimization; the Pareto frontier of secrecy rate vs. sensing SNR (or vs. sensing security level) is a useful design tool. Khoshafa et al. survey RIS-assisted PLS in RF and optical wireless systems, covering principles and applications that extend to ISAC deployments where RIS supports both communication and sensing [115].

In UAV-enabled secure ISAC, the mobility of the aerial node adds a degree of freedom for PLS. Yao et al. study a system where a UAV acts as a dual-functional BS serving a legitimate user and a sensing target in the presence of a dual-functional eavesdropper that intercepts both communication and sensing [116]. They maximize the average achievable secrecy rate by jointly designing the UAV trajectory, information beamforming, and sensing beamforming under sensing performance, sensing security, power, and flight constraints. The secrecy rate at each slot is the positive part of the difference between the log-rates at the legitimate user and the eavesdropper. Sensing security is enforced by upper-bounding the transmit beampattern gain at the eavesdropper and requiring a minimum beampattern gain at the target. The non-convex problem is solved via AO with SCA and SDR. A notable trade-off is that the radar (sensing) signal, which acts as interference to the eavesdropper for communication, becomes the desired signal for a sensing eavesdropper; thus communication and sensing security cannot be optimized independently. Wu et al. address real-time trajectory design for UAV-enabled secure ISAC when the legitimate user (Bob) moves and the eavesdropper follows a known trajectory [117]. They use extended Kalman filtering (EKF) on delay measurements from ISAC echoes to track and predict Bob’s location and then optimize the UAV trajectory to maximize the real-time secrecy rate $R_{s}\left[n\right]=R_{b}\left[n\right]-R_{w}\left[n\right]$ under speed constraints, via SCA. Xu et al. combine RIS and UAV for secure ISAC: passive beamforming at the aerial RIS enhances the legitimate link and suppresses jamming, while artificial noise (AN) can be directed toward the eavesdropper; they compare continuous and discrete phase shifts and show that joint active/passive beamforming and IRS-UAV deployment significantly improve anti-jamming SINR and secrecy [118].

Cooperative and multi-antenna structures further improve PLS. Wang et al. consider cooperative NOMA (C-NOMA) with SWIPT in secure ISAC: a cell-center user harvests energy and relays the cell-edge user’s message while the BS transmits a sensing signal that also acts as AN [106]. Under imperfect wiretap CSI (LoS and path loss known, multi-path bounded), they minimize transmit power subject to data rate, eavesdropping rate, and echo SINR constraints. The formulation uses S-Procedure and SDR to handle the uncertainty, and a BCD algorithm iterates between beamforming/AN covariance and power-splitting/auxiliary variables. The two-phase C-NOMA structure allows AN to be injected in both phases, improving flexibility; the relay phase is power-limited by the harvested energy, and U1 can also send AN along with the relayed message to limit the eavesdropper’s rate on the U2 link. Liu et al. maximize the sum secrecy rate in an RIS-assisted secure ISAC system where the radar target is a potential eavesdropper; they jointly design transmit beamforming, AN covariance, and RIS phase shifts under a beampattern matching constraint [119]. The non-convex coupled problem is solved with an SAC DRL framework, with the reward defined as the sum of per-user secrecy rates ${\left[R_{k}-R_{k}^{e}\right]}^{+}$; SAC outperforms DDPG and benchmarks without AN or with random RIS phases. The Markov decision process (MDP) state includes beam pattern error, CSI, and user/eavesdropper rates; the action space comprises beamforming vectors, RIS phases, and AN, and the critic uses two Q-networks to reduce overestimation. Deng et al. study covert communications in ISAC: the UAV maximizes the achievable covert rate (ACR) while keeping the probability of detection at wardens below a threshold; the sensing signal masks the information signal. They derive the minimum detection error probability at the wardens and use BCD to alternate between beamforming and trajectory optimization [120].

Secure ISAC-V2X design usually necessitates the joint optimization of communication secrecy, sensing quality, mobility, and transmit resources, as stated in Table 6. While learning-based techniques offer an alternative for highly dynamic or large-dimensional setups, optimization-based techniques like SCA, SDR, and BCD continue to be the most popular. This comparison emphasizes how security improvements must be balanced with computational complexity and real-time vehicle limits.

### 5.2. Attack Surfaces in ISAC (Spoofing, Jamming, Eavesdropping)

Because a single physical layer may enable both data transmission and sensing, and because the same waveforms can be used for several sorts of attacks, ISAC expands the attack surface. Furqan et al. separate communication-oriented dangers from sensing-oriented ones by analyzing wireless communication, sensing, and radio environment mapping (REM) from a security standpoint. While eavesdropping in communication focuses on the message’s content, exploratory attacks in sensing/REM seek to gather radio and environment awareness from other people’s signals. While manipulation in sensing/REM results in inaccurate sensing and flawed REM (e.g., incorrect maps or resource decisions), spoofing in communication wastes or steals resources. Jamming interferes with communication; disruption is a more general term that encompasses both jamming and flooding of sensing or processing nodes [33]. These distinctions are important for ISAC-V2X: an attacker may target only communication, only sensing, or both.

**Eavesdropping.** In ISAC, the eavesdropper may be a communication-only listener or a dual-functional node that also exploits the sensing waveform. Yao et al. model a dual-functional eavesdropper that intercepts both the data and the sensing signal; the sensing signal acts as AN for communication but as useful signal for sensing interception, creating a trade-off between communication and sensing security [116]. Ren et al. consider secure cell-free ISAC with both information and sensing eavesdroppers and maximize mutual information at the authorized sensing receiver while capping the eavesdropper’s mutual information. When the communication user itself acts as a sensing eavesdropper (e.g., a vehicle that is served for communication but also tries to infer target or environment information from the radar waveform), the design goal can be to maximize the mutual information at the authorized sensing receiver while keeping the eavesdropper’s mutual information below a threshold; this sensing–security formulation is considered in secure cell-free ISAC with both information and sensing eavesdroppers. Robust beamforming under imperfect or bounded eavesdropper CSI is treated in [106] via S-Procedure and in [120] for covert ISAC with multiple wardens. In V2X, the high mobility of vehicles and the need for low-latency safety messages make it difficult to obtain accurate and timely CSI for potential eavesdroppers; hence, worst-case or bounded-CSI formulations and learning-based policies that adapt to channel statistics are of practical interest.

**Jamming.** Jamming degrades both communication and radar reception. Shu et al. address ISAC in the presence of interrupted-sampling repeater jamming (ISRJ), which generates false targets and degrades detection [122]. They jointly design the transmit waveform and receive filters (matched and mismatched) to minimize multi-user interference (MUI) subject to target mainlobe level, jamming mainlobe upper bound, peak sidelobe level, and transmit power. The receive filter output mainlobe and sidelobes are constrained so that the target is detectable while the jamming component is suppressed; the problem is solved with the alternating direction method of multipliers (ADMM). Xu et al. consider a malicious jammer that blocks the direct BS-target link; IRS-UAV provides an alternative LoS path, and passive beamforming is optimized jointly with active beamforming and deployment to maximize sum rate under echo SINR and anti-jamming constraints [118]. Naeem et al. review security and privacy for RIS in 6G and note that RIS itself can be abused: an attacker may use an “illegal” RIS to boost the eavesdropping link or to relay jamming toward legitimate receivers [121].

**Spoofing and manipulation.** Spoofing in V2X often targets positioning or identity (e.g., fake BS or vehicle). In sensing, manipulation can inject false echoes or corrupt REM data, leading to wrong positioning or resource allocation. Furqan et al. describe REM manipulation and its consequences in a vehicular scenario: faulty REM can cause incorrect cell association, spectrum decisions, or handover. They also present a case study in which REM is used for cognitive wireless security in V2X: REM-assisted decisions can improve resource allocation and handover, but if the REM is spoofed or manipulated, the same mechanism can be exploited to steer traffic toward malicious nodes or to cause denial of service. Performance metrics for spoofing/manipulation defense include false alarm and missed detection rates and the receiver operating characteristic (ROC) curve; the same classification view applies to jamming/disruption detection [33]. Nencioni et al. survey security and dependability in 5G MEC, which is relevant when ISAC processing or REM aggregation is performed at the edge in V2X; edge nodes become attractive targets for jamming or compromise and must be protected by authentication, secure interfaces, and redundancy [47].

### 5.3. Privacy Risks from Sensing Data Leakage

Sensing and REM generate fine-grained data about the environment and users (location, motion, occupancy), which raises privacy concerns. Furqan et al. argue that REM and wireless sensing are prone to exploratory attacks that violate the privacy of both users and the network (e.g., inferring mobility patterns or social behavior from radio fingerprints) [33]. They also note that key-based or cryptographic protection of content does not directly address sensing/REM security; PLS and link-level design are better suited to limit what an adversary can infer from the physical signal. In V2X, REM can be used for handover prediction, content caching, and resource allocation; if REM data are manipulated or leaked, safety and efficiency are compromised. The distinction between communication security (confidentiality of the message) and sensing/REM security (confidentiality and integrity of the inferred state) is therefore central: the former is often measured by secrecy rate or BER gap, while the latter can be measured by estimation error at the adversary or by the sensitivity of REM outputs to unauthorized access.

Zheng et al. consider UAV individual identification in ISAC networks via distilled RF fingerprints and a large language model (LLM): the idea is to recognize UAVs from their RF signatures for authentication or monitoring [124]. These methods increase accountability, but they also show how RF sensing can expose identity and behavior; in the absence of appropriate safeguards, the same sensing data can be abused for monitoring or profiling. Tan and Zhu examine multi-modal sensing for intelligent V2X and talk about deployment issues. They point out that while combining camera, LiDAR, radar, and communication-based sensing improves situational awareness, it also increases the amount of sensitive data (like trajectories and occupancy) that needs to be anonymized or protected [8]. When RIS is integrated with ISAC, MEC, and IoT in 6G, Naeem et al. list privacy and security concerns as follows: centralized control of RIS may become a single point of failure or disclosure, and RIS phase configurations and channel estimates may leak information about the environment and user positions [121]. Anonymization of REM/sensing data, access control, and PLS are examples of mitigations that limit the adversary’s information gain even in the event that signals are overheard (e.g., via secrecy rate or estimation error metrics) [26,33]. One important topic that connects sensing security with data minimization and regulatory compliance for vehicular ISAC is the privacy-preserving aggregation of sensing or REM updates (e.g., from many RSUs or vehicles) without disclosing individual trajectories or identities.

### 5.4. Secure Beamforming and Waveform Design

Secure beamforming and waveform design in ISAC must satisfy both communication secrecy (or covertness) and sensing performance (e.g., beampattern gain, SINR, or estimation accuracy). A common approach is to use the sensing waveform as AN: it illuminates the target for radar while degrading the eavesdropper’s channel for communication.

**Secrecy rate and AN design.** The achievable secrecy rate for a user *k* is $R_{k}^{\sec}={\left[R_{k}-R_{k}^{e}\right]}^{+}$, where $R_{k}$ is the legitimate rate and $R_{k}^{e}$ the eavesdropper’s rate [125,126,127,128]. In RIS-assisted secure ISAC, the transmitted covariance is $R_{x}=\sum_{i=1}^{K}w_{i}w_{i}^{H}+R_{z}$ (beamforming plus AN), and the beampattern is $P_{\mathrm{bp}}=a^{H}\left(\theta \right)R_{x}a\left(\theta \right)$ [119]. The AN covariance $R_{z}$ is optimized jointly with beamforming and RIS phases to maximize $\sum_{k}R_{k}^{\sec}$ under power and beampattern matching constraints. In [116], the sensing covariance $A_{s}\left[n\right]$ plays the role of AN; the beampattern constraints $\zeta_{t}\left[n\right]\geq \Gamma_{t}d_{t}^{2}\left(\rho \left[n\right]\right)$ and $\zeta_{e}\left[n\right]\leq \Gamma_{e}d_{e}^{2}\left(\rho \left[n\right]\right)$ couple trajectory and beamforming. Guo et al. give the standard form for secrecy capacity in Gaussian channels, $C_{\sec}={\left(C_{u}-C_{e}\right)}^{+}$ with $C\left(x\right)=\log_{2}\left(1+x\right)$, and note that RIS can reduce the correlation between the legitimate and wiretap channels to improve $C_{\sec}$ [114].

**Covert communications.** When the goal is to hide the presence of communication (low probability of detection), the warden’s detection error probability is maximized. Deng et al. formulate the covert ISAC problem as maximizing the average ACR subject to a detection probability constraint at the wardens, plus power, speed, and sensing gain constraints [120]. They show that to improve covertness, the projection of the information beamforming covariance on the warden’s channel subspace should be reduced, or the projection of the sensing beamforming covariance should be increased; i.e., the sensing signal helps mask the information signal. The joint beamforming-trajectory problem is solved by BCD. In contrast to traditional PLS, which aims to achieve a positive secrecy rate (legitimate rate minus eavesdropper rate), covert communication seeks to make the transmission undetectable by the warden; the two objectives can be combined in scenarios where both low probability of detection and low probability of successful decoding at the eavesdropper are required.

**Waveform design under jamming.** Shu et al. design the ISAC transmit waveform (each column of X is a beamforming vector per time slot) and receive filters so that the target mainlobe at the filter output is above a threshold $\zeta_{l}$, the jamming mainlobe is below $\varepsilon_{l}$, and the peak sidelobe is below $\epsilon_{l}$ [122]. The MUI ${∥\Xi ∥}_{F}={∥HX-S∥}_{F}$ is minimized. For the mismatched filter (JTMMD), the receive filter is not constrained to be the matched filter, allowing better sidelobe and jamming suppression than the matched filter (JTMD); both schemes use ADMM for the joint update of X, v, and auxiliary variables. Joint waveform and beamforming design for ISAC under various constraints is also discussed in broader surveys on dual-functional radar–communication and ISAC signal processing [85,86].

**Optimization formulations.** A typical secure ISAC beamforming problem maximizes the sum of per-user secrecy rates (legitimate rate minus eavesdropper rate) over the beamforming matrix W, AN covariance $R_{z}$, and RIS phase matrix Φ, subject to a total power constraint, beampattern or sensing constraints (e.g., minimum gain toward the target, maximum gain toward the eavesdropper, or a beampattern matching error bound), and RIS feasibility $\Phi \in F_{\mathrm{RIS}}$. For example, with steering vector a(θ) and transmit covariance $R_{x}=WW^{H}+R_{z}$, the beampattern is $P\left(\theta \right)=a^{H}\left(\theta \right)R_{x}a\left(\theta \right)$; a minimum level at the target angle and a maximum level at the eavesdropper angle can be imposed. In [119], the beampattern matching constraint is written as a mean-square error between the designed beampattern and a desired one over a grid of angles, with an upper bound ϵ; the scaling factor for the desired pattern is obtained in closed form from a first-order optimality condition. These problems are non-convex and are handled by SDR, SCA, AO, or DRL as in [56]. When the problem includes trajectory (e.g., UAV), the channel and steering vectors become functions of position, and the constraints (e.g., sensing gain and sensing security) are trajectory-dependent, leading to joint trajectory–beamforming formulations as in [116,120].

### 5.5. AI/ML-Based Security Threats and Defenses

AI/ML is used in ISAC for channel estimation, beamforming, and target recognition but also introduces new threats: adversarial manipulation of learning-based systems and privacy leakage from trained models or sensed data.

**Threats.** Zhu et al. note that malicious users can use adversarial techniques to manipulate AI algorithms, leading to inaccurate sensing and thus to wrong communication or control decisions [26]. In their assessment of 6G powered by generative AI (GenAI), Sheraz et al. address security and privacy issues with generative AI in wireless systems, such as data poisoning and deepfakes [29]. In their assessment of ML-powered 6G networks, Noman et al. point out that ML models and data pipelines are vulnerable to attacks (e.g., model inversion, membership inference), which is important when ISAC employs learning for beamforming or sensing [129]. Adversarial examples or corrupted training data can impair detection or communication performance according to Temiz et al.’s survey of DL for ISAC [61]. In V2X, if the ISAC system relies on learned models for object detection, tracking, or beam prediction, an adversary that can perturb the input (e.g., adversarial patches or spoofed echoes) or the training data may cause misdetection or wrong beam selection, with potential safety implications; hence, robustness and interpretability of AI/ML components in ISAC-V2X are important research directions.

**Defenses.** AI/ML is also used for defense. Liu et al. use SAC-based DRL to optimize beamforming, AN, and RIS phases for secrecy rate maximization in RIS-assisted secure ISAC, avoiding explicit convex reformulation and scaling to time-varying channels [119]. Zhu et al. apply DDPG and SAC to STAR-RIS-aided MISO ISAC secure communications with energy-splitting and time-switching protocols; the long-term average secrecy rate is maximized under echo SNR and rate constraints, and the SAC-based solution handles channel dynamics better than one-shot convex methods [56]. The sensed target and legitimate users lie on opposite sides of the STAR-RIS, so the transmitting and reflecting coefficients are jointly optimized to serve communication while limiting information leakage to the target (acting as eavesdropper). Wu et al. use EKF to track the legitimate user from ISAC delay measurements and then optimize the UAV trajectory for secrecy; the sensing function directly supports the security function [117]. Federated learning (FL) and privacy-preserving aggregation can reduce the exposure of raw sensing or REM data while still allowing model updates [26]. Robust training (e.g., adversarial training, certified defenses) and anomaly detection for sensing outputs are promising directions for securing AI/ML-based ISAC-V2X [61,129]. Merluzzi et al. outline the Hexa-X vision for AI/ML-driven 6G communication and sensing, which includes the need for trustworthy and secure AI in integrated systems [49].

### 5.6. Security–Privacy Trade-Offs in V2X

In ISAC-V2X, security and privacy objectives can conflict with each other and with communication/sensing performance.

**Communication vs. sensing security.** Yao et al. show that the radar (sensing) signal is desired for a sensing eavesdropper but acts as AN for communication; imposing a sensing security constraint (upper bound on beampattern at the eavesdropper) reduces the achievable secrecy rate compared with a design that ignores sensing security [116]. Thus, there is a trade-off between communication secrecy and sensing confidentiality. Ren et al. jointly consider both types of security in cell-free ISAC.

**Covertness vs. rate and sensing.** In covert ISAC, the need to keep the detection probability low at the wardens limits how much power can be put into the information beam; the sensing signal helps covertness but consumes power that could otherwise go to communication or to stronger target illumination [120]. So covertness, communication rate, and sensing quality are three-way coupled.

**PLS vs. privacy.** PLS (such as secrecy rate and AN) can restrict what an eavesdropper can learn from the signal and safeguard communication content. The inference of user or environment attributes (location, identity, behavior) from sensed or mapped data is the subject of privacy in sensing/REM. Strong PLS can lessen the amount of information spilled over the wiretap channel, but access control and data minimization are required to safeguard privacy if sensing data or REM are shared or stored (e.g., at MEC or on the cloud) [33,121]. Regulatory and standards bodies (e.g., 3GPP, IEEE 802.11bf for WLAN sensing) are increasingly addressing privacy in positioning and sensing; ISAC-V2X deployments will need to align with such requirements while still delivering the sensing and communication performance needed for safety and automation [3].

**Resource and complexity trade-offs.** The constraints (sensing security, covertness, privacy budgets, etc.) are frequently added in secure and privacy-preserving design, which can raise the transmit power, lower rate, or necessitate extra signaling and iterations. According to Naeem et al., RIS deployment for PLS can lessen the requirement for excessive AN power, but if the RIS is compromised, it adds control and channel estimate overhead as well as new attack surfaces [121]. When security limitations are applied to the joint waveform–beamforming problem [88], wideband and multi-antenna ISAC solutions encounter comparable trade-offs. The choice of PLS technique (e.g., full optimization vs. heuristic or learning-based policies) should take security and real-time constraints into consideration because latency and reliability requirements in vehicular ISAC may conflict with the additional computation and feedback required for secure beamforming and AN design [7].

The ISAC-V2X attack taxonomy is summarized in Table 7. The contrast demonstrates that ISAC creates attack surfaces that impact sensing and communication at the same time. While sensing-oriented attacks can also corrupt environmental awareness, location, and REM information, communication-oriented attacks primarily risk confidentiality and availability. Therefore, rather than relying on a single defense method, effective protection necessitates complementing physical-layer, authentication, integrity, and edge-security mechanisms.

**Research directions.** Formal privacy guarantees for sensing-derived REM and trajectory data; tighter integration of PLS with authentication, key management, and V2X protocols; scalable and low-latency secure beamforming and waveform design for large-scale and cell-free ISAC; and unified metrics that jointly capture communication secrecy and sensing security are some of the major open issues. In addition to experimental validation of secure ISAC and systematic benchmarking of AI/ML-based solutions against optimization-based baselines, more work is required on robustness to adaptive jamming and combined eavesdropping–jamming assaults. For networked and multi-stakeholder ISAC-V2X systems to be deployed securely, several problems must be resolved.

### 5.7. Summary and Transition to Section 6

Section 5 showed that security and privacy in ISAC-V2X are tightly coupled with sensing and communication design. Key challenges include eavesdropping, jamming, spoofing, sensing-data leakage, secure beamforming and waveform design, and AI/ML-related threats, while PLS, AN, RIS-assisted optimization, and privacy-aware processing provide complementary countermeasures. Table 6 and Table 7 summarize the representative methods and attack taxonomy. Section 6 therefore extends these considerations to networked ISAC-V2X architectures, where multi-node cooperation, shared spectrum, fronthaul/backhaul constraints, and joint resource allocation further couple communication, sensing, and security. These findings primarily motivate G2 and G5, since secure ISAC-V2X requires joint treatment of sensing security, communication confidentiality, privacy, AI robustness, and scalable protection mechanisms. The need for common security interfaces, evaluation procedures, and practical validation also links these limitations to G3 and G4.

## 6. Networked ISAC-V2X Architectures and Resource Management

The networked architectures are a logical next step to support smart cities, autonomous driving, and aerial connectivity [3,49]. Standards and research roadmaps for 6G and B5G clearly incorporate integrated sensing and communication as well as V2X evolution. With a focus on formulations, trade-offs, and methods directly applicable to V2X and 6G, this section examines multi-BS/RSU cooperative ISAC, cell-free and distributed architectures, fronthaul/backhaul constraints and compression, edge/cloud integration, joint resource allocation, mobility management, and handover.

### 6.1. Multi-BS/RSU Cooperative ISAC for V2X

The sensing range, angular coverage, and blockage resistance of a single-BS ISAC are constrained. By utilizing the current cellular and RSU infrastructure, multi-BS cooperative sensing increases detection probability, increases range, and combines observations from several perspectives. A framework for ISAC-enabled multi-BS cooperative sensing (ISAC-MCS) toward 6G is proposed by Wei et al. [59], which divides cooperation into cooperative active, cooperative passive, and cooperative active–passive sensing as well as data-level and signal-level fusion. In data-level fusion, each BS reports local sensing results (e.g., detected positions or tracks) to a fusion center, which then combines them with lower fronthaul demand but limited accuracy gain. In signal-level fusion, raw or preprocessed echo signals from multiple BSs are coherently combined at a fusion center, yielding higher SNR and better estimation accuracy but requiring stricter time and phase synchronization and higher fronthaul capacity. For V2X, the framework is instantiated in a layered structure: terminal layer (micro-BSs and UEs), edge layer (macro-BSs and MEC servers), and cloud layer for global aggregation and decision-making. Key challenges include unified metrics for sensing and communication, interference between BSs, and synchronization suitable for mobile systems rather than dedicated radar. In cooperative active sensing, all BSs send and receive their own echoes and forward them to the fusion center; in cooperative passive sensing, one or more BSs receive echoes from signals transmitted by other BSs. The latter increases the number of observation links but requires accurate time and phase alignment across BSs. Synchronization in cellular systems is typically at the symbol or frame level (e.g., sub-microsecond), whereas coherent signal-level radar fusion often demands nanosecond-level accuracy. Symbol-level fusion and data-level fusion are therefore more practical for deployment on existing infrastructure, with a clear accuracy–complexity trade-off.

Wei et al. also introduce symbol-level multi-BS cooperative sensing, where demodulation symbols from multiple BSs are used for fusion instead of raw waveforms, relaxing synchronization to the symbol level of the communication system [130]. Phase calibration preserves distance and velocity information in the symbol stream; after compensation and coherent combination, distance and velocity estimation accuracy improve substantially compared with single-BS and data-level fusion (e.g., reported gains of about 40% in distance and 72% in velocity at −5 dB SNR versus single-BS, and 12% and 63% versus data-level ML-based fusion). This makes symbol-level fusion a practical option for deployment on existing cellular timelines.

Yin et al. address cooperative ISAC from an information-theoretic perspective for the two-BS case [131]. They define a cooperative sensing rate (SR) by modeling the sensing process as a virtual MIMO system: multiple BSs illuminate a static point target, and the reflected signals are observed at the BSs. Under Gaussian signaling and known steering, the cooperative SR has a closed form; maximizing it is equivalent to having all BSs perform maximum-ratio transmission (MRT) toward the target and to minimizing the MSE of the target’s RCS. For two BSs jointly serving one user and sensing one target, the trade-off between cooperative communication rate (CR) and cooperative SR is formulated as a weighted sum optimization and solved via SDP. The formulation shows that cooperative SR aligns both BSs’ beampatterns toward the target while CR favors user-directed beamforming, so the Pareto frontier between CR and SR guides system design.

Zhang et al. study CoMP ISAC under timing asynchrony across transceivers [90]. Asynchrony degrades both coherent sensing and coordinated communication. They propose a two-stage scheme: first, asynchronous errors and UE (target) locations are jointly estimated from coordinated sensing echoes; then, the estimated timing errors are used to correct the downlink CoMP transmission so that signals from multiple TRPs arrive quasi-synchronously at each UE. Closed-form CRBs for joint estimation and asymptotic communication rate under residual asynchrony are derived. A two-stage optimization then designs the coordinated sensing waveform (via Schur-complement-based SDR) and communication beamforming (via conjugate gradient with penalty terms) to improve robustness to asynchrony. The work is directly relevant to multi-BS/RSU ISAC-V2X, where backhaul/fronthaul and local oscillators introduce timing offsets.

Qu et al. consider multi-BS cooperative ISAC with rotatable arrays (RAs) at each BS [132]. By rotating the array as a whole (e.g., via a motor), additional spatial degrees of freedom are obtained with minimal hardware change. They jointly optimize the rotation angles of all BS arrays and the multi-BS beamforming matrix to minimize beampattern matching error while satisfying communication SINR and power constraints. The non-convex problem is handled by AO: SDR for beamforming and quasi-Newton for rotation angles. The RA-enabled multi-BS cooperative ISAC (RAMC-ISAC) design achieves lower beampattern error than fixed-array cooperation under the same communication requirements, illustrating the benefit of movable antenna structures in networked ISAC.

Wang et al. study ISAC-enabled cooperative detection for cellular-connected UAV networks, where multiple BSs perform joint detection of aerial targets [133]. The setup connects cooperative sensing with UAV traffic management and illustrates how multi-BS ISAC can serve both terrestrial V2X and aerial use cases. Pan et al. address cooperative trajectory planning and resource allocation for UAV-enabled ISAC, jointly optimizing UAV paths and communication/sensing resources to balance coverage and sensing quality [134]. These works extend the multi-BS cooperation idea to UAV-assisted and aerial scenarios relevant to future V2X and smart city deployments.

A simplified cooperation gain can be illustrated for the two-BS case. Let $R^{c}$ denote the cooperative communication rate and $R^{s}$ the cooperative sensing rate. For a single user and one target, with beamforming vectors $w_{1},w_{2}$ at the two BSs and steering vectors $a_{1},a_{2}$ toward the target, the cooperative SR scales with the combined gain $|a_{1}^{H}w_{1}+a_{2}^{H}w_{2}{|}^{2}$ when signals add coherently at the target. The trade-off between $R^{c}$ and $R^{s}$ is Pareto-limited; maximizing one typically reduces the other unless extra resources (e.g., power or bandwidth) are allocated. Unified metrics that combine communication and sensing in a single objective (e.g., weighted sum or constraint-based) are therefore important for multi-BS ISAC-V2X design [59,131].

### 6.2. Cell-Free and Distributed ISAC Architectures

**Figure 13:**
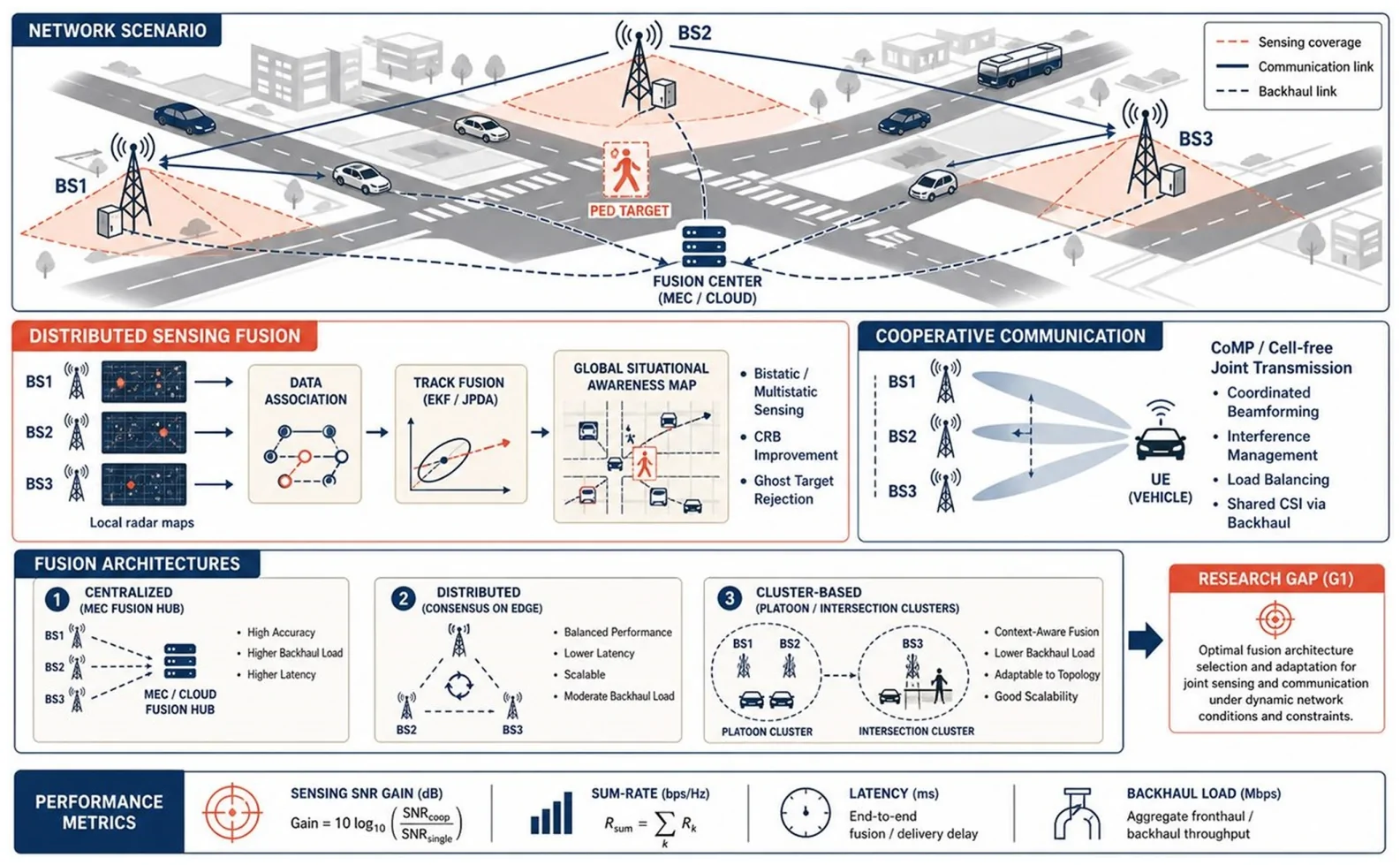
Multi-BS cooperative ISAC fusion architecture for networked ISAC-V2X, illustrating representative sensing fusion and cooperative communication mechanisms.

Cell-free massive MIMO removes cell boundaries by having a large number of distributed APs jointly serve users via coherent transmission and reception; there is no single cell center, and each user is served by a subset of nearby APs. This topology is a natural fit for ISAC: multiple nodes can illuminate and observe the same region (e.g., a road segment or intersection), and the combined aperture improves both communication diversity and sensing resolution [52]. Zhong et al. study UAV-enabled cell-free ISAC MIMO systems: multiple UAVs act as APs, each equipped with a vertical ULA, and jointly serve ground UEs while performing sensing [135]. They use an EKF to track mobile UEs from ISAC echoes; the estimated positions then drive beamforming and UAV trajectory optimization. The problem of maximizing average sum rate under sensing and power constraints is decomposed into subproblems and solved by BCD and fractional programming (FP). The combination of cell-free coordination and UAV mobility improves both sum rate and sensing accuracy compared with non-cooperative or fixed-AP setups. Cell-free and distributed ISAC deployments, increasingly relevant for intersection and urban V2X scenarios, extend the cooperative fusion concepts in Figure 13.

Singh et al. focus on target detection for an OTFS-aided cell-free MIMO ISAC system in high-mobility vehicular environments [52]. OTFS multiplexes symbols in the delay-Doppler domain, making the effective channel roughly time-invariant and thus suitable for doubly dispersive vehicular channels [136,137,138]. Multiple transmit and receive APs are connected to a C-RAN; they jointly serve UEs and detect a target using integrated communication and sensing signals. A sensing-centric (SC) approach is proposed: power allocation is optimized to maximize the sensing SNR (and thus detection probability) subject to per-UE QoS and per-AP power constraints. Regularized zero-forcing (RZF) precoding is used for communication and nullspace beamforming for the sensing stream to limit interference. The SC design is compared with a communication-centric design that minimizes total AP power; the SC scheme achieves much higher target detection probability (e.g., probability 1 at −20 dBsm RCS variance in the reported setup) while still meeting communication QoS [52]. This highlights the value of cell-free ISAC with appropriate waveform (OTFS) and sensing-centric resource allocation for V2X.

Abd et al. introduce Hydra-RAN, a dual-functional communication and sensing network architecture that can be interpreted in a distributed or cell-free spirit: perceptual networks with sensing-capable nodes are coordinated for both data and sensing services [5]. Such architectures align with 6G visions where BSs and RSUs become multi-functional nodes. The cell-free and distributed ISAC literature thus provides a path from single-cell ISAC to network-scale sensing and communication with shared or dedicated fronthaul/backhaul, as reviewed next.

In cell-free ISAC with *M* APs and *K* UEs, the downlink signal at UE *k* can be written as $y_{k}=\sum_{m=1}^{M}h_{mk}^{H}x_{m}+n_{k}$, where $x_{m}$ is the transmit vector at AP *m* and $h_{mk}$ the channel from AP *m* to UE *k*. When APs also illuminate a target, the echo at a dedicated RX or at the APs (in monostatic/bistatic fashion) depends on the same $x_{m}$ and the target channel. Joint optimization of $\left\{x_{m}\right\}$ for both sum rate and sensing SNR (or CRLB) under per-AP power constraints is a typical formulation; BCD, FP, and SDR are common solution tools [52,135]. In the OTFS-based cell-free setup, the delay-Doppler domain channel is approximately invariant over the frame, which simplifies equalization and also benefits sensing: the same precoders and power allocation can be designed over the delay-Doppler grid to maximize sensing SNR at the target location while guaranteeing minimum rates for UEs. The sensing-centric formulation in [52] shows that explicitly maximizing sensing SNR (rather than minimizing total power subject to a sensing constraint) leads to significantly better detection probability in high-mobility scenarios, which is relevant for V2X where targets (vehicles, pedestrians) are fast-moving. Representative networked ISAC architectures are compared in Table 8.

### 6.3. Fronthaul/Backhaul Constraints and Compression

In C-RAN and cell-free deployments, the central processor (CP) is connected to BSs/APs via capacity-limited fronthaul links. Transmitting full baseband signals would exceed typical fronthaul capacity, so compression and quantization are necessary. For networked ISAC, both downlink (CP-to-BS) and uplink (BS-to-CP for echoes or fused data) flows are constrained. The downlink carries the intended transmit signals (possibly compressed) to each BS; the uplink carries received echoes or preprocessed sensing data from the BSs or a dedicated sensing RX to the CP for fusion and target estimation. Capacity limits on these links directly affect the achievable communication rates and sensing accuracy, so joint design of compression and transmit/receive strategies is critical.

Zhang et al. study joint fronthaul compression and power allocation (J-FCPA) for networked ISAC [60]. The system has a CP, multiple ISAC TXs, one sensing RX, multiple users, and one target. The CP sends compressed signals to the TXs over the downlink fronthaul; the RX compresses the received echo and sends it to the CP over the uplink fronthaul. The achievable rate on the downlink fronthaul for TX *m* is $R_{m}^{\mathrm{dl}}=B\log\left(1+p_{m}/p_{m}^{\mathrm{dl}}\right)$, where $p_{m}$ is the power allocated to user *m* and $p_{m}^{\mathrm{dl}}$ is the downlink compression noise variance; similarly, the uplink fronthaul rate depends on the total received power at the RX and the uplink compression noise. The communication performance is measured by the signal-to-interference-compression-noise ratio (SICNR) at each user; the sensing performance is measured by the CRLB of the target location estimate, which is derived as a function of transmit powers and compression noise levels. The problem is to minimize total transmit power subject to fronthaul capacity limits, minimum SICNR per user, and a maximum CRLB for sensing. By analyzing the optimal compression noise levels in closed form, the number of variables and constraints is reduced; the CRLB constraint is shown to have a difference-of-convex (DC) form, and an SCA algorithm is developed. When compared to benchmarks that do not simultaneously optimize compression and power, simulations demonstrate that the suggested J-FCPA reduces power. This study is directly applicable to networked ISAC-V2X, in which scattered antennas or RSUs are connected to a central unit via fronthaul lines. In reality, the fronthaul can be either fiber or wireless (such as microwave or millimeter-wave). Since wireless fronthaul usually has higher capacity restrictions and delay variance, compression and selective forwarding—such as only when detection confidence surpasses a threshold—are particularly crucial. The fronthaul topology and the latency budget for fusion and feedback are both impacted by the option of whether the central unit for V2X is collocated with an RSU or with an aggregation node that serves several RSUs.

The trade-off between fronthaul usage and sensing quality is also discussed in the context of cooperative clustering and CRLB scaling and related cell-free ISAC references. For V2X, the choice of fusion level (data vs. symbol vs. signal) and compression strategy must account for latency and reliability requirements of safety messages and real-time sensing. The CRLB for target location in the networked setup depends on the FIM. With compressed uplink observation $\bar{r}\left(t\right)=r\left(t\right)+e^{\mathrm{ul}}\left(t\right)$ and variance $p^{\mathrm{ul}}$ for the compression noise, the FIM entries depend on $\sigma_{z}^{2}+p^{\mathrm{ul}}$ and on the effective powers $|g_{m}{|}^{2}\left(p_{m}+p_{m}^{\mathrm{dl}}\right)$ from each TX *m*. Minimizing transmit power subject to ${\mathrm{CRLB}}_{z}\leq \eta$ and $R_{m}^{\mathrm{dl}}\leq C_{m}^{\mathrm{dl}}$, $R^{\mathrm{ul}}\leq C^{\mathrm{ul}}$ leads to the J-FCPA problem; the DC structure of the CRLB constraint allows SCA to converge to a stationary point [60].

### 6.4. Edge/Cloud Integration for ISAC-V2X

**Figure 14:**
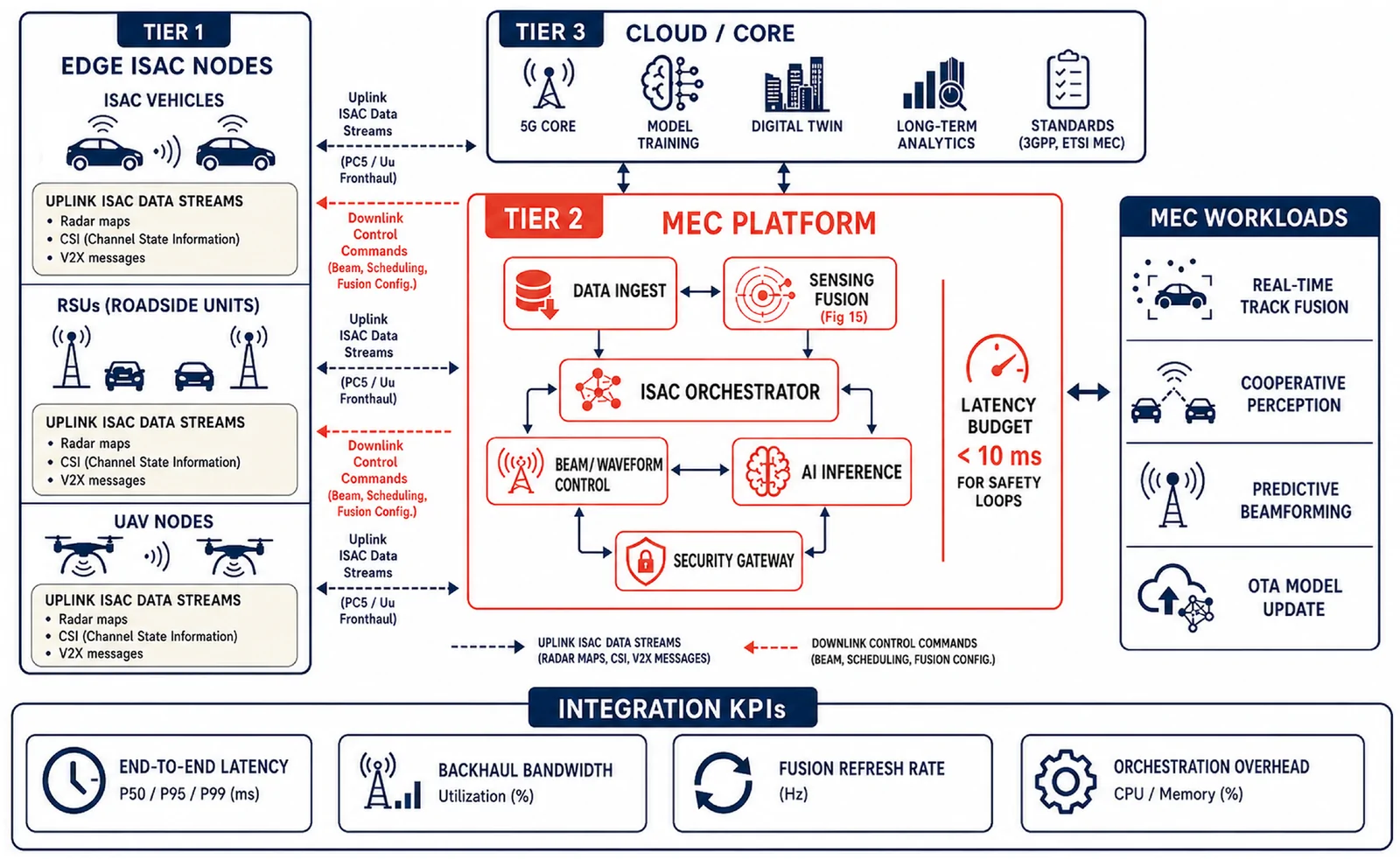
Networked ISAC and MEC integration for ISAC-V2X, illustrating edge-assisted sensing fusion, resource orchestration, and low-latency processing.

MEC brings computation and storage close to the radio access network, reducing latency for time-sensitive applications. For ISAC-V2X, MEC can host fusion of multi-BS sensing data, run tracking and prediction algorithms, and support low-latency resource allocation and handover decisions. Deploying fusion and detection at the edge avoids sending raw or symbol-level sensing streams to the cloud for every frame, which would consume backhaul and add delay; instead, only fused tracks, alerts, or aggregated statistics may be sent to higher tiers. At the same time, latency-sensitive safety applications (e.g., collision warning, cooperative perception) benefit from edge processing so that feedback to vehicles and RSUs stays within the required tens of milliseconds [30,139]. Figure 14 summarizes a typical MEC-assisted ISAC-V2X resource orchestration flow, including edge fusion and near-real-time decision feedback.

Qi et al. present a framework that integrates sensing, computing, and communication in 6G wireless networks [139]. They discuss joint optimization of resource allocation and task offloading so that sensing data can be processed at the edge with minimal delay while communication resources are shared between data delivery and sensing feedback. Wen et al. survey integrated sensing, communication, and computation, including edge and cloud roles for processing sensed data and coordinating multi-node ISAC [30]. These works position MEC as a natural place to aggregate ISAC outputs and run control and safety applications.

Shen et al. propose a cloud–edge cluster collaboration architecture for the IoV with software-defined networking (SDN) [140]. Multiple edge service providers (ESPs) contribute edge resources to a shared pool; the cloud supplements when edge capacity is insufficient. A dynamic pricing model encourages ESPs to participate. Task offloading and computing resource allocation are formulated to minimize task latency and maximize ESP profit; a clustering step (e.g., K-means++) determines initial offloading nodes, and a Double Deep Q-Network (DDQN) is used to learn resource allocation. Although not ISAC-specific, the architecture is relevant when ISAC processing (e.g., fusion, detection) is offloaded to edge or cloud in V2X.

Gong et al. combine digital twin (DT), network slicing, and ISAC for the IoV [111]. They propose an environment-aware offloading mechanism (EAOM) that uses ISAC to obtain environment and mobility information; task scheduling and resource allocation are jointly optimized to minimize overall response time (ORT). The problem is modeled as a Markov decision process and solved with an algorithm combining Shapley Q-value and DDPG. The ISAC-assisted mechanism provides two transmission paths (e.g., direct and sensing-assisted), shortening ORT compared with traditional offloading. This illustrates how ISAC and MEC can be coupled for V2X resource and offloading decisions.

Liu et al. study energy-efficient computation offloading in a UAV-aided V2X network with ISAC [141]. UAVs equipped with sensors and MEC capabilities assist vehicles in environmental perception; sensing data from multiple vehicles are fused at the UAV, and part of the fusion workload can be offloaded to ground RSUs. The problem minimizes a weighted sum of UAV energy consumption and user latency by optimizing offloading decisions, bandwidth, and computational resource allocation. SCA is used to handle the non-convex formulation. The work shows how MEC and ISAC can be integrated in a vehicle–UAV cooperative perception architecture for V2X. Feng et al. and Nemati et al. discuss joint communication, sensing, and computation in 6G and IoT contexts, reinforcing the role of edge/cloud for processing and coordination in ISAC systems [24,142].

A generic MEC-ISAC integration objective is to minimize the overall response time $T_{\mathrm{ort}}=T_{\mathrm{ul}}+T_{\mathrm{queue}}+T_{\mathrm{comp}}+T_{\mathrm{dl}}$, where $T_{\mathrm{ul}}$ is uplink transmission time (e.g., for sensed data or offloaded tasks), $T_{\mathrm{queue}}$ is queuing delay at the edge server, $T_{\mathrm{comp}}$ is computation time, and $T_{\mathrm{dl}}$ is downlink feedback time. When ISAC provides environment and mobility information, the allocator can choose offloading targets and resource splits as a function of vehicle position and channel state, reducing $T_{\mathrm{ul}}$ and $T_{\mathrm{queue}}$ through better placement and scheduling.

### 6.5. Joint Resource Allocation (Spectrum, Power, Time)

Joint resource allocation in networked ISAC-V2X must balance communication rate, sensing performance (e.g., SNR, CRLB, detection probability), and possibly latency and energy under power, bandwidth, and fronthaul constraints. The decision variables typically include per-node or per-user power (or power spectral density), beamforming vectors or covariance matrices, time and frequency slot allocation, and in some cases compression parameters or offloading decisions. Constraints include total or per-antenna power, per-user minimum rate or SINR, sensing quality (e.g., minimum SNR at the target, maximum CRLB for location), fronthaul capacity, and latency or queue stability. Because the sensing and communication objectives are often conflicting (e.g., directing power toward the target improves sensing but may increase interference to some users), Pareto-optimal or weighted-sum formulations are common, and solution methods range from convex approximations (SDR, SCA, FP) to learning-based policies (DRL, DDQN) when the problem is non-convex or the environment is time-varying [111,140].

Yang et al. consider queue-aware dynamic resource allocation for a joint communication-radar system [143]. The system shares time and spectrum between communication and radar; the objective is to optimize a utility that depends on communication throughput and radar performance (e.g., detection or estimation quality) while respecting queue stability. The formulation captures the temporal coupling of traffic and sensing demands and is solved with a Lyapunov-based approach. This type of framework extends to multi-BS or cell-free ISAC when each node has a queue and shared time-frequency resources.

Wei (Chun-Yi) addresses low-latency ISAC optimization for 6G V2X [77]. Safety-critical V2X requires very low sensing latency (e.g., 3GPP targets on the order of 20–50 ms). Beam misalignment, precoder imperfections, and target RCS fluctuations (modeled with Swerling statistics) increase the number of sensing repetitions or block length needed to achieve a target detection probability. The article derives detection thresholds and repetition bounds and formulates a mixed-integer non-linear program (MINLP) to minimize sensing latency subject to detection probability and communication SINR constraints. A branch-and-bound (B&B) algorithm is proposed and shown to achieve low complexity (e.g., 0.11 ms) and latency below 15 ms in simulations, meeting 3GPP-style V2X latency targets. This highlights that resource allocation in ISAC-V2X must explicitly account for latency and reliability, not only rate and SNR.

Annu and Rajalakshmi survey resource allocation for 6G V2X sidelink, including mode selection (Mode A/B), power-based and time-frequency allocation, interUE coordination, and emerging techniques such as ML-aided and edge-computing-driven allocation [13]. They outline mathematical formulations and challenges for V2V, V2I, and V2P services. Although the survey is not ISAC-specific, the same resource dimensions (power, time, frequency, space) and coordination mechanisms apply when sidelink carries both communication and sensing-related signaling or when ISAC-capable RSUs allocate resources for vehicular users.

Wu et al. study resource allocation in heterogeneously distributed joint radar–communications under asynchronous Bayesian learning, showing how distributed nodes can learn and adapt resource allocation with imperfect synchronization [144]. The formulation is relevant to multi-BS ISAC where nodes have different capabilities and synchronization is not ideal. Gong et al. and Singh et al. (as cited above) provide further examples of power and beamforming allocation for ISAC under communication and sensing constraints in IoV and cell-free settings [52,111]. A typical joint resource allocation problem maximizes a weighted sum of communication utility (e.g., sum of user rates $R_{k}$) and a sensing metric S over beamforming matrices W, power or time splits ρ, and possibly repetition or block length τ, subject to total power, per-user minimum rate, minimum sensing quality, fronthaul, and latency constraints; the weights and constraints are scenario-dependent and are instantiated in the references above for multi-BS, cell-free, and V2X cases.

In the low-latency formulation of [77], the number of sensing repetitions $N_{\mathrm{rep}}$ and the block length *L* determine the sensing latency $T_{\mathrm{sens}}\propto N_{\mathrm{rep}}\cdot L$. For a target detection probability $P_{d}\geq P_{d}^{\min}$ and false-alarm probability $P_{\mathrm{fa}}\leq P_{\mathrm{fa}}^{\max}$, the minimum $N_{\mathrm{rep}}$ is derived from Swerling target models and beam misalignment; the optimizer then chooses $\left(N_{\mathrm{rep}},L\right)$ and the detection threshold to minimize $T_{\mathrm{sens}}$ while satisfying communication SINR.

The V2X sidelink and 6G resource allocation literature presented in Table 9 discusses how dynamic spectrum access and cognitive-radio-inspired techniques can enable ISAC nodes to opportunistically exploit idle bands for sensing in shared bands.This table demonstrates how resource allocation in networked ISAC-V2X goes well beyond traditional spectrum and power management. Depending on the architecture, other optimization variables include trajectories, computation offloading, compression, sensor repetitions, and processing resources. Cross-layer and scalable optimization techniques that jointly account for communication, sensing, computing, and mobility are motivated by this diversity.

### 6.6. Mobility Management and Handover in ISAC-V2X

Vehicles move at high speed, so cell association, handover, and beam tracking must be fast and reliable. ISAC can support mobility management by providing real-time position, velocity, and channel-related information from the same waveform used for communication [48,67].

Liu et al. propose learning-based predictive beamforming for ISAC in vehicular networks [48]. Instead of reactive beam updates after channel change, the BS uses ISAC echoes to estimate or predict vehicle position and velocity and then predicts the best beam for the next slot. This reduces signaling overhead and latency and improves robustness to high mobility. The idea extends to multi-BS: predictive handover can use fused sensing from several BSs to decide when and to which cell to hand over.

Yuan et al. study ISAC-assisted OTFS transmission for vehicular networks [67]. OTFS is robust to Doppler and delay spread; combined with ISAC, the BS can estimate the vehicular channel (and thus mobility) from the same OTFS frame used for data. The estimated channel or mobility state can drive resource allocation and handover decisions. This fits the trend of using ISAC-derived state for mobility management in V2X.

Jiang et al. consider UAV-enabled ISAC with tracking design and optimization: the UAV trajectory and sensing/communication parameters are jointly optimized so that the UAV can track the target (e.g., a vehicle or another UAV) while maintaining communication links [81]. The formulation can be adapted to ground BSs or RSUs that track vehicles and adjust association and beams accordingly. For multi-UAV-assisted ISAC, Zhang et al. address joint UAV trajectory, user association, and beamforming, which once more links trajectory and association to sensing and communication performance [145]. The analog for terrestrial V2X is the selection and handover of RSU/BS based on position determined by ISAC and expected link quality.

Low-latency ISAC optimization [77] and cooperative multi-BS frameworks [59,130] also support mobility. Lower sensing latency means faster updates of the vehicle state for handover and beam prediction, and multi-BS fusion improves state estimation accuracy across coverage boundaries. Standardization and system design for ISAC-V2X will need to specify how sensing outputs (position, velocity, uncertainty) are used for handover and mobility management in 3GPP and IEEE stacks [3].

Predictive handover can be cast as follows: at time *n*, using ISAC-derived state ${\hat{s}}_{k}\left[n\right]$ (position, velocity) for vehicle *k*, predict the best serving cell and beam at time n+Δ. The predictor can be model-based (e.g., constant velocity model with EKF-updated state) or learning-based (e.g., recurrent or transformer models trained on past trajectories). The same state is useful for beam prediction and for resource reservation at the target cell, reducing handover failure and latency. Integration with 3GPP handover procedures (e.g., measurement reporting, conditional handover) and with sidelink resource allocation (Mode A/B) remains an open area for ISAC-V2X [13,48]. 3GPP has defined latency targets for V2X services (e.g., end-to-end latency on the order of 20–50 ms for safety messages and for sensing-based applications); meeting these in a networked ISAC setting requires not only low-latency sensing but also fast fusion at the edge and timely delivery of sensing-aided decisions (e.g., handover command or beam switch) to the vehicle. Mobility management in ISAC-V2X thus couples physical-layer sensing, edge/cloud processing, and higher-layer protocols and will benefit from cross-layer design and standardization that explicitly incorporate sensing outputs into mobility and resource procedures.

**Research directions.** Key open challenges for networked ISAC-V2X include scalable fusion and resource-allocation algorithms for large numbers of BSs, RSUs, and users under heterogeneous fronthaul and stringent latency constraints; integration of ISAC-derived mobility information with standardized handover and sidelink procedures; and experimental validation of multi-BS cooperative sensing and cell-free ISAC under realistic synchronization and channel conditions. Further research is also needed on joint communication, sensing, and computation offloading across edge and cloud infrastructures. Table 8 and Table 9 summarize the corresponding architecture and resource-allocation perspectives.

### 6.7. Summary and Transition to Section 7

Section 6 showed that networked ISAC-V2X extends joint sensing and communication from individual links to coordinated multi-node systems. Multi-BS/RSU cooperation, cell-free and distributed architectures, fronthaul/backhaul management, edge/cloud processing, joint resource allocation, and mobility-aware operation can improve coverage and sensing performance but introduce additional synchronization, signaling, latency, and scalability challenges. Their practical realization therefore requires coordinated optimization of waveform design, beamforming, fusion, spectrum, power, and network resources under vehicular mobility and reliability constraints.

These findings directly motivate G1 and G5: although the reviewed studies demonstrate the benefits of multi-node cooperation, cell-free architectures, and joint resource allocation, unified sensing–communication performance metrics and scalable cooperative optimization under high vehicular mobility remain insufficiently developed. In addition, the limited experimental validation of multi-BS and cell-free ISAC architectures under realistic synchronization and channel conditions contributes directly to G4.

Section 7 builds on this networked foundation by considering multi-modal and AI-native ISAC-V2X, where radar, LiDAR, camera, and communication-derived sensing information are combined with AI/ML for prediction, beam management, target processing, and semantic communication.

## 7. Multi-Modal and AI/ML-Enabled ISAC-V2X

This section reviews the convergence of multi-modal sensing, AI, and ML with ISAC for V2X networks. As 6G and beyond move toward native intelligence and richer environmental perception, the combination of radar, LiDAR, camera, and RF sensing with data-driven algorithms is becoming central to both sensing-assisted communication and communication-assisted sensing. The section is organized into six subsections: multi-modal sensing fusion (Section 7.1), AI/ML for channel prediction and beam management (Section 7.2), DL for target detection and tracking (Section 7.3), FL for privacy-preserving ISAC (Section 7.4), generative AI for ISAC (Section 7.5), and semantic communication in ISAC-V2X (Section 7.6).

### 7.1. Multi-Modal Sensing Fusion (Radar, LiDAR, Camera, RF)

Multi-modal ISAC-V2X extends RF-only designs by combining radar, LiDAR, camera, RF, and positioning information to support channel prediction, beam management, blockage prediction, tracking, and cooperative perception [5,8,9]. These modalities provide complementary capabilities: radar and RF sensing offer range, velocity, and angle information, LiDAR provides high-resolution geometry, cameras add semantic information, and GNSS/IMU support mobility awareness. Their fusion can be implemented at vehicle, infrastructure, or hybrid levels, with trade-offs among accuracy, latency, backhaul load, hardware cost, and privacy.

**Figure 15:**
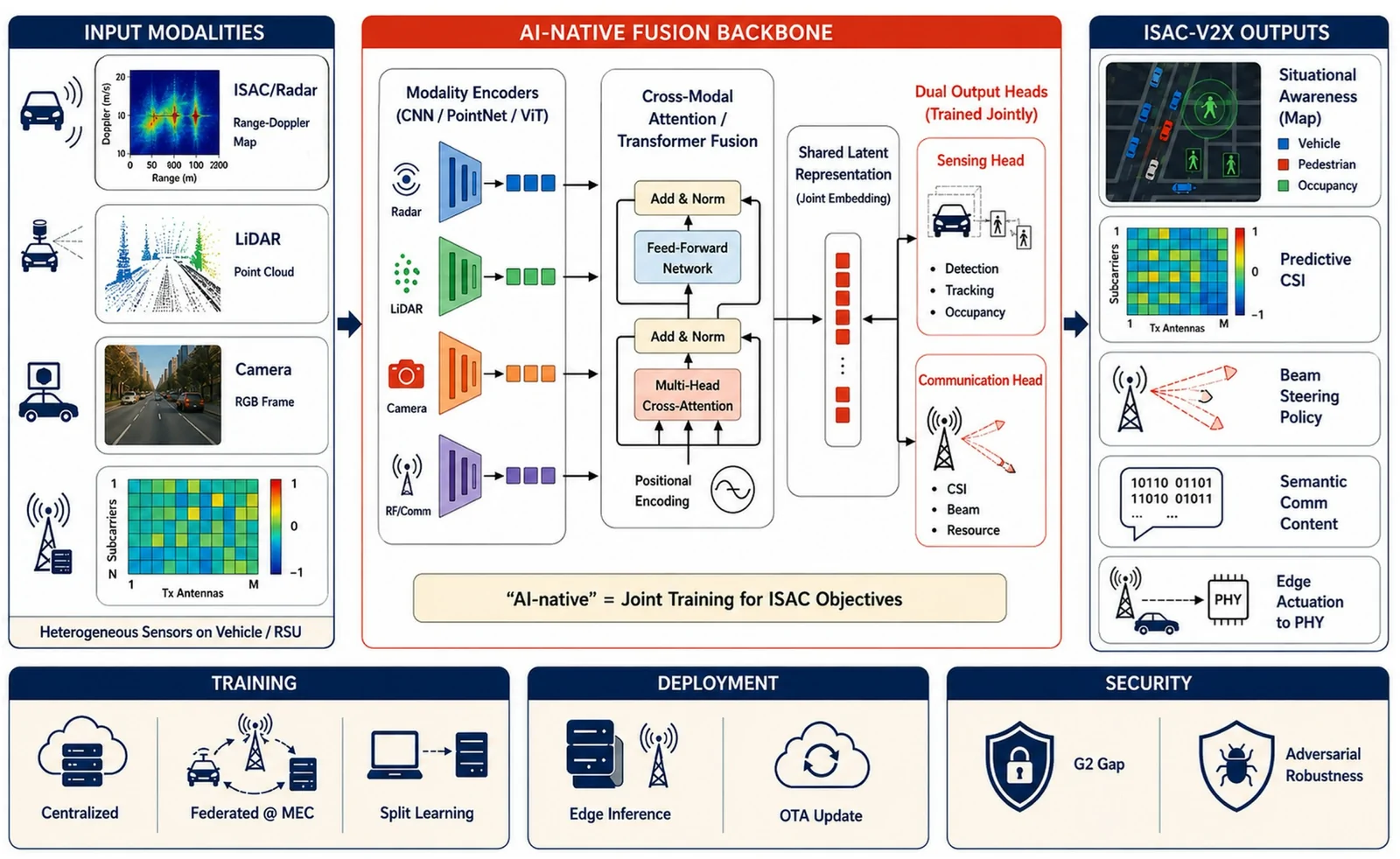
Multi-modal AI-native ISAC-V2X architecture integrating heterogeneous sensing and communication modalities through cross-modal fusion and shared representation learning.

Figure 15 illustrates a representative multi-modal fusion pipeline that supports both sensing-assisted communication and communication-assisted sensing.

Abd et al. propose the Hydra-RAN (H-RAN) perceptual network architecture, which merges communication and sensing within a unified framework and employs multi-sensor data fusion and AI/ML for real-time decision-making, beam management, blockage avoidance, and network self-organization [5]. In contrast, Wang et al. provide a broader system-level perspective on the coexistence and integration of communication and radar sensing, covering enabling techniques, applications, tools, and datasets [9]. Together, these perspectives illustrate two complementary directions in multi-modal ISAC: architecture-level fusion of distributed sensing information and system-level integration of communication and sensing resources.

At the data level, multi-modal fusion can be performed at raw, feature, decision, or hybrid stages, depending on where heterogeneous sensing and communication information is combined. Existing V2X-oriented studies exploit combinations of GNSS, RGB images, LiDAR, radar, sub-6 GHz CSI, and depth information for tasks such as beam prediction, channel estimation, blockage prediction, and cooperative perception [8]. Raw-level fusion preserves the richest information but increases communication and processing overhead, whereas feature-level fusion offers a more compact representation at the cost of additional local processing. Decision-level fusion reduces backhaul demand and supports distributed operation, although some cross-modal information may be lost. Hybrid approaches seek a balance among these alternatives. Therefore, the preferred fusion level depends on prediction accuracy requirements, latency, backhaul capacity, edge-computing resources, privacy constraints, and vehicular mobility.

For data-centric multi-modal ISAC-V2X, datasets and simulators are essential. Ray-tracing and 3D simulation can supplement limited real-world measurements, while DeepSense 6G, Raymobtime, and ViWi offer representative multi-modal resources for channel and beam prediction [8,14]. However, in addition to spectrum availability, roadside hardware cost, privacy, and the difference between synthetic and actual situations, the lack of large-scale synchronized RF, radar, LiDAR, camera, and positional data continues to be a significant impediment for training and evaluation [3]. The associated multi-modal fusion taxonomy is summarized by fusion level, modality, and typical use case in Table 10.

### 7.2. AI/ML for Channel Prediction and Beam Management

In ISAC and V2X systems, where model-based optimization is frequently too complicated or sluggish for real-time operation, AI and ML are being employed more and more for channel prediction, beam management, and resource allocation. A mapping from previous CSI or sensing features to future CSI or beam indices can be learned by channel prediction; the loss is then either cross-entropy for classification or MSE for regression. Prediction horizons and update rates must be carefully selected to balance overhead and performance in high-mobility V2X due to the short coherence time.

More generally, supervised, unsupervised, reinforcement, transfer, federated, and generative learning techniques for waveform design, beamforming, channel estimation, sensing, and resource management are all included in AI-enabled ISAC [25,49]. The dimensionality of the decision space, the latency and privacy limitations of the vehicular application, the availability of labeled data, and the requirement for online adaptation all influence their usefulness.

**Figure 16:**
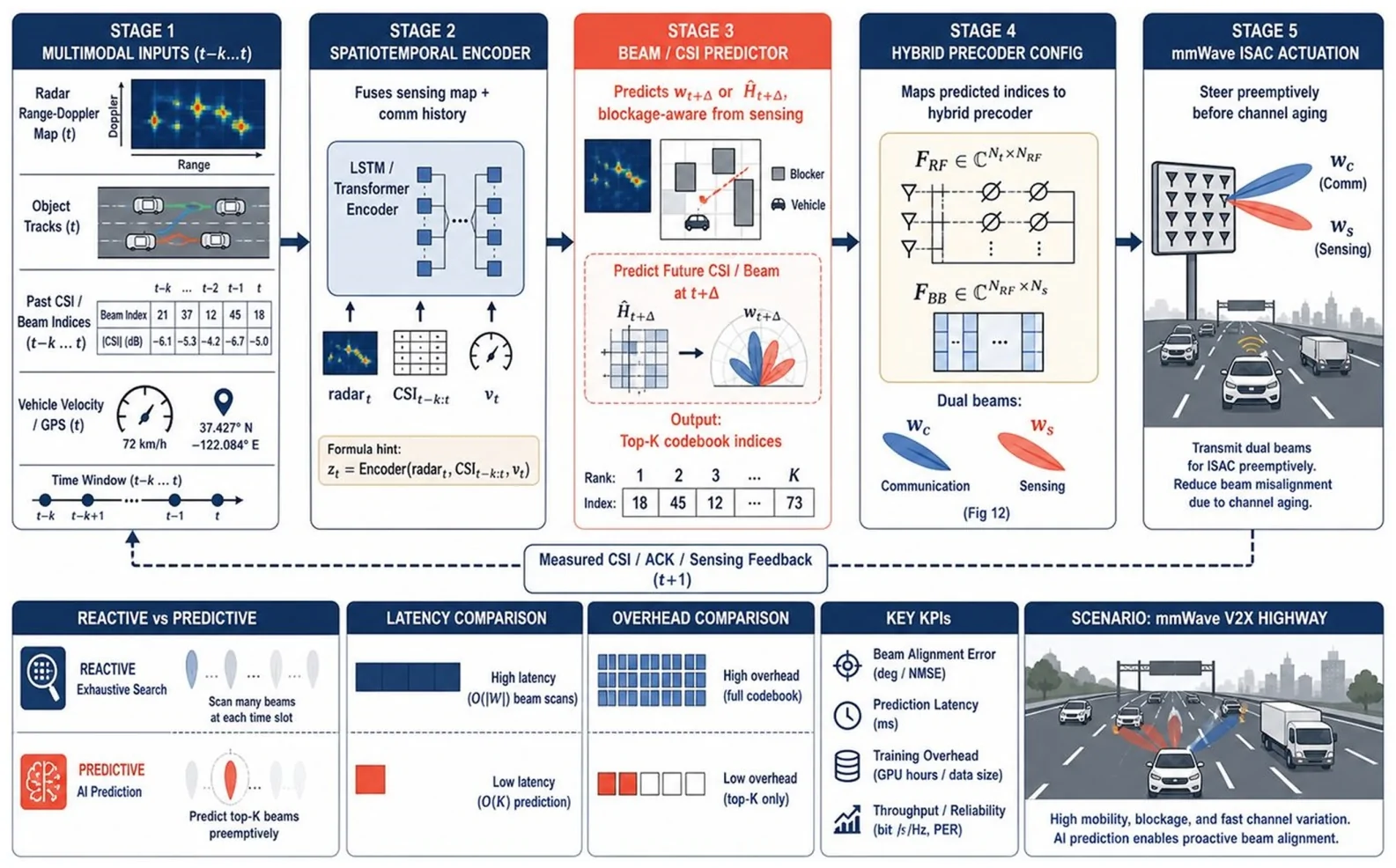
Multi-modal beam prediction pipeline for mmWave ISAC-V2X, illustrating sensing-assisted prediction and proactive beam management.

One of the most developed AI applications in vehicle ISAC is predictive beam management. MLPs, CNNs, and recurrent architectures are examples of supervised deep models that learn mappings from past CSI, mobility states, radar range-angle or Doppler representations, and other sensing characteristics to future beams or beamforming parameters. These techniques can minimize explicit beam-search and channel-sounding overhead by utilizing sensing information prior to link degradation, and they are especially appealing for quickly changing mmWave V2X links [6,48,146]. The need for representative training data, sensitivity to changes in the distribution of the environment, and the requirement that inference and model updates stay faster than the underlying channel dynamics are the primary practical constraints. The multi-modal beam prediction pipeline for mmWave ISAC-V2X covered in this subsection is shown in Figure 16. The choice of learning paradigm is strongly task-dependent. Supervised learning is effective for channel estimation and beam prediction when labeled data are available, whereas unsupervised and semi-supervised approaches are useful when labels are limited or when physical performance objectives can be embedded directly into the training loss. Transfer learning can reduce retraining requirements when vehicles, propagation environments, or sensing conditions change, while distributed learning provides a natural framework for exploiting data collected across multiple vehicles and infrastructure nodes. FL is discussed in greater detail in Section 7.4 [25,61].

Reinforcement learning (RL) and DRL do not require labeled data but instead learn from rewards; the objective is often formulated as maximizing the expected discounted reward over time, e.g., $J_{T}\left(i_{0}\right)=E\left[\sum_{t=0}^{T}\alpha_{d}^{t}R\left(x_{t}\right)|x_{0}=i_{0}\right]$, with discount factor $\alpha_{d}$ and reward R(·), which can represent sum rate, sensing accuracy, or a weighted combination [61]. DRL is well suited to sequential decision-making in ISAC, including beam and resource allocation, trajectory design, and waveform adaptation.

From a vehicular perspective, RL/DRL is particularly attractive when optimization variables evolve over time and depend on previous system states, such as vehicle trajectories, beam configurations, RIS phases, or dynamic resource allocation. Recent learning-based ISAC studies have therefore applied RL/DRL to resource allocation, trajectory control, RIS configuration, beamforming, and secure sensing–communication optimization [119,147,148]. These approaches are especially useful when conventional optimization must be repeatedly solved under rapidly changing channel, mobility, or network conditions. However, training costs, convergence behavior, policy stability, and the requirement to ensure safe decisions in latency-critical traffic conditions continue to limit their practical application.

Overall, there is a distinct separation of roles in the AI/ML literature for beam management and channel prediction. Supervised models are primarily suited to prediction tasks, RL is more naturally aligned with sequential resource and control decisions, and transfer and distributed learning approaches address adaptation and data-sharing constraints. Accordingly, the practical choice of learning paradigm should be determined not by model architecture alone but by data availability, mobility-induced distribution shifts, online adaptation requirements, inference latency, communication overhead, privacy, and safety-critical robustness.

### 7.3. Deep Learning for Target Detection and Tracking

DL is widely applied to target detection, parameter estimation, and tracking in ISAC and dual-function radar–communication systems. Wild et al. note that DL has been used for static object classification, radar-based fall detection, semantic segmentation of radar point clouds, and detection and localization of multiple object classes in a single frame; they also mention GAN-based methods for ghost-target removal in range-Doppler estimation [35]. Temiz et al. categorize DL-based ISAC techniques for sensing: CNNs for target detection, parameter estimation, and recognition from radar imagery; RNNs/LSTMs for temporal tracking; and autoencoders or GANs for denoising and data augmentation [61].

Yin et al. propose a DL-assisted ISAC framework for UAV detection in multi-path environments using micro-Doppler. They model per-UAV micro-Doppler signatures (e.g., interpropeller angle offsets), use DL to associate multi-paths belonging to the same UAV via signature clustering, and report a large reduction in false alarms with OFDM as the ISAC waveform; the framework is the first to employ DL to identify individual UAVs of the same category (same number of propellers and size) from subtle micro-Doppler differences [149]. Xie et al. study RIS-aided radar for target detection with clutter region analysis and joint active–passive design, which can be combined with learning-based detection and waveform optimization [123]. Singh et al. address target detection for OTFS-based cell-free MIMO ISAC, where multiple APs cooperate for both communication and sensing [52]. In order to improve resolution and detection reliability in spectrum-efficient setups, Shah et al. suggest Newtonized Orthogonal Matching Pursuit for high-resolution target detection in sparse OFDM ISAC systems [75]. ISAC is taken into consideration by Zhou et al. in UAV swarms for cooperative multiple-target tracking, where learning can support state estimation and data association across multiple UAVs [85]. Ahmed and Cho examine machine learning for healthcare radars (activity recognition, vital signs), which has methodological similarities to machine learning for radar-based detection and classification in ISAC-V2X [150].

It is possible to formulate dual-function waveform design as an optimization problem and then substitute or approximate it with learnt mappings. Temiz et al. provide an example of how to satisfy per-antenna power limits and minimize multi-user interference (Frobenius norm between HX and intended symbols S) while remaining near a desired radar waveform $X_{0}$ [61]. To minimize data and complexity, model-based DL (such as deep unfolding) integrates such structure into the network. For ISAC transceiver design, Zhang et al. suggest deep unfolding learning, which combines learnable parameters with iterative algorithms for reduced complexity and improved performance [97]. In order to balance sensing and communication goals, Pulkkinen and Koivunen employ model-based online learning for active ISAC waveform tuning [89]. Joint beamforming for multi-target detection and multi-user communication in ISAC, where detection and communication metrics are jointly maximized, is addressed by Zhao et al. [92]. This situation is accessible to learning solvers. Long et al. study GPS-denied ISAC vehicle localization using mmWave radar and identification, with learning for robust localization in challenging environments [151]. Wang et al. consider ISAC-enabled cooperative detection for cellular-connected UAV networks, combining detection performance with cellular connectivity in a framework where learning can improve both [133]. Zheng et al. demonstrate how large-scale pretrained or distilled models can assist sensing and identification by using RF fingerprinting and a distilled LLM for UAV individual identification in ISAC networks [152]. In summary, DL for target detection and tracking in ISAC-V2X spans CNN-based classification and detection from radar or fused inputs, RNN/LSTM-based temporal tracking and prediction, model-based DL for waveform and transceiver design, and unsupervised or semi-supervised methods when labels are scarce. For channel/beam and sensing tasks, representative machine learning techniques utilized in ISAC-V2X are compared in Table 11. It is evident from this table that no single learning paradigm works well for all ISAC-V2X jobs. While RL and FL are more appropriate for sequential decision-making and distributed privacy-sensitive situations, supervised and DL approaches work well for prediction and detection when there are enough labeled data. Therefore, the practical decision is based on the targeted sensing or communication job, robustness, inference latency, communication overhead, and data availability.

### 7.4. Federated Learning for Privacy-Preserving ISAC

ISAC systems combine data from numerous dispersed sensors and devices, as centralizing raw data presents issues with transmission costs and privacy. FL keeps raw sensor and channel data on-device while enabling nodes to train locally and share only model updates (such as gradients or weights). FL for ISAC is described by Zhu et al. as follows: sensor nodes train local models on local data then share and consolidate models through an aggregation process to obtain a global model without disclosing raw data, enhancing both privacy and usage of distributed data and compute [56]. In order to accomplish automatic detection and recognition while maintaining indoor sensing privacy and facilitating distributed edge training, Qi et al. employ federated transfer learning for device-free sensing. Zhang et al. investigate collaborative beamforming and resource allocation optimization in ISAC-accelerated edge intelligence, which can be paired with FL for distributed training.

FL is discussed by Wu et al. in relation to integrated sensing and backscatter communication (ISABC) with RIS assistance. IoT devices preprocess echo signals, train local sensing models (such as neural network weights), then upload these models via backscatter to the sensing node. The sensing node then updates a global model by aggregating local models, which it then sends back to the devices. As a result, substantially less information is communicated than in traditional distributed sensor networks, and an accurate distributed sensing model is constructed without sharing raw echo data [154]. The model is updated as a weighted sum of local model parameters using a straightforward FedAvg-style aggregation, with weights corresponding to local dataset sizes [61]. Heterogeneous data amounts or quality can be taken into consideration with weighted aggregation; secure aggregation techniques enhance privacy by enabling the server to calculate the total of updates without viewing individual updates. The size of model parameters or gradients, the number of rounds, and the percentage of nodes involved in each round all affect the communication cost of FL in ISAC-V2X, and compression and sparsification of updates can lower overhead at the expense of ultimate accuracy or convergence speed. Additionally, FL is brought up in connection with beam selection: Mashhadi et al. employ FedAvg for model aggregation and FL for cooperative beam selection with LiDAR and BS location, with the BS acting as the aggregator [8]. Federated and distributed learning difficulties are reviewed by Noman et al. for upcoming wireless networks in 6G [129]. For 6G intelligent machine systems, Feng et al. address joint communication, sensing, and computing, where FL can facilitate distributed learning across sensing and communication resources [24]. The sensing-assisted communication in vehicular networks with intelligent surfaces is reviewed by Meng et al. [156]. Distributed or federated training can use data from several vehicles and RSUs without centralizing raw sensor data. In ISAC deployments, latency and communication costs are strongly impacted by the trade-off between aggregation frequency, local epochs, and convergence in FL; robust aggregation is still a hot topic when sensing nodes are heterogeneous or unreliable. In conclusion, FL makes it possible to train sensing and communication models across dispersed ISAC nodes while maintaining privacy. Global model updates are based on the FedAvg aggregation rule and its variations; integration with differential privacy and over-the-air computation for additional privacy guarantees is an ongoing research direction. The impact of non-IID data between vehicles or RSUs and the privacy–utility trade-off are crucial factors for ISAC-V2X FL deployment. Differential privacy can be applied to local updates (e.g., clipping and noising gradients) so that the aggregated model does not expose individual data.

### 7.5. Generative AI for ISAC (Data Augmentation, Channel Modeling)

In ISAC and 6G, GenAI—which includes generative adversarial networks (GANs), variational autoencoders (VAEs), and diffusion models—is utilized for data augmentation, channel modeling, and the creation of synthetic data in situations when real data are costly or limited. In their study of GenAI-enabled 6G technologies and their role in digital twins, RIS, UAV, and ISAC, Sheraz et al. emphasize that GenAI can support scenario modeling and adaptive learning while addressing data scarcity [29]. Wild et al. note that GANs can help with error propagation and ghost targets in range-Doppler estimation (e.g., GAN-CRT) and that generative models could help simulate channels for joint communication and sensing when ray-tracing or measurements are limited [35]. Shafique and Alhassoun suggest GANs to generate virtual RIS channels and reduce CSI acquisition frequency and FL at the edge for privacy [155]. Zhu et al. mention adversarial generative networks as a threat (e.g., fooling AI-based detection) and cite works on WGAN-GP for beamforming inference and GAN-based wideband channel estimation [56].

Dassanayake et al. design unsupervised learning-based ISAC waveforms, leveraging generative or representation learning without labeled data [153]. Zhang et al. survey intelligent ISAC and list GenAI among the key AI techniques for waveform design, beamforming, resource allocation, and parameter estimation in RIS, edge, UAV, and V2X-ISAC settings [25]. Beyond GANs, diffusion models and VAEs are increasingly used for channel and signal generation: diffusion models can produce high-fidelity samples by iteratively denoising, and VAEs learn latent representations that can be sampled for data augmentation or channel imputation. Sheraz et al. discuss generative diffusion models and their applications for optimizing network performance in 6G, which can be extended to ISAC channel and scenario synthesis [29]. Concrete use cases for GenAI in ISAC-V2X include the following: generating additional training samples for beam or detection models when real data are limited; imputing missing channel or sensor measurements; simulating rare or dangerous scenarios (e.g., extreme weather, congestion) for testing; and learning compact latent representations of channels or scenes for semantic or compressed feedback. Evaluation of generative models in this context should report both fidelity (e.g., distribution match or downstream task performance when training on generated data) and diversity to avoid overfitting to a narrow subset of channels or scenarios. In line with data-driven and generative modeling for channels and scenarios [49], Merluzzi et al. include AI/ML and future sensing/communication integration in the Hexa-X roadmap. Generative models can support synthetic datasets for training and channel/signal creation, according to Wen et al.’s assessment of integrated sensing, communication, and computation [30]. Thus, applications of GenAI in ISAC-V2X include secure semantic or incentive mechanisms, channel and scenario generation for simulation, and data augmentation for detection/beam prediction [56]. Ensuring that produced channels or scenarios maintain physical consistency and that GAN-based systems are resilient to mode collapse and adversarial inputs when employed in closed-loop ISAC control are among the challenges. Additionally, the requirement for verification and monitoring when implementing generative models in safety-critical V2X applications is highlighted by misuse of GenAI.

### 7.6. Semantic Communication in ISAC-V2X

Semantic communication aims to transmit and process information at the level of meaning or task-relevant features rather than bit-level fidelity, which can reduce bandwidth and latency and align transmission with downstream tasks (e.g., detection, control). In ISAC-V2X, semantic approaches can be applied to both communication (e.g., sending sensing summaries or features instead of raw waveforms) and sensing (e.g., extracting and sharing only task-relevant semantic features).

Zhu et al. reference semantic communications for wireless sensing (e.g., RIS-aided encoding and self-supervised decoding), AI-generated incentive mechanisms and full-duplex semantic communications for information sharing, and semantic-aware sensing information transmission for the metaverse [56]. They also cite generative AI-aided joint training-free secure semantic communications with multi-modal prompts. Annu and Rajalakshmi list “Understand-Before-Talk” (UBT) and semantic communication as relevant to 6G V2X in their survey of resource allocation and future directions [13]. Cheng et al. discuss functional and signaling ISAC and “synesthesia of machines” (SoM) as a multi-modal ISAC framework that can incorporate semantic or task-oriented representations [7]. Yang et al. propose SoM-enhanced ISAC precoding for vehicular networks with double dynamics, which can be interpreted as using richer (including semantic) representations for precoding [157]. Semantic communication shifts the design goal from bit-level reliability to task success (e.g., correct detection, correct beam choice, or low control latency), which can reduce required bandwidth and latency when the receiver only needs high-level information; however, defining and measuring semantic fidelity for joint sensing and communication remains an open problem.

Integrating semantic communication with ISAC-V2X is still emerging. Potential directions include the following: (i) encoding sensing outputs (e.g., detections, tracks, or risk scores) into compact semantic messages for V2X broadcast; (ii) using semantic features from vision or LiDAR to guide beam or resource selection; and (iii) joint source-channel coding of sensing and communication messages for latency-critical safety applications. Wang et al. survey symbiotic sensing and communications toward the 6G vision, applications, and technology trends, which naturally align with semantic and task-oriented interfaces between sensing and communication [17]. Research on standardization and performance bounds for semantic ISAC is still ongoing.

In summary, Section 7 showed how multi-modal sensing and AI/ML can support prediction, fusion, detection, and resource management in ISAC-V2X. At the same time, the reviewed literature reveals persistent limitations in multi-modal integration, robustness, privacy, dataset availability, model generalization, and scalable distributed learning, which directly motivate G2, G4, and G5. Section 8 consolidates these findings with the research gaps synthesized throughout the preceding sections.

## 8. Conclusions and Future Directions

This section synthesizes the main findings of Section 1, Section 2, Section 3, Section 4, Section 5, Section 6 and Section 7 and consolidates the key research gaps, future directions, standardization and testbed recommendations, and the 2026–2030 deployment roadmap for ISAC-V2X.

### 8.1. Summary of Key Findings

Section 1, Section 2, Section 3 and Section 4 established the foundations of ISAC-V2X, covering vehicular requirements, ISAC architectures and taxonomy, high-mobility channel and sensing models, and physical-layer techniques including OFDM, OTFS, DFRC, beamforming, RIS/STAR-RIS, NOMA/RSMA, and joint resource allocation. Across these areas, the literature consistently shows that practical ISAC-V2X design is governed by coupled trade-offs among sensing accuracy, communication reliability, mobility robustness, computational complexity, and signaling overhead [1,3,27,64,85,86].

Section 2, Section 3, Section 4, Section 5, Section 6 and Section 7 extended this perspective to security, networked operation, and intelligent multi-modal processing. Security and privacy must be co-designed with sensing and communication; multi-BS/RSU and cell-free architectures improve coverage and cooperative perception but introduce fronthaul, synchronization, latency, and coordination constraints; and multi-modal AI/ML approaches enable prediction, fusion, detection, and distributed learning while raising challenges in robustness, privacy, data availability, and model generalization [8,25,26,33,59,60].

Overall, practical ISAC-V2X deployment remains constrained by high-mobility operation, scalable multi-node coordination, fronthaul and synchronization overhead, multi-modal and AI/ML robustness, security and privacy, and limited standardization and experimental validation. These interconnected challenges motivate the consolidated research gaps G1–G5 discussed next.

To make the derivation of G1–G5 from the reviewed literature explicit, Table 12 links the principal findings of Section 2, Section 3, Section 4, Section 5, Section 6 and Section 7 to the unresolved limitations from which the consolidated research gaps emerge.

### 8.2. Consolidated Research Gaps (G1–G5)

Building on the limitations synthesized in Section 1 and refined across Section 2, Section 3, Section 4, Section 5, Section 6 and Section 7, we consolidate five research gaps that should guide future work on ISAC-V2X toward 6G and beyond. Table 13 summarizes these gaps, their scope, and representative open questions.

**Figure 17:**
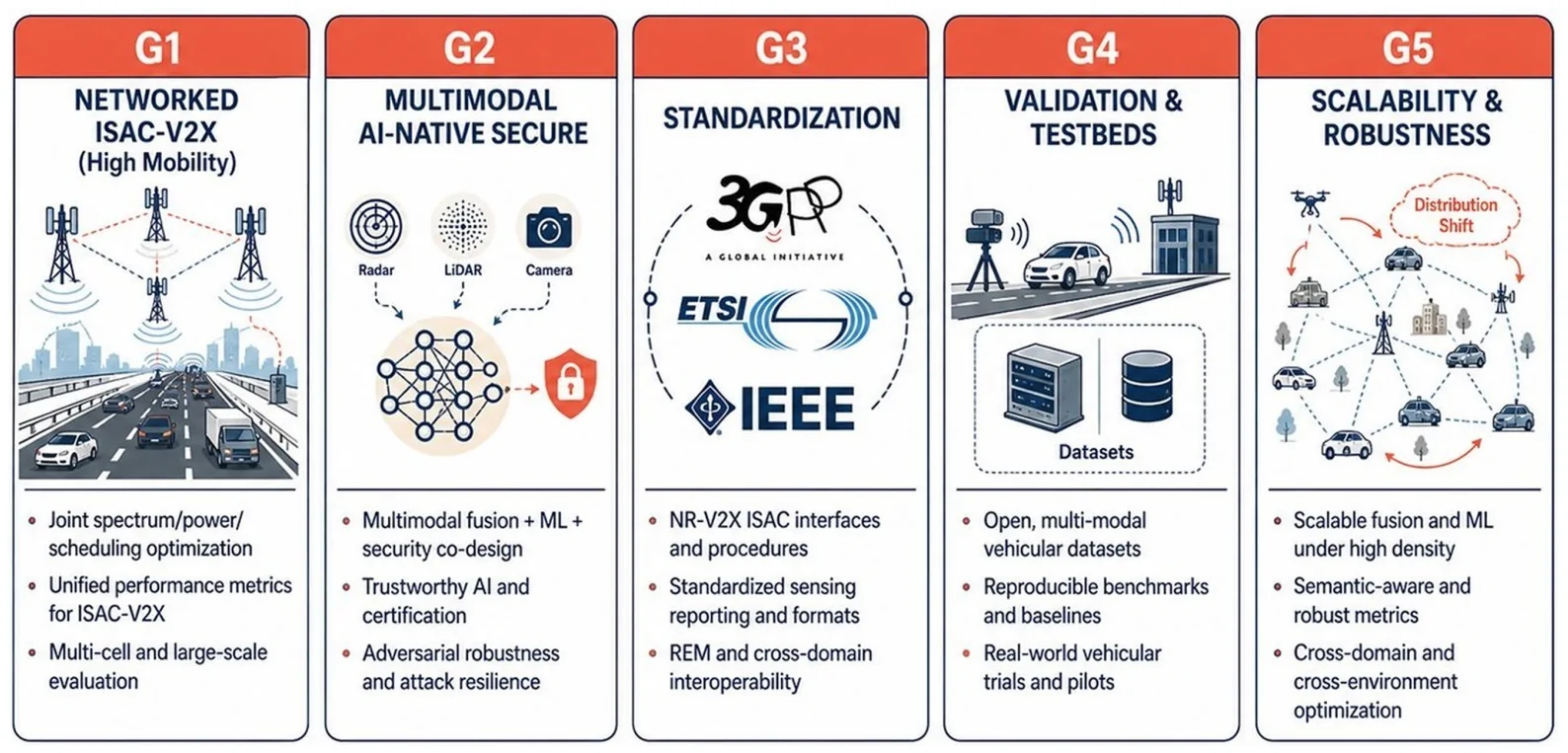
Consolidated research gaps G1–G5 for ISAC-V2X toward 6G.

Figure 17 provides a visual summary of the consolidated gaps and their relationship to the deployment-oriented roadmap discussed later in this section.

**G1: High-mobility networked ISAC-V2X framework.** End-to-end models and design methodologies for networked ISAC-V2X under high mobility are largely missing. Many ISAC works assume point-to-point or quasi-static topologies and do not quantitatively capture the trade-offs between sensing performance, communication QoS, and resource consumption in dense, multi-hop vehicular networks [7,9]. Unified system-level frameworks that jointly optimize spectrum, power, scheduling, and cooperation across many ISAC-enabled vehicles and RSUs under realistic mobility and interference are still absent [5,13]. Closing G1 requires integrated performance metrics, scalable resource and fusion algorithms, and evaluation in dynamic multi-cell and cell-free V2X scenarios.

**G2: Multi-modal AI-native secure ISAC-V2X architecture.** Unified designs that jointly account for sensing, communication, and security in automotive networks are rare, despite the fact that multi-modal fusion and ML for ISAC have developed independently. The majority of systems either tackle multi-modal fusion and V2X traffic engineering separately or concentrate on RF-only ISAC; privacy, robustness to adversarial inputs, and certification of learned models are frequently outside the core design loop [1,8]. In order to assess and manage the impact on dependability, security, and deployment complexity, a unified framework that systematically utilizes multi-modal data and machine learning is required [6,16].

**G3: Standardization and interoperability.** ISAC and V2X are evolving in multiple standards bodies, including 3GPP, ETSI, and IEEE, but aligned specifications for ISAC in V2X remain incomplete. In particular, common procedures for waveform design, resource allocation, sensing reporting, and cooperative sensing have not yet been fully established. Although Release 18/19 and beyond are beginning to address sensing in cellular systems, consistent interfaces for REM exchange, AI/ML-assisted ISAC, and cross-vendor or cross-operator interoperability are still lacking [1,22].

**G4: Validation, testbeds, and reproducible benchmarks.** Large-scale real-world validation of ISAC-V2X remains limited. Existing datasets, including DeepSense 6G and Raymobtime, cover only selected scenarios and sensing modalities, while synchronized multi-modal and multi-node measurements under realistic mobility, interference, and network conditions remain scarce [8,14]. Moreover, common experimental platforms and reproducible evaluation methodologies for jointly assessing communication, sensing, latency, and learning-based functions are still insufficient [23].

**G5: Scalability, robustness, and cross-domain optimization.** Scaling ISAC-V2X to large numbers of vehicles, infrastructure nodes, sensing modalities, and learning agents remains challenging under limited backhaul, mobility-induced distribution shifts, and adversarial conditions. Robustness and interpretability of learned models for safety-critical V2X, together with semantic or task-oriented metrics that jointly reflect communication and sensing performance, remain insufficiently established [25,29]. Moreover, cross-domain optimization that jointly coordinates physical-layer, network, computation, and application-level parameters under latency, energy, and regulatory constraints is still at an early stage [24,158].

### 8.3. Future Research Directions (6G, 7G Roadmap)

Future directions for ISAC-V2X align with 6G and early 7G visions that emphasize native sensing, AI/ML integration, and sustainability. Below, we outline thematic directions and a 2026–2030 roadmap.

**Native sensing and dual-functional air interface.** Access to 6G radio is expected to treat sensing as a first-class service alongside communication, with configurable modes from communication-only to sensing-only and joint ISAC in between [23,37]. Bazzi et al. demonstrate ISAC imaging from CSI using ray tracing for next-generation 6G, illustrating how CSI-based sensing can support environmental mapping [159]. Research should focus on waveform and frame design that support flexible time-frequency-spatial resource split, sensing reference signals compatible with NR-V2X and sidelink, and low-overhead reporting of sensing results (range, velocity, angle, or higher-level features) for cooperative perception and REM [160].

**AI/ML-native design and automation.** Data-driven and learning-based methods will play a central role in channel prediction, beam management, detection, and resource allocation. Celik and Eltawil survey new frontiers in 6G wireless intelligence at the dawn of the generative AI era, with implications for channel and scenario synthesis in ISAC [161]. Directions include the following: scaling federated and transfer learning to vehicular ISAC with non-IID and mobile data; improving robustness and interpretability of learned models for safety certification; and integrating generative AI for data augmentation and scenario synthesis while preserving physical consistency [25]. Zero-touch and intent-based management of ISAC-V2X networks, with closed-loop optimization of sensing and communication, are long-term goals [49].

**Networked and cell-free ISAC.** Multi-BS/RSU cooperation, cell-free architectures, and integrated access and backhaul (IAB) will extend ISAC benefits to coverage-limited and dense urban scenarios. Research should address symbol-level and signal-level fusion under fronthaul and synchronization constraints, joint compression and power allocation for networked ISAC, and integration with MEC for low-latency fusion and control [59,60]. Meng et al. survey UAV-enabled integrated sensing and communication, outlining opportunities and challenges that apply to aerial support for V2X [158]. UAV and non-terrestrial ISAC will complement terrestrial deployments for rural and disaster scenarios; Nomikos et al. review UAV-aided maritime communications and future challenges that parallel those of UAV-assisted V2X [162].

**Security, privacy, and trust.** PLS, covertness, and sensing security must be co-designed with waveform and beamforming. Directions include the following: formal treatment of sensing confidentiality (e.g., limiting what an eavesdropper can infer from the radar waveform); privacy-preserving aggregation of REM and sensing updates; and defenses against adversarial manipulation of learned perception and beam prediction [26,129]. Standardization of security and privacy requirements for ISAC in V2X will be critical for adoption [3].

**Sustainability and energy efficiency.** Future ISAC-V2X systems must balance sensing and communication performance with hardware complexity and energy consumption. Promising directions include energy-efficient waveform and beamforming design, power-aware resource allocation, and low-power operating modes for ISAC-capable RSUs and vehicles [11,54].

From a practical deployment perspective, the transition from research prototypes to large-scale ISAC-V2X systems will depend not only on algorithmic performance but also on hardware cost, processing latency, synchronization accuracy, fronthaul capacity, spectrum regulation, interoperability with legacy V2X, and the availability of validated multi-node testbeds. AI/ML-enabled solutions additionally require robust and certifiable models, representative datasets, and manageable training and inference overhead. These practical constraints suggest a gradual deployment path rather than an immediate transition to fully integrated ISAC-V2X, motivating the phased 2026–2030 roadmap outlined below.

**Expected deployment timeline (2026–2030).** A phased deployment trajectory is anticipated. During 2026–2027, efforts are expected to focus on early cellular sensing capabilities, NR-V2X ISAC trials, multi-modal datasets and benchmarks, and sensing-reporting interfaces [1,22,163]. The 2027–2029 period is likely to emphasize pilot networked ISAC-V2X deployments, multi-BS/RSU cooperation, AI/ML-enabled channel and scenario modeling, and emerging security and privacy mechanisms [3,30,112]. By 2029–2030, larger-scale cell-free and MEC-integrated ISAC-V2X deployments, semantic and task-oriented metrics, and tighter integration of sensing, communication, computation, and control are expected to become more prominent [32,40,164]. Table 14 summarizes these directions across the three time horizons and maps them to G1–G5.

### 8.4. Standardization and Testbed Recommendations

**Standardization.** The standardization trajectory summarized in Section 2.2 shows a progressive transition from service-level ISAC requirements toward system architecture and radio-interface procedures. At the 3GPP service layer, the Release 19 FS_Sensing study resulted in TR 22.837 and was followed by the Stage 1 requirements in TS 22.137 [41,42]. Release 20 extends this work through the Stage 2 architectural study TR 23.700-14 and the RAN1 study TR 38.765 [43,44]. These developments provide an increasingly concrete basis for cellular sensing, but their translation into V2X-specific procedures remains incomplete.

For ISAC-V2X, several standardization priorities therefore remain. First, sensing reference signals and resource patterns should coexist with PSCCH/PSSCH without compromising legacy C-V2X reliability, including suitable time-frequency allocations for in-band and out-of-band sensing [13]. Second, standardized sensing-result reporting and exposure interfaces are required so that raw, feature-level, or decision-level sensing information can be consumed by radio access network (RAN), application, and radio environment map (REM) functions without proprietary extensions [1]. Third, cooperative and multi-node sensing procedures must specify synchronization, measurement aggregation, uncertainty representation, and sensing-result exchange across BSs, RSUs, vehicles, and edge nodes. Fourth, cellular ISAC should interoperate with IEEE 802.11bf WLAN sensing in heterogeneous or multi-RAT deployments [46]. IEEE 802.11bf explicitly structures WLAN sensing around sensing-session setup, measurement, reporting, and termination phases and includes mechanisms for sensing-measurement reporting; these features provide a useful protocol-level reference for future cross-RAT sensing interfaces. Finally, ETSI ITS and C-ITS frameworks should incorporate sensing and REM information into cooperative awareness and automated-driving services [3], while cross-SDO coordination among 3GPP, ETSI, IEEE, and ITU-R is needed to harmonize terminology, sensing-performance requirements, report latency, security, and interoperability [22].

**Figure 18:**
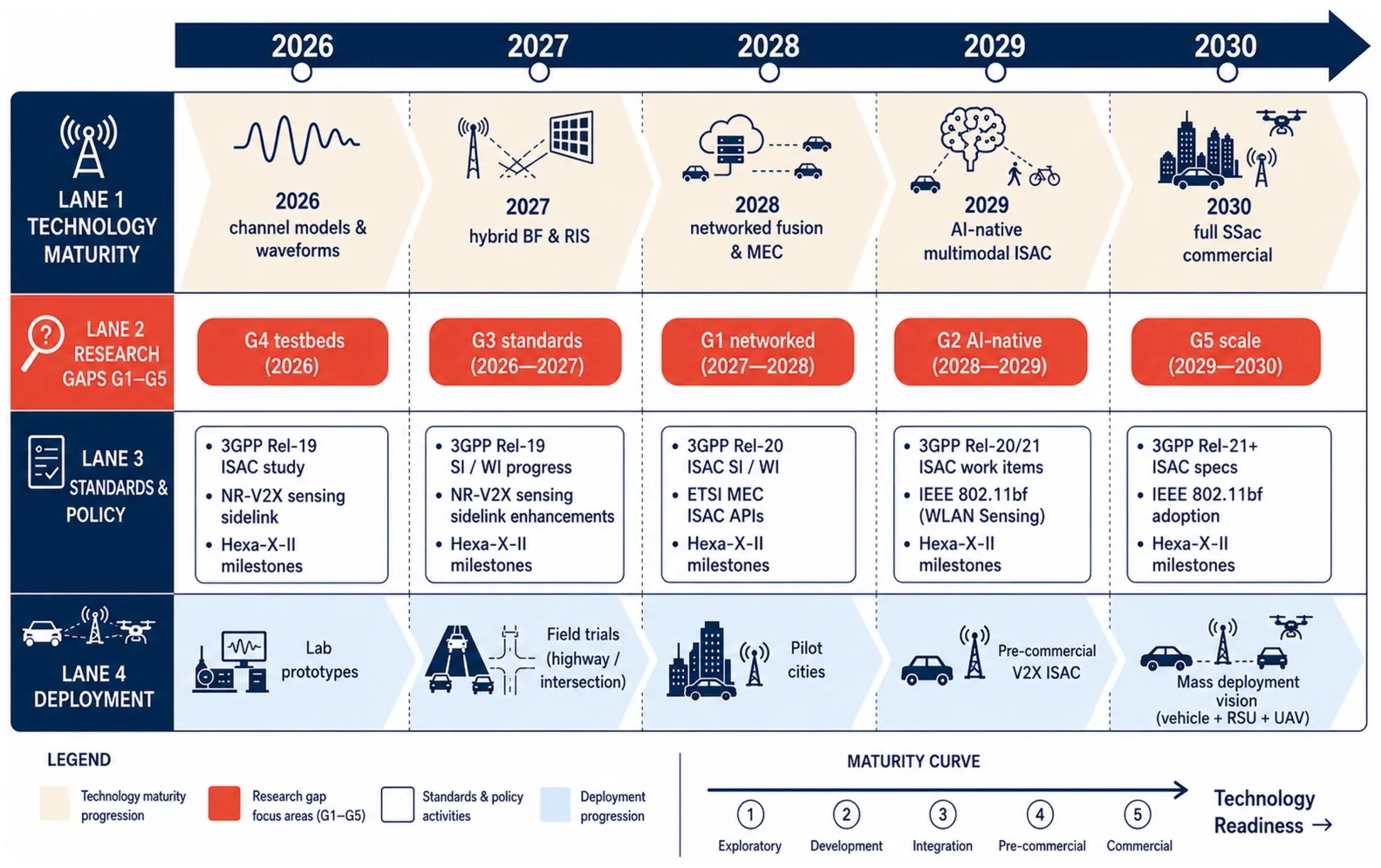
Proposed ISAC-V2X research and deployment roadmap for 2026–2030, linking research gaps, standardization, and deployment maturity.

Table 15 maps the main 3GPP and IEEE sensing milestones to the corresponding ISAC-V2X research directions and highlights the remaining standardization gaps. The standardization and deployment timeline in Figure 18 aligns the above recommendations with the broader ISAC-V2X roadmap.

**Testbeds and benchmarks.** To validate ISAC-V2X under realistic conditions, we recommend the following: (i) *Hardware testbeds* combining software-defined radio platforms or commercial NR-V2X stacks with radar or sensing-capable front-ends, and with configurable RSU/vehicle positions and mobility [23]; (ii) *channel and scenario emulation* using ray-tracing (e.g., NYURay, Remcom) or recorded channels to reproduce high-mobility and multi-path conditions without full outdoor deployment [159]; (iii) *open datasets* that include synchronized RF, LiDAR, camera, and GNSS/IMU from multiple nodes and multiple scenarios (highway, urban, intersection), with clear train/validation/test splits and evaluation metrics [8,14]; (iv) *benchmark suites* that define standard scenarios, metrics (e.g., top-K beam accuracy, detection probability, latency, secrecy rate), and baselines so that new methods can be compared fairly [25]. Zhang et al. design and evaluate a joint sensing and communication system for 5G mmWave-enabled connected and automated vehicles, providing an early example of system-level evaluation relevant to future testbeds [53]. Collaboration between academia, industry, and regulators on shared testbeds and datasets will accelerate progress and reduce duplication.

### 8.5. Research Opportunities and Lessons Learned

**Research opportunities and outstanding directions.** Key opportunities remain in characterizing the fundamental limits of multi-node and multi-target ISAC-V2X, jointly optimizing latency, reliability, and sensing performance, and developing interpretable and certifiable learning-based solutions for safety-critical operation [34,61,68,77,97]. Further challenges concern spectrum and regulatory alignment, as well as cost-effective and backward-compatible deployment of ISAC-capable infrastructure and vehicles [3,54,165]. These issues require closer integration of PHY design, learning, regulation, and practical implementation.

**Lessons learned.** The surveyed literature shows that sensing and communication provide the greatest benefit when jointly designed, but their integration introduces unavoidable trade-offs among rate, sensing accuracy, secrecy, privacy, and computational overhead. Networked and multi-modal operation is essential for scalable 6G V2X, while open datasets, reproducible benchmarks, and security-by-design remain critical for practical adoption [116,120]. These observations reinforce the need for system-level rather than isolated optimization of ISAC-V2X functions.

### 8.6. Concluding Remarks

This survey has provided a unified view of ISAC-V2X spanning physical-layer design, networked operation, security and privacy, multi-modal intelligence, and deployment considerations for 6G vehicular systems. The five consolidated gaps G1–G5 define a research agenda covering high-mobility networked operation, multi-modal AI-native design, standardization, experimental validation, and scalability, whose resolution will require coordinated advances in physical-layer design, network architecture, security, interoperable standards, and reproducible testbeds. As 6G standardization and trials progress, ISAC-V2X is expected to evolve from single-link demonstrations toward deployable, multi-node, and AI-native vehicular systems, with the roadmap and recommendations developed in Section 8 providing a structured path toward practical integrated sensing and communication for V2X.

## Figures and Tables

**Table 1:** Comparison of representative surveys related to ISAC and 6G V2X. Symbols: ✓, covered; △, partially covered; ☓, not covered.

Survey	ISAC	V2X	Security	AI/ML	Multi-Modal	Networked ISAC	Deployment/Roadmap
Aldirmaz-Colak et al. [1]	✓	△	✓	✓	△	△	△
Rodriguez-Pineiro et al. [3]	△	✓	△	✓	△	△	✓
Tan and Zhu [8]	△	✓	△	✓	✓	△	✓
Matsumine et al. [15]	✓	☓	✓	△	☓	△	☓
Cheng et al. [7]	✓	✓	△	△	☓	△	△
Wang et al. [9]	✓	△	△	△	☓	△	✓
Demirhan and Alkhateeb [6]	✓	△	△	✓	△	△	△
Ma et al. [18]	✓	✓	△	△	△	△	△
Zhong et al. [19]	✓	✓	☓	△	△	△	△
Wei et al. [20]	✓	☓	☓	△	☓	☓	△
Tan et al. [21]	☓	✓	☓	△	△	☓	△
Zhang et al. [25]	✓	△	△	✓	△	△	✓
Zhu et al. [26]	✓	△	✓	✓	△	△	✓
Wei et al. [27]	✓	△	☓	△	☓	△	△
Magbool et al. [28]	✓	△	△	△	☓	△	△
Sheraz et al. [29]	△	△	✓	✓	△	△	✓
Wen et al. [30]	✓	△	☓	△	☓	✓	✓
This survey	✓	✓	✓	✓	✓	✓	✓

**Table 2:** Summary comparison of the main ISAC-V2X approaches.

Approach	Main Advantages	Main Limitations	Suitable ISAC-V2X Scenarios
OFDM-based ISAC [9,35]	Mature signal processing, flexible time-frequency resource allocation, and strong compatibility with existing cellular/V2X infrastructure	Sensitivity to high Doppler and intercarrier interference in rapidly time-varying vehicular channels	Urban V2I/V2X, roadside sensing, and deployments prioritizing NR compatibility
OTFS-based ISAC [35,52]	Strong robustness to delay-Doppler variations and improved operation under high mobility	Higher receiver processing and detection complexity compared with conventional OFDM	Highway, high-speed V2X, and severe-Doppler environments
DFRC/Joint Waveform Design [7,9]	Direct reuse of spectrum, waveform, and hardware for communication and sensing	Coupled sensing–communication objectives and non-convex waveform optimization	Short-range vehicular sensing, platooning, and joint radar–communication links
Hybrid/Massive-MIMO Beamforming [53,54]	High directional gain, spatial multiplexing, and simultaneous support for users and sensing targets	Beam alignment, RF-chain cost, hardware constraints, and mobility-induced tracking overhead	mmWave V2X, highway links, and directional RSU-to-vehicle operation
RIS/STAR-RIS-Assisted ISAC [55,56,57]	Coverage extension, blockage mitigation, controllable propagation, and improved sensing/communication performance	Channel-estimation overhead and large-dimensional joint beamforming/phase optimization	Urban NLoS, blockage-prone intersections, and infrastructure-assisted V2X
NOMA/RSMA-Assisted ISAC [11,58]	Improved spectral efficiency, flexible interference management, and multi-user connectivity	SIC/rate-splitting complexity and sensitivity to imperfect CSI and resource allocation	Dense multi-user V2X and spectrum-constrained vehicular networks
Networked/Cooperative ISAC [59,60]	Extended sensing coverage, spatial diversity, cooperative perception, and improved localization	Synchronization, fronthaul/backhaul overhead, interference coordination, and scalability challenges	Intersections, dense urban traffic, multi-RSU/multi-BS sensing, and cell-free V2X
AI/ML-Enabled ISAC [6,8,61]	Adaptive beam prediction, channel estimation, target processing, resource allocation, and fast online inference	Training-data requirements, robustness/generalization issues, model complexity, and explainability concerns	Highly dynamic environments, predictive beam management, multi-modal perception, and adaptive V2X

**Table 3:** Representative channel models for vehicular ISAC.

References	Frequency	Scenario	Mobility	Model Type	Key Parameters
[64,82]	28 GHz	Vehicular ISAC (front, left, right)	Dynamic (birth–death MPCs)	Stochastic TDL	S-MPCs, C-MPCs, lifetimes, power/delay evolution
[27,70]	28 GHz	Indoor ISAC	Quasi-static	Shared-cluster stochastic	Shared/non-shared clusters, SD, joint clustering
[27,68]	Survey	General ISAC	Various	Deterministic + statistical	Target/clutter RCS, one-way vs. two-way, 3GPP-style
[66,71]	mmWave	6G massive MIMO ISAC	Non-stationary	3D GBSM, BDCM	Angular/delay spread, sensing–comm. correlation
[66,71]	mmWave	AHRIS-ISAC	Aerial mobility, jitter	3D non-stationary GBSM	Jitter, forward/backward scattering, sensing–comm. correlation
[67,69]	77 GHz	Automotive JCRS	Vehicular	Measurement-based (PMCW-CDMA)	RF hardware, automotive JCRS
[37,67]	Sub-6/mmWave	Vehicular (OTFS)	High (Doppler-resistant)	OTFS-based ISAC	Delay-Doppler domain, Doppler resilience
[64,82]	OFDM MIMO	General ISAC (clutter)	Time-varying	Transceiver design (clutter suppression)	Clutter suppression, robust transceiver
[83,84]	THz	RIS-ISAC	Configurable	Robust beamforming	Imperfect CSI, THz propagation

**Table 4:** Representative PHY techniques (waveform and beamforming) for ISAC-V2X.

Ref.	Cat.	Type/Waveform	Key Features or Objective	V2X Relevance or Constraint
[85,86]	W	OFDM	Subcarrier/power allocation, PAPR reduction	5G NR compatibility, flexible resource use
[85,86]	W	OFDM	Echo model, block FFT, MI/rate trade-off	Standard-aligned, multi-carrier sensing
[37,67]	W	OTFS	Beamforming from topology, uplink pilot reduction	High-mobility, low pilot overhead
[37,67]	W	OTFS	DD correlation, complexity vs. OFDM	JRC architectures, high Doppler
[52,60]	W	OTFS	Cell-free MIMO, power allocation, SOC	Cell-free ISAC, QoS + sensing SNR
[84,88]	W	OFDM + RHS/RIS	Holographic + passive beamforming, max-min radar SINR	NLoS, wideband DFRC
[89,90]	W	Active ISAC	Online learning, waveform optimization	Time-varying objectives
[88,90]	W	CoMP	Asynchronous errors, joint design	Multi-BS robustness
[39,91]	B	Hybrid (HAD)	STMB, V2X DFRC, max. rate	Radar SINR per target
[65,91]	B	Hybrid (HAD)	VBA-ISAC, trade-off (S&C)	Power, analog phase
[39,65]	B	Hybrid	IoV DFRC mmWave, beam alignment	RF chains, hardware
[92,93]	B	Digital	Multi-target, multi-user, weighted S&C	Sum power
[84,93]	B	Hybrid + RIS	mmWave ISAC, joint BF + RIS phases	Power, RIS modulus
[84,94]	B	Joint BF + waveform	RIS-assisted MIMO, robust S&C	Channel uncertainty
[94,95]	B	Full-duplex	Joint comm. + sensing beamformer design	Self-interference
[86,95]	B	Survey	Hybrid/digital/analog comparison	Complexity vs. performance

Cat.: W = waveform, B = beamforming.

**Table 5:** Qualitative comparison of computational complexity and scalability of representative ISAC-V2X approaches.

Approach	Computational Complexity	Scalability	Main Complexity Driver	Practical Vehicular Suitability
OFDM-based ISAC [35]	Low-Moderate	High	FFT-based sensing and resource allocation	Strong compatibility with NR-V2X and practical for real-time deployment
OTFS-based ISAC [35,52]	Moderate-High	Moderate	Delay-Doppler processing and detection	Attractive for high-mobility and severe Doppler environments
Digital beamforming [53,54]	High	Limited-Moderate	Large-dimensional precoding and RF-chain count	High performance but costly for large vehicular arrays
Hybrid beamforming [53,54]	Moderate	High	Joint analog–digital precoding	Suitable compromise for mmWave massive-MIMO V2X
RIS/STAR-RIS ISAC [55,56,57]	High	Moderate	Joint beamforming and large-dimensional phase optimization	Promising for blocked and NLoS vehicular scenarios
NOMA/RSMA ISAC [11,58]	Moderate-High	Moderate	SIC, rate splitting, power and beam allocation	Useful in dense multi-user vehicular networks
Networked/cell-free ISAC [59,60]	High-Very High	Challenging	Synchronization, fronthaul, multi-node coordination	Suitable for cooperative sensing if infrastructure support is available
AI/ML-enabled ISAC [61,97]	High training/Low inference	High	Model training and distributed updates	Attractive for low-latency adaptation after offline training

**Table 6:** Comparison of PLS methods for ISAC-V2X.

Reference/Method	Scenario	Objective	Key Constraints	Solution Approach
Yao et al. [26,116]	UAV, dual eavesdropper	Max. avg. secrecy rate	Sensing gain at target, sensing security at Eve, power, trajectory	AO + SCA + SDR
Liu et al. [114,119]	RIS, target as Eve	Max. sum secrecy rate	Beampattern matching, power	SAC-DRL (beamforming + AN + RIS)
Wang et al. [106,120]	C-NOMA-SWIPT, imperfect Eve CSI	Min. transmit power	Data rates, eavesdrop rates, echo SINR	S-Procedure + SDR + BCD
Wu et al. [116,117]	UAV, mobile Bob	Max. real-time secrecy rate	Speed, trajectory	EKF tracking + SCA trajectory
Xu et al. [118,121]	IRS-UAV	Anti-jamming + secrecy	Echo SINR, power	Joint active/passive beamforming + deployment
Deng et al. [116,120]	UAV, covert	Max. avg. covert rate	Detection prob. at wardens, sensing gain, power, speed	BCD (beamforming + trajectory)
Shu et al. [122,123]	ISAC + ISRJ	Min. MUI	Target/jamming mainlobe, sidelobe, power	ADMM (waveform + matched/mismatched filter)

**Table 7:** Taxonomy of attacks in ISAC-V2X.

Attack Type	Communication Impact	Sensing/REM Impact	Example Defenses
Eavesdropping	Intercept data	-	Secrecy rate, AN, beamforming [26,116,119]
Sensing eavesdropping	-	Extract target/env. info	Sensing security constraint, beampattern null at Eve [116,120]
Jamming	Degrade SINR, block link	False targets (e.g., ISRJ), mask echoes	Joint waveform + receive filter [118,122]
Spoofing	Impersonate node, steal resources	-	Authentication, ROC-based detection [33,47]
Manipulation	-	Faulty REM, wrong sensing	Integrity checks, PLS for sensing [3,33]
Disruption	Broader than jamming	Flood sensing/processing	Detection, MEC/edge security [33,47]
RIS abuse	Boost Eve/relay jammer	-	RIS access control, monitoring [114,121]

**Table 8:** Comparison of networked ISAC-V2X architectures.

Architecture	Topology	Fusion Level	Fronthaul Demand	Synchronization	Key References
Multi-BS cooperative (ISAC-MCS)	Macro/micro BSs + MEC + cloud	Data/signal/symbol	Medium (data) to high (signal)	Symbol-level to sub-symbol	[59,130,139]
Two-BS cooperative (MI-based)	2 BSs, central unit	N/A (joint transmission)	Low (beamforming only)	Coherent TX/RX	[59,131]
CoMP ISAC (async)	Multiple TRPs, 1 RX	Central estimation	Medium	Joint estimation of asynchrony + location	[88,90]
RA-enabled multi-BS	Multiple BSs with rotatable arrays	Beampattern matching	Low (angles + beamforming)	Coherent	[90,132]
Cell-free UAV ISAC	Multiple UAVs as APs	EKF tracking at CP	Medium (channel/state)	LoS, position-based	[81,135]
Cell-free OTFS ISAC	Multiple TX/RX APs, C-RAN	Central detection	High (echoes or compressed)	OTFS sync	[52,60]
Networked ISAC (fronthaul-limited)	CP + M TXs + 1 RX	Central CRLB-based	Capacity-limited compression	Coherent after correction	[52,60]

**Table 9:** Resource allocation methods for networked ISAC-V2X.

Method/Reference	Objective	Decision Variables	Constraints	Solution Approach
J-FCPA [52,60]	Min. transmit power	Power, compression noise	Fronthaul, SICNR, CRLB	Closed-form compression; SCA for power
Queue-aware [77,143]	Utility (communication + radar)	Time/spectrum allocation	Queue stability	Lyapunov optimization
Low-latency V2X [77,143]	Min. sensing latency	Repetitions, block length, threshold	Detection probability, SINR	B&B for MINLP
Cell-free SC [52,135]	Max. sensing SNR	Power allocation	Per-UE QoS, per-AP power	Convex optimization
UAV cell-free [81,135]	Max. sum rate	Beamforming, trajectory, association	Sensing, power	BCD + FP
EAOM IoV [111,140]	Min. ORT	Offloading, scheduling, resources	DT, slicing	SQDDPG (Shapley-DDPG)
Cloud–edge IoV [140,141]	Min. latency, max. ESP profit	Offloading, compute allocation	Capacity, pricing	K-means++ + DDQN
UAV V2X offloading [111,141]	Min. energy + latency	Offload decision, bandwidth, CPU	Power, QoS	SCA

**Table 10:** Multi-modal fusion taxonomy in ISAC-V2X.

Fusion Level	Modalities	Typical Use Case	References
Raw/data	RF + LiDAR histograms + GNSS	Beam selection (e.g., Raymobtime)	[8,14]
Feature	ResNet RGB + position; radar + CSI	Beam prediction, channel estimation	[5,8,86]
Decision	Classifier outputs per modality	Strong classifier via weighted fusion	[5,9]
Hybrid	GNSS + LiDAR + RGB; radar + camera	V2N/V2V beam, blockage prediction	[8,14]

**Table 11:** Comparison of ML methods for ISAC-V2X (channel/beam and sensing).

Method	Channel/Beam Application	Sensing Application	Label Need	Complexity	References
Supervised (DNN/CNN)	Beam prediction, channel estimation	Target classification, detection	High	Medium	[48,61,146]
Unsupervised	Waveform design (e.g., rate/SINR loss)	Clustering, representation	None	Low-medium	[61,153]
RL/DRL	Resource allocation, trajectory	-	None	High	[119,147,148]
Transfer learning	Fast adaptation to new scenarios	Few-shot detection/beam	Low	Medium	[25,61]
Federated learning	Distributed beam/channel model	Distributed sensing model	Per-node	Medium	[56,61,154]
GenAI (GAN/VAE)	Channel/data augmentation	Synthetic radar/vision data	Optional	High	[29,35,155]

**Table 12:** Mapping of the reviewed literature findings to the consolidated research gaps.

Survey Section	Main Findings from the Reviewed Literature	Remaining Limitations	Related Gaps
Section 2, Section 3 and Section 4	ISAC architectures, high-mobility channel models, waveform design, beamforming, RIS, and joint resource allocation provide strong link-level sensing–communication gains.	Performance evaluation remains fragmented across sensing and communication metrics, while many optimization frameworks become difficult to scale under mobility, interference, and multi-node operation.	G1, G5
Section 5	PLS, secure beamforming, RIS-assisted security, privacy-aware processing, and attack mitigation improve communication and sensing resilience.	Security, sensing confidentiality, privacy, and AI robustness are still rarely incorporated within a unified vehicular ISAC design and lack common evaluation frameworks.	G2, G3, G5
Section 6	Multi-BS/RSU cooperation, cell-free architectures, MEC, and joint resource allocation improve coverage, sensing diversity, and network-level coordination.	Unified sensing–communication metrics, scalable multi-node cooperative optimization, synchronization/fronthaul management, and realistic experimental validation remain unresolved.	G1, G4, G5
Section 7	Multi-modal sensing and AI/ML enable beam prediction, channel estimation, tracking, fusion, and adaptive resource management.	Unified multi-modal architectures, robust and certifiable learning, representative datasets, privacy protection, and scalable distributed inference remain limited.	G2, G4, G5

**Table 13:** Consolidated research gaps (G1–G5) for ISAC-V2X.

Gap	Scope	Representative Open Questions
G1	High-mobility networked ISAC-V2X	Joint optimization of spectrum, power, scheduling, and cooperation across many nodes; unified metrics for sensing and communication in multi-cell/cell-free V2X
G2	Multi-modal AI-native secure ISAC-V2X	Unified architecture for multi-modal fusion + ML + security; certification and robustness of learned perception and beam prediction
G3	Standardization and interoperability	3GPP/ETSI/IEEE interfaces for sensing reporting and REM; coexistence of ISAC with NR-V2X and sidelink
G4	Validation and testbeds	Open multi-node, multi-modal datasets; hardware testbeds; reproducible benchmarks and evaluation protocols
G5	Scalability, robustness, cross-domain	Scaling fusion and ML to many nodes/modalities; distribution shift and adversarial robustness; semantic/task-oriented metrics

**Table 14:** Future research directions and roadmap (2026–2030).

Direction	Short-Term (2026–2027)	Mid-Term (2027–2029)	Long-Term (2029–2030)	Related Gaps
Native sensing air interface	Release 19 sensing features; NR-V2X ISAC trials	Sensing reference signals; reporting formats	Flexible S&C resource split; 7G studies	G3
AI/ML-native design	Datasets; FL/GenAI for channel/scenario	Robustness; interpretability; zero-touch	Certified learning-based ISAC	G2, G5
Networked/cell-free ISAC	Multi-BS cooperation; symbol-level fusion	Fronthaul-aware compression; MEC integration	Cell-free + UAV/NTN ISAC at scale	G1, G4
Security and privacy	PLS and sensing security co-design	Standards for sensing privacy; adversarial defenses	Trustworthy ISAC-V2X	G2, G3
Testbeds and benchmarks	Open datasets; emulation	Hardware testbeds; benchmark suites	Reproducible evaluation across industry	G4

**Table 15:** Key standardization milestones and corresponding ISAC-V2X research gaps.

Period/Release	Milestone	Related Research Direction	Remaining ISAC-V2X Gap
2022–2023/Rel. 19	TR 22.837 and TS 22.137	ISAC use cases, sensing KPIs, and service requirements	V2X-specific sensing KPIs and sidelink procedures remain limited
2025–2026/Rel. 20	TR 23.700-14	Networked sensing, multi-node cooperation, edge processing, and sensing-result exposure	Vehicular multi-BS/RSU cooperation and interoperable sensing-result exchange remain open
2025–2026/Rel. 20	TR 38.765	NR sensing signals, measurements, resource design, and sensing-assisted radio operation	High-mobility and NR-V2X sidelink sensing procedures require further specification
2025	IEEE Std 802.11bf-2025	Sensing-session management, measurements, reporting, ranging, and motion sensing	Cellular-WLAN sensing interoperability and cross-RAT sensing exchange remain open
2026–2030	Academic ISAC-V2X research	Cooperative sensing, multi-modal AI, predictive mobility, RIS, and edge intelligence	Research capabilities remain ahead of interoperable V2X sensing interfaces and validation procedures

## Data Availability

No new data were created or analyzed in this study. Data sharing is not applicable to this article.

## Abbreviations

The following abbreviations are used in this manuscript:

**Abbr.**	**Meaning**	**Abbr.**	**Meaning**
3GPP	3rd Generation Partnership Project	mmWave	Millimeter-wave
6G	Sixth-generation	MPC	Multi-path component
ACR	Achievable covert rate	MUI	Multi-user interference
ADMM	Alternating direction method of multipliers	NLoS	Non-line-of-sight
AI	Artificial intelligence	NOMA	Non-orthogonal multiple access
AN	Artificial noise	NR	New radio
AO	Alternating optimization	NR-V2X	NR vehicle-to-everything
AP	Access point	NTN	Non-terrestrial network
B5G	Beyond 5G	OFDM	Orthogonal frequency-division multiplexing
BCD	Block coordinate descent	OMA	Orthogonal multiple access
BS	Base station	OTFS	Orthogonal time frequency space
CAD	Connected autonomous driving	PAPR	Peak-to-average power ratio
C-ITS	Cooperative intelligent transport systems	PC5	3GPP sidelink interface
CoMP	Coordinated multi-point	PHY	Physical layer
CR	Cooperative communication rate	PLS	Physical-layer security
CRB	Cramér–Rao bound	PSCCH	NR sidelink control channel
CRLB	Cramér–Rao lower bound	PSSCH	NR sidelink data channel
CSI	Channel state information	QoS	Quality of service
C-V2X	Cellular V2X	RCC	Radar–communication coexistence
DFRC	Dual-function radar–communication	RCS	Radar cross-section
DL	Deep learning	REM	Radio environment map
DRL	Deep reinforcement learning	RF	Radio frequency
DSRC	Dedicated short-range communications	RIS	Reconfigurable intelligent surface
EKF	Extended Kalman filter	RL	Reinforcement learning
eMBB	Enhanced mobile broadband	RSMA	Rate-splitting multiple access
FL	Federated learning	RSU	Roadside unit
FMCW	Frequency-modulated continuous wave	SCA	Successive convex approximation
FP	Fractional programming	SDR	Semidefinite relaxation
GBSM	Geometry-based stochastic model	SIC	Successive interference cancellation
GNSS	Global navigation satellite system	SINR	Signal-to-interference-plus-noise ratio
HAD	Hybrid analog–digital beamforming	SNR	Signal-to-noise ratio
IMU	Inertial measurement unit	SOP	Secrecy outage probability
IoT	Internet of Things	SR	Sensing rate
IoV	Internet of Vehicles	SSaC	Symbiotic sensing and communications
ISAC	Integrated sensing and communication	STAR-RIS	Simultaneously transmitting and reflecting RIS
ISFFT	Inverse symplectic FFT	STMB	Single-target multi-beam
ISRJ	Interrupted-sampling repeater jamming	SWIPT	Simultaneous wireless information and power transfer
JCAS	Joint communication and sensing	THz	Terahertz
JCRS	Joint communication and radar system	UAV	Unmanned aerial vehicle
JRC	Joint radar–communication	UE	User equipment
KPI	Key performance indicator	URLLC	Ultra-reliable low-latency communication
LiDAR	Light detection and ranging	Uu	Cellular uplink/downlink interface
LoS	Line-of-sight	V2I	Vehicle-to-infrastructure
LTE	Long-term evolution	V2N	Vehicle-to-network
MEC	Multi-access edge computing	V2V	Vehicle-to-vehicle
MFR	Minimum fairness rate	V2X	Vehicle-to-everything
MIMO	Multiple-input multiple-output	VRU	Vulnerable road user
MISO	Multiple-input single-output	WEE	Weighted energy efficiency
ML	Machine learning	WLAN	Wireless local area network
mMTC	Massive machine-type communication		
